# *Rhizobium etli* CFN42 proteomes showed isoenzymes in free-living and symbiosis with a different transcriptional regulation inferred from a transcriptional regulatory network

**DOI:** 10.3389/fmicb.2022.947678

**Published:** 2022-10-13

**Authors:** Hermenegildo Taboada-Castro, Jeovanis Gil, Leopoldo Gómez-Caudillo, Juan Miguel Escorcia-Rodríguez, Julio Augusto Freyre-González, Sergio Encarnación-Guevara

**Affiliations:** ^1^Proteomics Laboratory, Program of Functional Genomics of Prokaryotes, Center for Genomic Sciences, National Autonomous University of Mexico, Cuernavaca, Morelos, Mexico; ^2^Division of Oncology, Section for Clinical Chemistry, Department of Translational Medicine, Lund University, Lund, Sweden; ^3^Regulatory Systems Biology Research Group, Program of Systems Biology, Center for Genomic Sciences, National Autonomous University of Mexico, Mexico City, Mexico

**Keywords:** transcriptional regulatory network, *Rhizobium etli*, nitrogen fixation, *Phaseolus vulgaris*, free life, proteomics, Isoenzymes

## Abstract

A comparative proteomic study at 6 h of growth in minimal medium (MM) and bacteroids at 18 days of symbiosis of *Rhizobium etli* CFN42 with the *Phaseolus vulgaris* leguminous plant was performed. A gene ontology classification of proteins in MM and bacteroid, showed 31 and 10 pathways with higher or equal than 30 and 20% of proteins with respect to genome content per pathway, respectively. These pathways were for energy and environmental compound metabolism, contributing to understand how *Rhizobium* is adapted to the different conditions. Metabolic maps based on orthology of the protein profiles, showed 101 and 74 functional homologous proteins in the MM and bacteroid profiles, respectively, which were grouped in 34 different isoenzymes showing a great impact in metabolism by covering 60 metabolic pathways in MM and symbiosis. Taking advantage of co-expression of transcriptional regulators (TF’s) in the profiles, by selection of genes whose matrices were clustered with matrices of TF’s, Transcriptional Regulatory networks (TRN´s) were deduced by the first time for these metabolic stages. In these clustered TF-MM and clustered TF-bacteroid networks, containing 654 and 246 proteins, including 93 and 46 TFs, respectively, showing valuable information of the TF’s and their regulated genes with high stringency. Isoenzymes were specific for adaptation to the different conditions and a different transcriptional regulation for MM and bacteroid was deduced. The parameters of the TRNs of these expected biological networks and biological networks of *E. coli* and *B. subtilis* segregate from the random theoretical networks. These are useful data to design experiments on TF gene–target relationships for bases to construct a TRN.

## Introduction

*Rhizobium etli* CFN42 is a soil bacterium classified as an alpha-proteobacterium able to establish a symbiotic relationship with leguminous plants, and this faculty is shared with some members of the beta-proteobacterium group (Andrews and Andrews 2017; Lardi and Pessi 2018; Dicenzo et al. 2019). When the seeds of the bean plant *Phaseolus vulgaris* germinate with *R. etli* CFN42 in a tropical soil, chemical communication starts in the roots to establish a symbiotic relationship. In this process, the root of the plant develops an infection thread through which the bacteria are internalized and travel with some duplications, while the root cortex gives rise to the nodule primordium. When the infection thread reaches the nodule cells, the bacteria are released into organelle-like membranes derived from the host cell plasmalemma called the symbiosome in the nodule. In this stage, *Rhizobium* has some additional duplications, but very soon they stop growing and become pleomorphic, and symbiotic biological nitrogen fixation (SNF) starts (Rascio and La Rocca 2013; Dicenzo et al. 2019). These pleomorphic bacteria, called bacteroids, carry out the expensive reduction of atmospheric N_2_ to ammonium, which is exported to the plant cell, in an exchange of carbon compounds supplied from the photosynthesis of the plant cells. This photosynthate is metabolized by the bacteroid to sustain the SNF (Rascio and La Rocca 2013). Rhizobial inoculants are inexpensive alternatives to environmentally polluting industrial nitrogen fertilizers, with significant impacts on the livelihood of the community. Replacing the use of chemical fertilizers with SNF is a relevant strategy against global warming, favoring sustainable agriculture for the production of grains for human consumption, feed and pasture species (Oldroyd et al. 2011; Ferguson et al. 2019). Proteomic studies on symbiosis have been reported (Larrainzar and Wienkoop 2017; Lardi and Pessi 2018; Liu et al. 2018; Khatabi et al. 2019) and a search for binding sites (motifs) of transcriptional regulators (TFs; Fischer 1994; Tsoy et al. 2016; Rutten and Poole 2019). Now, the first study on O_2_-dependent regulation of the SNF by extending known motifs by bioinformatic methods was performed to establish a regulatory network of proteins and global TFs considering 50 genomes of the Alphaproteobacteria by extending known motifs recognized by the TFs based on a phylogenetic footprinting approach, i.e., the *nifA-rpoN* regulon of nitrogen fixation in the Alphaproteobacteria group was searched, and the deduced matrix from the motifs of the TFs inferred with a strict *p*-value was used to scan with the Run profile tool in the Regpredict site (Novichkov et al. 2010). Using the same *p*-value to search for additional regulon members, 95 operons with potential NifA-binding sites comprising 280 genes were found in Alphaproteobacteria (Tsoy et al. 2016). The NifA-RpoN regulon of *R. etli* CFN42 was determined experimentally and with bioinformatic methods; it consisted of 120 genes, which indicates that the aforementioned study of the NifA regulon in Alphaproteobacteria is highly conservative, highlighting that genes not directly related to nitrogen fixation were found (Salazar et al. 2010), as was also observed (Tsoy et al. 2016). Based on the biological functions resulting from protein interactions, the symbiosis interactome of *Sinorhizobium meliloti* with its host plants was proposed by computational methods, which is composed of 440 proteins involved in 1041 unique interactions (Rodriguez-Llorente et al. 2009).

These data show that the symbiotic nitrogen fixation regulatory circuitry is suspected to be complex. Most of the symbiotic stage protein profiles in the cited literature include TFs (see above), but efforts are needed to infer the genetic circuitry between TFs and the proteins for each profile. We need to take advantage of the co-expression of TFs with potential target proteins in a proteomic profile due to the enrichment of common motif sites involved in the transcriptional regulation of these genes (Van Helden et al. 1998; McGuire et al. 2000; Aerts et al. 2003; Ihuegbu et al. 2012), considering autoregulation of the TFs and that they are involved in the transcriptional regulation of proteins of their respective profile.

We recently constructed the RhizoBindingSites database^1^ (Taboada-Castro et al. 2020), a DNA-motif site collection based on the inferred motifs from each gene recognizing a site in its own promoter region, covering nine representative genomes of the taxon Rhizobiales. This algorithm aligns all the upstream regions of the orthologous genes per gene per genome to search for pairs or conserved position-specific trinucleotides (dyads) to define the motifs (Defrance et al. 2008). These dyads represented in a position-specific scoring matrix (PSSM; Hertz et al. 1990) were used to scan all the genes of a respective genome. These output data per gene per genome were fractionated at low, medium, and high stringency of *p*-value ranges. These data are used to match protein profiles from experimental or theoretical data to predict transcriptional regulatory networks at the desired *p*-value, or using the “auto” option, in which in each round the algorithm selects the data with the lowest *p*-value (high stringency) by searching from the highly strict to low strict data in the proper genome, assuring the output data are with the highly strict *p*-value as possible (Taboada-Castro et al. 2020). This database contains from one to five conserved motifs represented in matrices per gene that have different significance. At the moment, it is not clear which motifs conserved in a gene are directly involved in the recognition of the ARN polymerase and which *p*-value corresponds to the biological action of the TF. The lowest *p*-values are generally used (Tsoy et al. 2016). Inferred data on transcriptional regulation in the SNF are important to accelerate experiments on transcriptional regulation to define TF gene targets, which are basic components of a regulatory network (Resendis-Antonio et al. 2005, 2012; Tsoy et al. 2016).

For *R. etli* CFN42, a systems biology of the metabolic activity during SNF integrating proteome and transcriptome data was used, i.e., 415 proteins and 689 upregulated genes, respectively. From this, 292 unique proteins were identified. This constraint-based model was used to simulate metabolic activity during SNF, and 76.83% of enzymes were justified. The metabolic pathways sustaining SNF activity were discussed compared with aerobic growth in succinate ammonium minimal medium (MM; Resendis-Antonio et al. 2011).

In this work, the study of the SNF proteome of *R. etli* CFN42 was revised with the same experimental conditions (Resendis-Antonio et al. 2011), comparing the aerobic growth at 6 h in MM and the symbiosis at 18 days post-inoculation (dpi). A total of 1730 proteins were identified in MM and 730 in bacteroids; compared to the first report (Resendis-Antonio et al. 2011), it contains 2.5 times more proteins identified in symbiosis. Similar pathways supporting the SNF and a role of the different genome compartments were identified, and new pathways related to adaptation to environmental conditions were described. A new study of the vicinity of the genes expressed in the genome of *R. etli* CFN42 showed specific zones for growth in MM and bacteroid. The chromosome has more genes for growth in MM than in bacteroid, which were more scattered, while for the SNF, the symbiotic plasmid d (p42d) has more genes than for growth in MM. The MM and bacteroid proteome profiles included 127 and 62 TFs, respectively. A potential transcriptional regulatory network for MM and bacteroid was constructed using the RhizoBindingSites database and the prediction of regulatory network approach with the auto option, proposing on average 87% of TF gen-target relationships with *p*-values ranging from 1.0e-5 to 1.0e-20, which represents a strict criterion.

Assuming that the TFs in MM and bacteroid profiles are involved in the transcription of their corresponding protein profile, a bioinformatic study with conserved motifs of TFs was used to establish a TF gen-target relationship, and a transcriptional regulatory network for MM and bacteroid was proposed.

## Materials and methods

### Culture of *Rhizobium etli* CFN42 strain

The *R. etli* CFN42 strain was grown in minimal medium with ammonium chloride and succinic acid as previously reported (Taboada et al. 2018), it was cultured for 6 h, and the cells were pelleted by centrifugation at 7,500 × *g* at 5°C, for 5 min.

### Plant inoculation with *Rhizobium etli* CFN42

The *Phaseolus vulgaris* bean seeds were surface sterilized and placed on 0.8% agar in Petri dishes (Wacek and Brill 1976). Each seed was inoculated with 10^5^ *R. etli* cells previously washed with sterilized distilled water after growing in a peptone-yeast-rich medium as described (Encarnación et al. 1995); after 18 days post-inoculation, the bacteroid were extracted on a percoll gradient as described (Romanov et al. 1994).

### Sample preparation

The cell pellets from both free-living bacteria and bacteroid were lysed in a solution containing 7 M urea, 2% CHAPS, 1 mM DTT in 50 mM Tris–HCl pH 8. Cells were resuspended in the lysis buffer and sonicated on the ice for 15 microns. Samples were incubated with an additional 20 mM DTT for 30 min at 40°C to completely reduce disulfide bridges. Cysteine residues were alkylated with 50 mM IAA for 30 min at room temperature in darkness. After centrifugation, the proteins were collected in the supernatant. Proteins were precipitated overnight with cold ethanol (9 volumes) and washed with a 90% ethanol solution.

The precipitate was dissolved in sodium deoxycholate SDC 0.5%, SDS 0.5%, in 100 mM triethylammonium bicarbonate buffer (TEAB). Proteins were submitted to a chemical acetylation reaction of all lysine residues as previously described (Gil et al. 2017; Gil and Encarnación-Guevara 2022). Fully acetylated proteins were dissolved in AmBiC 50 mM, SDC 0.5%, and digested by adding trypsin to a ratio of 1:50 (enzyme:protein), and the reaction was incubated for 16 h at 37°C. SDC was removed with ethyl acetate acidified with trifluoroacetic acid (TFA) as previously reported (Gil et al. 2017; Gil and Encarnación-Guevara 2022). The peptide mixture was dried on a Speed-Vac and stored at −80°C until MS analysis.

### LC–MS/MS and data analysis

Peptides were dissolved in 0.1% TFA in water and loaded on an RSLC nano UPLC system (Ultimate 3000, Dionex) coupled to a Q-Exactive high-resolution mass spectrometer (Thermo Fischer Scientific). The chromatographic conditions, as well as the MS acquisition parameters, were as previously described (Gil et al. 2017). The analysis was performed at the Proteomics Core Facility, Ecole Polytechnique Fédérale de Lausanne in Switzerland. The data presented in the study are deposited in the ProteomeXchange Consortium via the PRIDE repository (Perez-Riverol et al. 2022), accession numbers PXD035204 and 10.6019/PXD035204.

Raw data were processed for peptide and protein identification/quantification using the MaxQuant platform. The database search parameters were as follows: Trypsin/R was selected as the digestion enzyme, up to two missed cleavages were allowed, carbamidomethylcysteine and acetylated lysine were set as fix modifications, and oxidized methionine was considered variable. The database used for protein identification was released in 2006 (González et al. 2006) and is publicly available through the UniProt repository. Three biological replicates of each condition were included in the study. Proteins and peptides were identified with an FDR of 1% based on the target-decoy strategy integrated in the software.

### Statistical analysis

**Figure 1:**
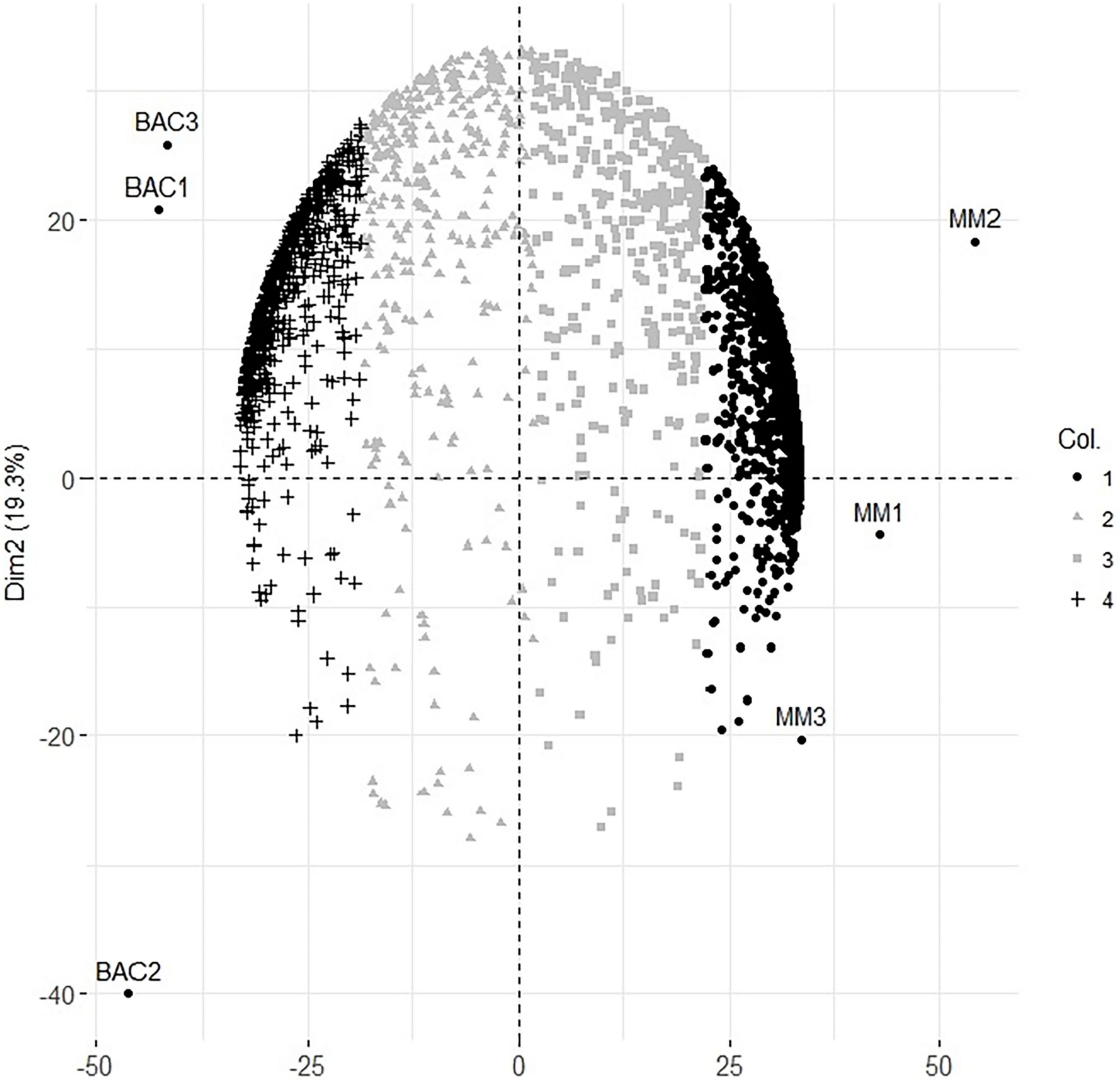
Principal component analysis of protein expression at 6 h of minimal medium growth MM1, MM2, MM3 and 18 days post inoculation Bacteroids of the symbiosis from *Rhizobium etli* CFN42 with the plant Phaseolus vulgaris Bac1, Bac2, Bac3 biological replicates.

Only proteins identified with at least two peptides and one of these unique peptides and at least two intensity values in each condition were used for statistical analysis. The protein abundance was normalized, and missing values were imputed with the Random Forest method (missForest, R package; Stekhoven and Bühlmann 2012). The PCA was carried out on the protein intensity correlation matrix (FactoMiner, R package; Lê et al. 2008) to generate a protein abundance pattern for the cell lines. To determine whether any component could distinguish between the cell lines, the sample scores for each component were plotted. After finding the component, we identified the more correlated proteins in that component with discriminatory capacity using the square cosine of the correlation matrix between the components and the proteins (Abdi and Williams 2010). It is observed in the graphic, MM and bacteroid conditions were clustered separately but grouped by condition, recovering a great diversity of data, 64.8 and 19.3% of data for one and two dimensions, respectively, giving a total of 84.1% (Figure 1). A total of 1,730 and 735 proteins were significantly identified in the minimal medium and bacteroid, respectively. In addition, 322 proteins were without change in their expression.

### Metabolic pathways analysis

**Figure 3:**
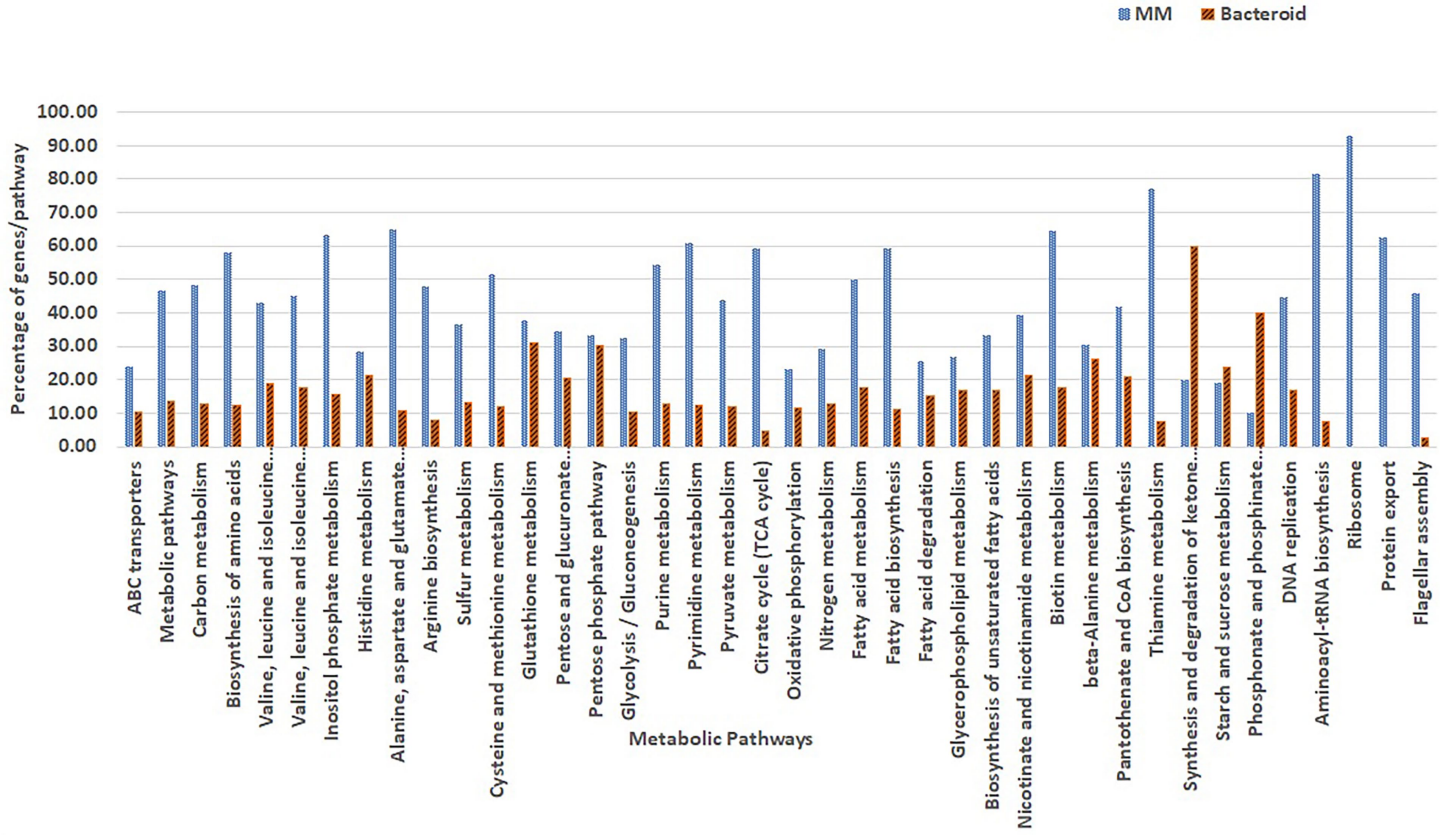
Comparison of GO classified proteins expressed per metabolic pathway in minimal medium and bacteroids from *Rhizobium etli* CFN42.

**Figure 4:**
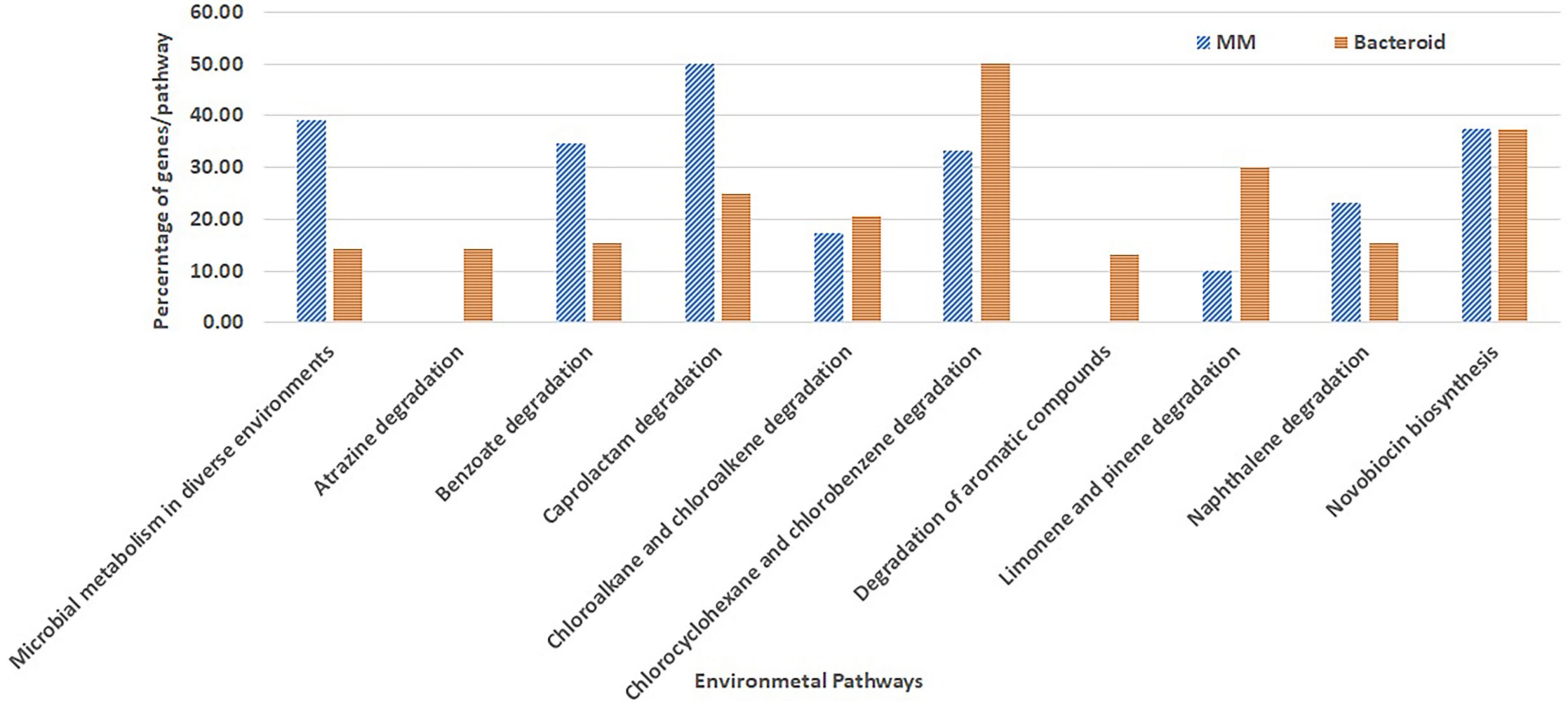
Metabolic pathways for biosynthesis and degradation of organic compounds environment related from *Rhizobium etli* CFN42.

Overrepresentation of pathways was performed online employing the Gene List Analysis tool on the PANTHER Classification System site.^2^ Only proteins with an absolute value of association with a *p*-value equal or greater than 0.5 with data of the first two components (Abdi and Williams 2010) were selected for comparative overrepresentation analysis based on Gene Ontology (Ashburner et al. 2000). To obtain the GO terms significantly overrepresented in this experiment we used the hypergeometric test and only processes with a *p*-value less than 0.05 were selected. The presence of genes for each metabolic pathway was compared as percent respect of the background number of genes per pathway in MM and bacteroid profiles (Figures 3, 4).

### Metabolic maps construction

For analysis of metabolic pathways in MM and bacteroid, and genes without changes in their expression, the Kegg mapper^3^ was used (Kanehisa 2017). This mapper uses the KO Kegg Orthology, which is based on the function of the ortholog genes. The K identifiers for *R. etli* CFN42 were obtained for the entire genome with the application blastKOALA^4^ (Kanehisa et al. 2016), the input was the sequences in FASTA format of the genes from *R. etli* CFN42 genome divided into two parts, then the *R. etli* CFN42 locus tag identifier was associated to the K identifiers, and a list including MM, bacteroid and with no change expression proteins was used in the Kegg mapper (Supplementery Table 2). Obtention of the EC number was from the KO Orthology application from Kegg^5^ (Kanehisa et al. 2016).

### Design of a regulatory network

The protein profiles of *R. etli* CFN42 grown in minimal medium (MM) at 6 h and of bacteroid isolated from nodules at 18 days post-inoculation of the bean plant *Phaseolus vulgaris*, were used to construct a transcriptional regulatory network with the application “Prediction of transcriptional regulatory networks” of the RhizoBindingSites database (Taboada-Castro et al. 2020). Briefly, this database contains predicted matrices deduced from conserved dyads (Defrance et al. 2008), composed of position-specific di or tri-nucleotides in the orthologs genes of each gene in members of the Rhizobiales taxon. These position-specific nucleotides were converted into a matrix format, which describes the conserved motifs for each gene (Hertz et al. 1990), the dyad analysis of the footprinting discovery algorithm deduced from one to five matrices per gene. The matrices of the TF’s were used to scan with a matrix-scan RSAT analysis (Nguyen et al. 2018), all the upstream regulatory sequences of the genes, establishing TF gene-target relationships data, which is in the motif information window of the RhizoBindingSites database (Taboada-Castro et al. 2020; RhizoBindingSites database user guide), this information is used in the “Prediction of a transcriptional regulatory network” application.

**Figure 5:**
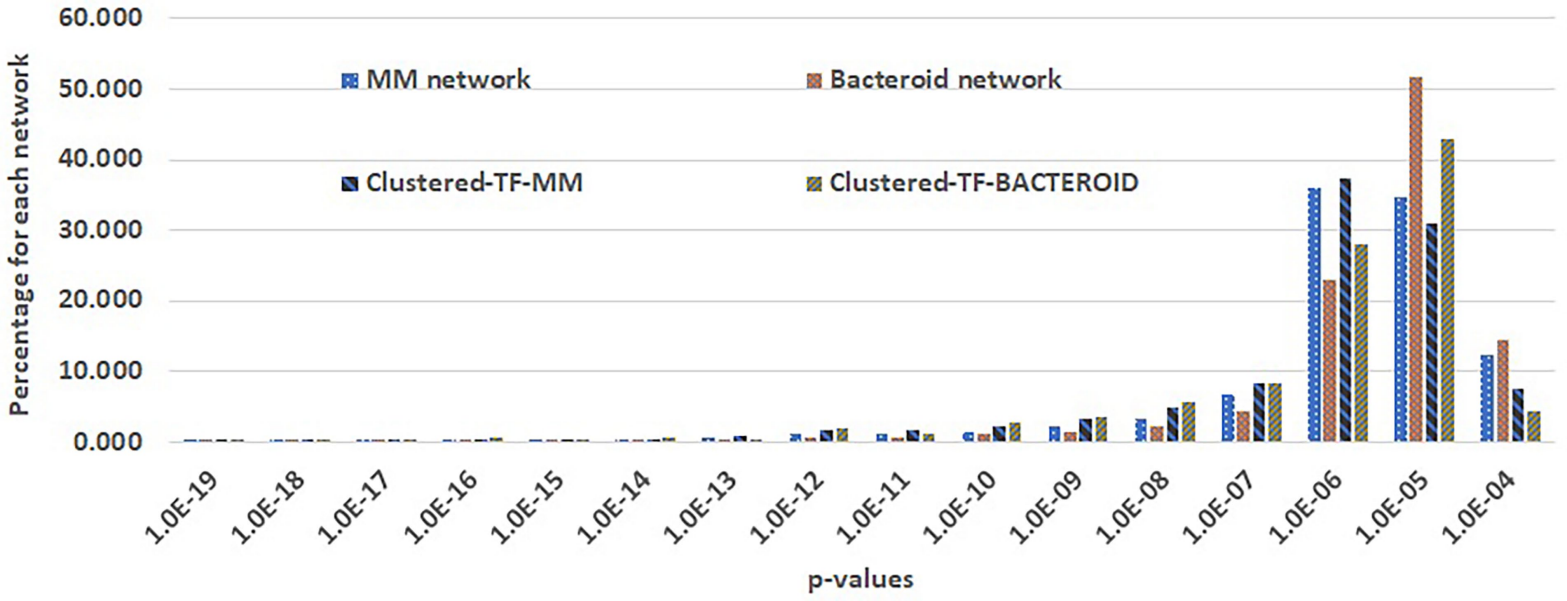
Comparison of genes per p-value range of the first and second transcriptional regulatory networks of minimal medium and bacteroid from *Rhizobium etli* CFN42.

For the prediction of transcriptional regulatory networks, a three-step method was implemented. The first step consisted in to construct networks with the MM protein profile including the 127 co-expressed TF´s. As well as, the bacteroid protein profile with the 62 co-expressed TFs, with the application “Prediction of the transcriptional regulatory network” from the RhizoBindingSites database, with the “auto” option. This step, is to eliminate the genes of the proteins not recognized by any TF, and TF’s whose matrices had no homology with any upstream regulatory sequences of potential target genes. With the option “auto”. With this option, the application searches for TF gene-target relationships for each of the TF’s co-expressed with the entered genes by looking into the motif information data from the RhizoBindingSites database. Only 1,336 genes, including 107 TF’s genes from MM (Supplementary Table 1A) and 583 genes including, 50 TF’s co-expressed bacteroid genes (Supplementary Table 1B), respectively, were found with a relationship, giving rise to hypothetical regulons available (Supplementary Tables 1A, B). The TF-matrices may have homology with the upstream regulatory sequences of target genes three levels of stringency, low stringency (*p*-value from 1.0e-4 to 9.9e-4), medium stringency level (*p*-value 1.0e-5 to 9.9e-5) or highly strict (from the *p*-value 1.0e-6 to lower *p*-values). In the second step, a matrix- clustering analysis for each condition, with the matrices of the 1336 genes of MM, as well as, the matrices of the 583 bacteroid genes including their respective TF´s was done (Castro-Mondragon et al. 2017). This step is to eliminate false-positive data as possibly, since the motifs are short conserved functionally compromised sequences (Ihuegbu et al. 2012), to avoid possible TF gene-target relationships by chance. In this analysis, the matrix of a TF should be grouped by homology with the nucleotide sequence of matrices of the potential target genes. Matrix-clustering algorithm creates the file clusters_motif_names.tab, which is edited to obtain all the genes whose matrices were clustered containing at least two different genes. Only the clusters, including matrices of a TF or TFs (Clustered-TF) were selected from MM (Supplementary Table 1C) and bacteroid profiles (Supplementary Table 1D), the NCBI genomic information of the genes was added to these tables as well as the Clustered-TF for each cluster (column headed “Clustered-TF” Supplementary Tables 1C, D). An alignment of MM and bacteroid matrices from matrix-clustering showed how much conserved are motifs in the clusters (Supplementary Tables 1E, F, respectively). The matrices were grouped into 207 and 92 clusters for MM and bacteroid, respectively. In this second step, additional depuration of genes after a matrix-clustering analysis was observed since only 655 genes, including 93 TF’s genes from MM, and 247 genes, including 46 TF’s genes, were clustered. A TF gen-target relationship with only genes of a clusters was confirmed (Results and discussion, Appendix G and H). In the third step, second networks were constructed (as in the first step) only with clustered-TF genes, called “Clustered-TF-MM” and “Clustered-TF-BACTEROID” (Figure 5). and cluster_97 and cluster_112 from bacteroid were chosen. For cluster_34, all the genes had a TF gene-target relationship. For cluster_195, 22 out of 27 genes were connected (Supplementary Table 1G). For cluster_97, 21 of 26 genes were connected and for cluster_112, 21 from 22 genes were connected (Supplementary Table 1H). It is worth noticing that, after the matrix-clustering grouping genes, all the genes for each condition had one or more relationships. Quality of MM, bacteroid, clustered-TF-MM and Clustered-TF-BACTEROID networks were analyzed (Results and discussion, Figure 5). Then, the transcriptional regulatory networks of MM and bacteroid protein profiles are constructed with motifs interspecies conserved.

These data confirmed that clustered matrices of genes are strongly related to the structure of a network, and these genes probably represent hubs.

To search for expected transcriptional regulation for isoenzymes in MM and bacteroids, the tables of the transcriptional regulatory networks described above were ordered in decreasing order by the column headed “*p*-value” (Supplementary Tables 1A, B). These tables were identified in the right column with the condition they pertain giving rise to new files ordered from MM and bacteroid and Clustered-TF-MM and Clustered-TF-BACTEROID separately. A Supplementary Table 1I containing the “K” number with a *R. etli* CFN42 locus tag identifier and the pertaining physiological condition per row was constructed. Then, the table from Supplementary Table 1I was paired with new files from the MM and bacteroid and Clustered-TF-MM and Clustered-TF-BACTEROID aforementioned. A new file with three groups of columns was produced; the first group contains information on the expected regulation with information from MM and bacteroid networks (with columns; Condition, Locus tag, K number, Upstream_region, Matrix_ID, Chain, End_motif, Start_motif, Site, Weight, *p*-value, and Significance). The second group of columns contains information of the expected transcriptional regulation with information from the Clustered-TF-MM and Clustered-TF-BACTEROID with columns headed as the MM and bacteroid data. The third group of columns contains information of the enzymatic function of the K numbers headed as; Condition, Locus tag, K number, Compartment, locus name, COG number, COG group, Function from KO orthology, and Function from NBCI. To look for the expected transcriptional regulation for the same K number with different locus tag in MM and bacteroid, it was located in the column “Matrix_ID” with a format “RHE_RS13345_m5,” which means the TF is RHE_RS13345 and “_m5” means the matrix number “5” of the TF (as was mentioned, a TF has one to five matrices; Supplementary Table 4).

### Properties of networks

The most recent *E. coli* and *B. subtilis* “strong” evidence networks were retrieved from Abasy Atlas v2.2 (Escorcia-Rodríguez et al. 2020). Both networks only include regulatory interactions supported by experiments showing a direct interaction between the transcription factor and the upstream region of the target gene. We contrasted the inferred networks with the *E. coli* and *B. subtilis* curated networks as a positive control, and 1000 Erdös-Rényi random networks parametrized having the same number of nodes and edges as the corresponding biological networks as a negative control.

We computed several global structural properties for regulatory networks. Namely, regulators ( $k_{\mathrm{out}}>0$ ), self-regulations, maximum out-connectivity, giant component size, network density, feedforward circuits, complex feedforward circuits, 3-Feedback loops, average shortest path length, network diameter, average clustering coefficient, adjusted coefficient of determination ( $R_{adj}^{2}$ ) of $P\left(k\right)$ , and $R_{adj}^{2}$ of $C\left(k\right)$ . Regulators, self-regulations, maximum out-connectivity, and giant component size were normalized by the number of nodes in the network. The density was included as the product of the network density and the fraction of regulators. Network diameter was normalized by (number of nodes – 2; as if no shortcuts would exist). 3-feedback loops, feedforward loops, and complex feedforward loops were normalized by the number of potential motifs in the network, defined as:


$$\frac{n!}{\left(n-r\right)!}\cdot {\left(\frac{TF_{n}}{n}\right)}^{TF_{m}}$$

Where n is the number of nodes in the network, r is the number of nodes in the motif ( r=3 ), $TF_{n}$ is the number of TFs in the network, and $TF_{m}$ is the number of TFs required for each motif type ( $TF_{m}=3$ for 3-feedback loops, and $TF_{m}=2$ for feedforward and complex feedforward loops). We scaled the values of each property vector across networks to the range between 0 and 1, inclusively. Then, we clustered networks and properties using Ward’s method. Further, we used pairwise Pearson correlation for the network property profiles and clustered the networks according to the Euclidean distance using Ward’s method.

### Hierarchy reconstruction of networks

First, we removed all the structural genes (nodes having $k_{\mathrm{out}}=0$ ) and their interactions from the network. Next, we classified each network edge (a, b) as ‘top-down’ if $k_{a}^{\mathrm{out}}>k_{b}^{\mathrm{out}}$ (where $k_{n}^{\mathrm{out}}$ is the out-connectivity of node *n*), otherwise, it was classified as ‘bottom-up’. Then, we removed all the ‘bottom-up’ edges from the network. This step removed the feedback circuits present in the network, transforming it into a directed acyclic graph. Then we applied a modified topological sorting algorithm that returned the list of layers composing the hierarchy, where each node in a layer only can regulate nodes in lower layers. As the number of ‘bottom-up’ edges is low (<5% in average), our strategy maintains the global structure of the network to reveal the hierarchy. Besides structural nodes, no other nodes are removed, and ‘bottom-up’ edges can be added back to the hierarchy to reveal the feedback among layers and reconstruct the original network.

## Results and discussion

In a previous study, we identified 292 proteins of the symbiotic state at 18 days post-inoculation (Resendis-Antonio et al. 2011); now, we discuss new data covering 2.5 times more proteins from symbiosis in this work. Principal components (one and two) covered 84.1% of the total initial data (Figure 1). The update of the *R. etli* CFN42-*Phaseolus vulgaris* bean plant symbiosis is with 1,730, 738, and 323 protein profiles for MM, bacteroid, and without no change in their expression, respectively (see below). There were 39.7% of common proteins in the bacteroid between the previous report (Resendis-Antonio et al. 2011) and this study. The low coverage observed in the new data may be due to the great diversity of different experiments collected for the last study. While in the new data, the variation in the experimental condition was from only two biological replicates, because our interest was to take advantage of the TF and non-TF protein co-expression (Galán-Vásquez and Perez-Rueda 2019), under the assumption that these TFs were involved in the transcriptional regulation of these proteins, to establish a TF gene–target relationship, only new data are considered in this analysis, and our previous data are considered only for discussion.

### Compartmentation of proteins in MM and bacteroid

**Figure 2:**
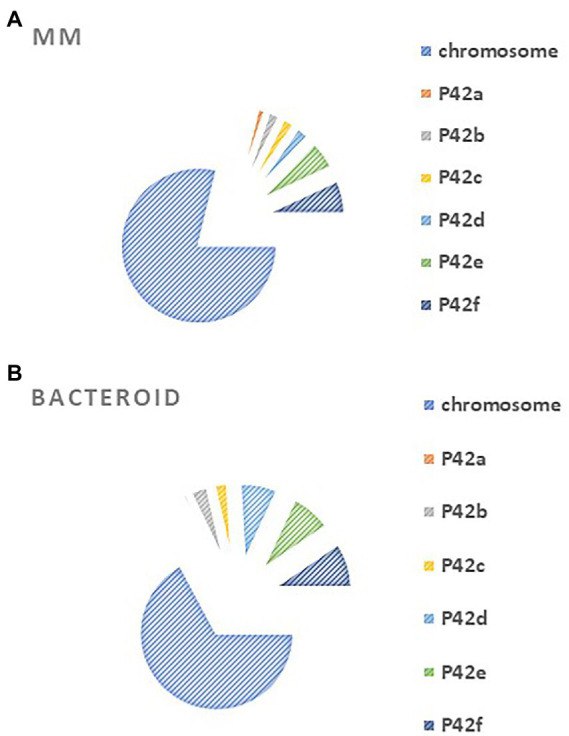
Compartmentation of **(A)** minimal medium protein profile expressed at six hours of growth in minimal medium and **(B)** 18 days post inoculation of bacteroid protein profile from *Rhizobium etli* CFN42.

*Rhizobium etli* CFN42 contains six plasmids and a chromosome (González et al. 2003). An analysis of gene location from MM and bacteroid showed that for MM proteins, most of the genes are codified in the chromosome, while for bacteroid proteins, higher participation was found for plasmids p42b, p42d, p42e, p42f than in MM (Figure 2). Of note, the symbiotic plasmid (p42d) had a 5.3% higher participation in bacteroids than in MM, in line with a wide transcription rate of the symbiotic plasmid (psym) genes of *R. etli* CFN42 under microaerobic conditions (as in symbiosis) or in aerobic conditions in the presence of genistein (Valderrama et al. 1996). Additionally, many of the genes expressed in MM (78.7%) and bacteroid (67.1%) were from the chromosome, while 21 and 32% of the expressed genes were from plasmids, respectively. The higher number of genes expressed from the chromosome agrees with the finding that exponential growth in MM and nitrogen fixation activity have in common a great demand for energy synthesis, and most of the metabolic pathways for this process are similar (see below). One of the exceptional differences is that in symbiosis, the high-affinity *cbb3* cytochrome oxidase terminal is expressed (Delgado et al. 1998; Lopez et al. 2001). *Rhizobium leguminosarum* bv. *viciae* UMP791 contain five plasmids and a chromosome, similar to *R. etli* CFN42, which contains six plasmids. A proteome analysis of *R. leguminosarum* bacteroid with its host plant *Pisum sativum* showed that most of the bacteroid proteins were from the chromosome (81.6%), showing a lower participation of the plasmids than with the *R. etli* CFN42 strain (Durán et al. 2020).

The plasmids contain essential genes for growth in MM, such as p42e (*minCDE*; Landeta et al. 2011) and plasmid p42f (*panCB*; Villaseñor et al. 2011). Moreover, a cured *R. etli* CFN42 of p42f complemented with the *panCB* genes did not restore wild-type growth, meaning that p42f has unidentified genes that are important for growth in MM (Villaseñor et al. 2011).

### Metabolic pathways

A detailed view of the pathways that operate in exponential growth in MM (ammonium-succinate) and bacteroid, a non-growing state in symbiosis at 18 days post-inoculation, with a maximal peak of nitrogen fixation, showed 105 pathways according to the KEEG program with Gen Ontology (GO) gene off classification (see “Materials and Methods” section; Maere et al. 2005; Figure 3). In MM, 31 representative metabolic pathways with greater or equal to 30% of genes, and in bacteroid, 10 pathways with greater or equal to 20% of genes per pathway with respect to the genome content were found (Figure 3), which is related to the high demand for the synthesis of metabolites to sustain growth in a minimal medium compared to the non-growing bacteroid state. In contrast, in symbiosis, most of the energy for the synthesis of metabolites is dedicated to nitrogen fixation. In agreement with this, carbon metabolism, including synthesis of amino acids, sugars, purine and pyrimidine, sulfur metabolism, glycolysis-gluconeogenesis, pyruvate metabolism, TCA cycle, oxidative phosphorylation, nitrogen metabolism, fatty acid metabolism, nicotinate and nicotinamide, vitamin synthesis, DNA replication, aminoacyl-tRNA biosynthesis, ribosome synthesis, protein export, and flagellar assembly, had a higher percentage of genes in MM than in bacteroids (Resendis-Antonio et al. 2011), as was shown in a comparative proteomic study of a free-living aerobic condition and the symbiosis of the *Bradyrhizobium japonicum* USDA110 strain (Sarma and Emerich 2005, 2006). Some other pathways, such as histidine metabolism, glutathione metabolism, pentose phosphate pathway, beta-alanine, starch, and sucrose metabolism, were similar in MM and bacteroids; likely, histidine metabolism is necessary for the synthesis of inosine monophosphate, a precursor for the synthesis of purines and subsequently for the synthesis of allantoin and allantoic acids. These nitrogen compounds from nitrogen fixation are exported to the bean plant *Phaseolus vulgaris* by the *R. etli* CFN42 bacteroid (Alamillo et al. 2010; Collier and Tegeder 2012). Glutathione plays a crucial role against oxidative damage during the establishment of symbiosis (Hérouart et al. 2002); it is a precursor for cysteine synthesis, a sulfur donor for the synthesis of the Fe-S centers involved in defense against oxidative stress and in the prosthetic groups of sensory proteins. The pentose phosphate pathway is essential for the synthesis of phosphoribosyl pyrophosphate (PRPP), a precursor for purine synthesis during symbiosis (Newman et al. 1994; Miranda-Ríos et al. 1997). Beta-alanine is a precursor for the synthesis of pantothenate, which is essential for the ubiquitous compound coenzyme A (coA), subsequently used for many metabolic reactions, including phospholipid synthesis, fatty acid synthesis and degradation, and the tricarboxylic acid cycle. The *panCB* genes for the synthesis of pantothenate codified in p42f from the *R. etli* CFN42 strain were characterized (Villaseñor et al. 2011). Starch and sucrose synthesis was not detected in free-living or symbiotic conditions of *R. etli* CFN42. For the synthesis and degradation of ketone bodies, phosphonate and phosphinate metabolism were higher in bacteroids than in MM. *R. etli* CFN42 synthesizes poly-β-hydroxybutyrate granules during symbiosis with *P. vulgaris*; because this polymer is a reserve of carbon and reducing power, its accumulation is greater in symbiosis than in MM, where the energy is for supporting growth (Cevallos et al. 1996). Additionally, there is a high demand for phosphate in nitrogen-fixing nodules; it is an essential macronutrient necessary for the synthesis of proteins and nucleic acids (Liu et al. 2018), and phosphate is probably limited during symbiosis. As a response, the transcription of this pathway is raised, as was shown for bacteroids harvested from soybeans grown under field conditions (Delmotte et al. 2010). A detailed study with transcriptomic and proteomic technologies of the symbiosis compared with the aerobic growth showed 3,587 genes/proteins, expressing 43% of the predicted genome from *B. japonicum* (Delmotte et al. 2010), 807 proteins were identified in symbiosis; while in this study, 738 proteins were identified; i.e., in this study, there is a great proteomic coverage of the symbiosis *R. etli* CFN42-*Phaseolus vulgaris* bean plant considering that the *B japonicum* genome size is bigger than the *R. etli* genome. Although *R. etli* is a fast grower and *B. japonicum* is a slow grower in minimal medium, they elicit determinate nodules. In contrast to the symbiont *S. meliloti* with their host *Medicago sativa* alfalfa plant that induces indeterminate nodules, there are notable differences between the structure and composition of the symbiont in determinate and indeterminate nodules, reviewed in (Rascio and La Rocca 2013). Although *B. japonicum* and *R. etli* symbiosis occur in temperate and tropical weather, respectively, despite these differences, *R. etli* and *B. japonicum* symbiosis is more similar than *S. meliloti* symbiosis. A proteomic comparison of free-living and symbiosis from *B. japonicum* showed a greater number of proteases in free life than in symbiosis (Sarma and Emerich 2006). Similarly, in this study, 27 and two proteases were expressed. Most likely, the recycling of metabolites may be one of the factors that impacts the spending of energy in free life and symbiosis. It was suggested that bacteroids expend their energy judiciously between protein synthesis and nitrogen fixation by altering protein turnover (Sarma and Emerich 2006).

### Environmental metabolism

Moreover, some GO genes classified for the metabolism of environmental compounds were mapped; in MM, these genes covered approximately 39% of genes, while in bacteroids, they covered 14% with respect to the genome content (microbial metabolism in diverse environments, Figure 4). For some pathways, there is a low representation with respect to the total content of the *R. etli* CFN42 genome. The pathways for the degradation of benzoate, caprolactam, and naphthalene were more highly expressed in MM than in bacteroids. For chloroalkane and chloroalkene degradation and novobiocin biosynthesis, the number of proteins expressed was similar (Figure 4). Proteins for the degradation of chloroalkane and chloroalkene were also identified in a metagenomic analysis in the rhizosphere soil of a constructed wetland (Bai et al. 2014). Novobiocin is a very potent inhibitor of DNA gyrase, which works by targeting the GyrB subunit of the enzyme for energy transduction, and resistance to novobiocin of *Lotus rhizobia* was related to the effectiveness of the symbiosis with *Lotus pedunculatus* (Pankhurst 1977). For the degradation of atrazine, chlorocyclohexane and chlorobenzene, and aromatic compounds limonene and pinene, the number of genes was higher in bacteroid than in MM. Atrazine is an herbicide that may inhibit the growth of *Rhizobium* species, *P. vulgaris*-*Rhizobium* sp. Consortium symbiosis has been used for the bioremediation of soil contaminated with atrazine (Madariaga-Navarrete et al. 2017). Genes for the degradation of the aromatic compounds chlorocyclohexane and chlorobenzene were also reported in the genome of *Burkholderia phenoliruptrix* BR3459a, a symbiont of the *Mimosa flocculosa* leguminous plant (Zuleta et al. 2014). It was observed that for the *Rhizobium leguminosarum* E20-8 strain, limonene and pinene have antioxidant activity promoting growth under stress provoked by cadmium (Sá et al. 2020) and antibacterial activity (Ghaffari et al. 2019). In the *B. japonicum* bacteroid proteome, the NrgC protein and a gene for phenazine biosynthesis were identified for a response against microbial attack (Sarma and Emerich 2005, 2006). These genes in MM and bacteroid for degradation of metabolites of the environment are used for a fast response, competence, and better adaptation in soil conditions. Unlike *B. japonicum* (Sarma and Emerich 2006), *R. etli* bacteroid showed a wide strategy to withstand environmental stresses.

### Isoenzymes in MM and bacteroid

The KEEG mapper for visualization of the metabolic maps was used (see “Materials and Methods” section). This mapper uses the “K” number to identify the function of the gene, and it is assigned based on the orthology of the genes (Kanehisa et al. 2016). For an integral view of the metabolism in MM, bacteroid, and proteins present in both conditions with “no change” (Nch), genes were mapped (Supplementary Tables 2 and 3A). Discussion of the central metabolism involved 37 representative pathways. Analysis of mapped genes showed that for some enzymatic reactions, different genes for the same enzymatic step in MM and bacteroid were found, e.g., for the pentose phosphate pathway there were two genes for the conversion of D-ribulose phosphate to D-ribose-5P by the 6-phosphogluconate dehydrogenase enzyme; one is expressed in MM RHE_RS12615, and a different one was expressed in the bacteroid RHE_RS17825 (Table 1), suggesting the presence of a condition-dependent isoform (Supplementary Table 2 pathway 15, and Supplementary Table 3A). From here on, we will call it “multiplicity.” The Fructose and mannose metabolism pathway (Supplementary Table 2 pathway 10, and Supplementary Table 3A), for the catalysis of L-fucose to L-fucolactone by the enzyme D-threo-aldose 1-dehydrogenase; the proteins in MM RHE_RS02500 and bacteroid RHE_RS28605 were expressed (Table 1), showing multiplicity. For the galactose metabolism pathway (Supplementary Table 2 pathway 11, and Supplementary Table 3A), the conversion of UDP-glucose to UDP-galactose, UDP-glucose 4-epimerase was synthesized in MM RHE_RS03845 and in bacteroid RHE_RS17845 was expressed. As well as, for the enzyme *dgoD*, galactonate dehydratase [EC:4.2.1.6] for catalysis of D-galactonate to 2-dehydro-3-deoxy-D-galactonate in MM the RHE_RS18905 and in bacteroid RHE_RS24515 proteins were expressed (Table 1), showing multiplicity for two different enzymes of the same pathway. These data showed that the same enzymatic reactions are performed in MM and bacteroids with distinct proteins, suggesting that some alternative proteins are specific for free-living aerobic conditions and others for symbiosis for the same metabolic step. The pyruvate metabolism pathway (Supplementary Table 2 pathway 18, and Supplementary Table 3A), for the conversion of acetyl-CoA to acetoacetyl-CoA in MM, RHE_RS23190 was expressed, while in bacteroid, two different genes were expressed; RHE_RS02820 and RHE_RS20545 which showed differences in metabolism from MM and in bacteroid (Table 1), and since distinct TFs were identified in MM and bacteroid, a different transcriptional regulation for isoenzymes was analyzed (see below). Moreover, for the inositol phosphate pathway (Supplementary Table 2 pathway 2, and Supplementary Table 3A), for the myo-inositol-1(or 4)-monophosphatase enzyme in MM was identified the RHE_RS10865, RHE_RS17960, and RHE_RS22570 enzymes, and in bacteroid RHE_RS22680 and RHE_RS04240 were found (Table 1), again showing that the metabolism in symbiosis compared with MM has some differences. For valine, leucine, and isoleucine biosynthesis pathway (Supplementary Table 2 pathway 6, and Supplementary Table 3A), the enzyme *ilvD*, dihydroxy-acid dehydratase [EC:4.2.1.9], and the RHE_RS08720 and RHE_RS23070 proteins were expressed in MM and bacteroid, respectively, supporting multiplicity (Table 1). Similarly, for valine, leucine, and isoleucine degradation (Supplementary Table 2 pathway 7, and Supplementary Table 3A), the enzyme *acd* acyl-CoA dehydrogenase [EC:1.3.8.7] RHE_RS20670 was expressed in MM and in bacteroid, the isoenzyme RHE_RS04555 was identified (Table 1). Additionally, for the enzyme *atoB*, acetyl-CoA C-acetyltransferase [EC:2.3.1.9] in MM the RHE_RS23190 was present, and in bacteroids, the isoenzymes RHE_RS02820 and RHE_RS20545 were found (Table 1), showing a multigenic strategy for the degradation of branched-chain amino acids. For the synthesis of the poly-β-hydroxybutyrate polymer, the enzyme β-ketothiolase (acetyl-CoA C-acetyltransferase) converts two molecules of acetyl-CoA to acetoacetyl-CoA. In MM, the enzyme RHE_RS23190 was detected, and in bacteroid, two enzymes, RHE_RS02820 and RHE_RS20545, were identified (Table 1).

**Table 1:** Isoenzymes in MM and bacteroid from *Rhizobium etli* CFN42.

**K number**	**Physiological condition**	**Locus tag**	Annotation from BlastKoala*
K02035	MM	RHE_RS10550	ABC.PE.S; peptide/nickel transport system substrate-binding protein
K02035	MM	RHE_RS20405	ABC.PE.S; peptide/nickel transport system substrate-binding protein
K02035	MM	RHE_RS22160	ABC.PE.S; peptide/nickel transport system substrate-binding protein
K02035	MM	RHE_RS23500	ABC.PE.S; peptide/nickel transport system substrate-binding protein
K02035	MM	RHE_RS23525	ABC.PE.S; peptide/nickel transport system substrate-binding protein
K02035	MM	RHE_RS24485	ABC.PE.S; peptide/nickel transport system substrate-binding protein
K02035	MM	RHE_RS27640	ABC.PE.S; peptide/nickel transport system substrate-binding protein
K02035	MM	RHE_RS27665	ABC.PE.S; peptide/nickel transport system substrate-binding protein
K02035	MM	RHE_RS03080	ABC.PE.S; peptide/nickel transport system substrate-binding protein
K02035	Bacteroid	RHE_RS10750	ABC.PE.S; peptide/nickel transport system substrate-binding protein
K02035	Bacteroid	RHE_RS22645	ABC.PE.S; peptide/nickel transport system substrate-binding protein
K02035	Bacteroid	RHE_RS28255	ABC.PE.S; peptide/nickel transport system substrate-binding protein
K02035	Bacteroid	RHE_RS01120	ABC.PE.S; peptide/nickel transport system substrate-binding protein
K02052	MM	RHE_RS17470	ABC.SP.A; putative spermidine/putrescine transport system ATP-binding protein
K02052	Bacteroid	RHE_RS14790	ABC.SP.A; putative spermidine/putrescine transport system ATP-binding protein
K02052	Bacteroid	RHE_RS14870	ABC.SP.A; putative spermidine/putrescine transport system ATP-binding protein
K00249	MM	RHE_RS20670	ACADM, acd; acyl-CoA dehydrogenase [EC:1.3.8.7]
K00249	Bacteroid	RHE_RS04555	ACADM, acd; acyl-CoA dehydrogenase [EC:1.3.8.7]
K00626	MM	RHE_RS23190	ACAT, atoB; acetyl-CoA C-acetyltransferase [EC:2.3.1.9]
K00626	Bacteroid	RHE_RS20545	ACAT, atoB; acetyl-CoA C-acetyltransferase [EC:2.3.1.9]
K00626	Bacteroid	RHE_RS02820	ACAT, atoB; acetyl-CoA C-acetyltransferase [EC:2.3.1.9]
K01486	MM	RHE_RS17480	ade; adenine deaminase [EC:3.5.4.2]
K01486	Bacteroid	RHE_RS15825	ade; adenine deaminase [EC:3.5.4.2]
K02012	MM	RHE_RS10880	afuA, fbpA; iron(III) transport system substrate-binding protein
K02012	Bacteroid	RHE_RS13955	afuA, fbpA; iron(III) transport system substrate-binding protein
K00759	MM	RHE_RS15525	APRT, apt; adenine phosphoribosyltransferase [EC:2.4.2.7]
K00759	Bacteroid	RHE_RS31115	APRT, apt; adenine phosphoribosyltransferase [EC:2.4.2.7]
K05349	MM	RHE_RS28885	bglX; beta-glucosidase [EC:3.2.1.21]
K05349	Bacteroid	RHE_RS29645	bglX; beta-glucosidase [EC:3.2.1.21]
K01255	MM	RHE_RS01080	CARP, pepA; leucyl aminopeptidase [EC:3.4.11.1]
K01255	Bacteroid	RHE_RS07430	CARP, pepA; leucyl aminopeptidase [EC:3.4.11.1]
K00405	MM	RHE_RS29065	ccoO; cytochrome c oxidase cbb3-type subunit II
K00405	Bacteroid	RHE_RS30885	ccoO; cytochrome c oxidase cbb3-type subunit II
K03412	MM	RHE_RS03250	cheB; two-component system, chemotaxis family, protein-glutamate methylesterase/glutaminase [EC:3.1.1.61 3.5.1.44]
K03412	Bacteroid	RHE_RS26805	cheB; two-component system, chemotaxis family, protein-glutamate methylesterase/glutaminase [EC:3.1.1.61 3.5.1.44]
K03412	Bacteroid	RHE_RS17965	cheB; two-component system, chemotaxis family, protein-glutamate methylesterase/glutaminase [EC:3.1.1.61 3.5.1.44]
K00390	MM	RHE_RS05785	cysH; phosphoadenosine phosphosulfate reductase [EC:1.8.4.8 1.8.4.10]
K00390	Bacteroid	RHE_RS05785	cysH; phosphoadenosine phosphosulfate reductase [EC:1.8.4.8 1.8.4.10]
K00285	MM	RHE_RS28700	dadA; D-amino-acid dehydrogenase [EC:1.4.5.1]
K00285	Bacteroid	RHE_RS03755	dadA; D-amino-acid dehydrogenase [EC:1.4.5.1]
K01714	MM	RHE_RS26910	dapA; 4-hydroxy-tetrahydrodipicolinate synthase [EC:4.3.3.7]
K01714	MM	RHE_RS27660	dapA; 4-hydroxy-tetrahydrodipicolinate synthase [EC:4.3.3.7]
K01714	MM	RHE_RS03065	dapA; 4-hydroxy-tetrahydrodipicolinate synthase [EC:4.3.3.7]
K01714	Bacteroid	RHE_RS07055	dapA; 4-hydroxy-tetrahydrodipicolinate synthase [EC:4.3.3.7]
K01714	Bacteroid	RHE_RS19830	dapA; 4-hydroxy-tetrahydrodipicolinate synthase [EC:4.3.3.7]
K01714	Bacteroid	RHE_RS22155	dapA; 4-hydroxy-tetrahydrodipicolinate synthase [EC:4.3.3.7]
K01714	Bacteroid	RHE_RS14280	dapA; 4-hydroxy-tetrahydrodipicolinate synthase [EC:4.3.3.7]
K02031	MM	RHE_RS24230	ddpD; peptide/nickel transport system ATP-binding protein
K02031	MM	RHE_RS24500	ddpD; peptide/nickel transport system ATP-binding protein
K02031	MM	RHE_RS25825	ddpD; peptide/nickel transport system ATP-binding protein
K02031	MM	RHE_RS27625	ddpD; peptide/nickel transport system ATP-binding protein
K02031	MM	RHE_RS20420	ddpD; peptide/nickel transport system ATP-binding protein
K02031	Bacteroid	RHE_RS28270	ddpD; peptide/nickel transport system ATP-binding protein
K01684	MM	RHE_RS18905	dgoD; galactonate dehydratase [EC:4.2.1.6]
K01684	Bacteroid	RHE_RS24515	dgoD; galactonate dehydratase [EC:4.2.1.6]
K00064	MM	RHE_RS02500	E1.1.1.122; D-threo-aldose 1-dehydrogenase [EC:1.1.1.122]
K00064	Bacteroid	RHE_RS28605	E1.1.1.122; D-threo-aldose 1-dehydrogenase [EC:1.1.1.122]
K01092	MM	RHE_RS17960	E3.1.3.25, IMPA, suhB; myo-inositol-1(or 4)-monophosphatase [EC:3.1.3.25]
K01092	MM	RHE_RS22570	E3.1.3.25, IMPA, suhB; myo-inositol-1(or 4)-monophosphatase [EC:3.1.3.25]
K01092	MM	RHE_RS10865	E3.1.3.25, IMPA, suhB; myo-inositol-1(or 4)-monophosphatase [EC:3.1.3.25]
K01092	Bacteroid	RHE_RS04240	E3.1.3.25, IMPA, suhB; myo-inositol-1(or 4)-monophosphatase [EC:3.1.3.25]
K01092	Bacteroid	RHE_RS22680	E3.1.3.25, IMPA, suhB; myo-inositol-1(or 4)-monophosphatase [EC:3.1.3.25]
K01560	MM	RHE_RS05045	E3.8.1.2; 2-haloacid dehalogenase [EC:3.8.1.2]
K01560	Bacteroid	RHE_RS28210	E3.8.1.2; 2-haloacid dehalogenase [EC:3.8.1.2]
K01768	MM	RHE_RS18990	E4.6.1.1; adenylate cyclase [EC:4.6.1.1]
K01768	MM	RHE_RS24270	E4.6.1.1; adenylate cyclase [EC:4.6.1.1]
K01768	MM	RHE_RS18920	E4.6.1.1; adenylate cyclase [EC:4.6.1.1]
K01768	Bacteroid	RHE_RS11150	E4.6.1.1; adenylate cyclase [EC:4.6.1.1]
K01768	Bacteroid	RHE_RS12750	E4.6.1.1; adenylate cyclase [EC:4.6.1.1]
K01768	Bacteroid	RHE_RS13090	E4.6.1.1; adenylate cyclase [EC:4.6.1.1]
K01768	Bacteroid	RHE_RS13735	E4.6.1.1; adenylate cyclase [EC:4.6.1.1]
K01768	Bacteroid	RHE_RS14395	E4.6.1.1; adenylate cyclase [EC:4.6.1.1]
K01768	Bacteroid	RHE_RS18920	E4.6.1.1; adenylate cyclase [EC:4.6.1.1]
K01768	Bacteroid	RHE_RS24935	E4.6.1.1; adenylate cyclase [EC:4.6.1.1]
K09458	MM	RHE_RS12650	fabF, OXSM, CEM1; 3-oxoacyl-[acyl-carrier-protein] synthase II [EC:2.3.1.179]
K09458	MM	RHE_RS12655	fabF, OXSM, CEM1; 3-oxoacyl-[acyl-carrier-protein] synthase II [EC:2.3.1.179]
K09458	MM	RHE_RS07375	fabF, OXSM, CEM1; 3-oxoacyl-[acyl-carrier-protein] synthase II [EC:2.3.1.179]
K09458	Bacteroid	RHE_RS10850	fabF, OXSM, CEM1; 3-oxoacyl-[acyl-carrier-protein] synthase II [EC:2.3.1.179]
K00059	MM	RHE_RS06685	fabG, OAR1; 3-oxoacyl-[acyl-carrier protein] reductase [EC:1.1.1.100]
K00059	MM	RHE_RS07365	fabG, OAR1; 3-oxoacyl-[acyl-carrier protein] reductase [EC:1.1.1.100]
K00059	MM	RHE_RS05335	fabG, OAR1; 3-oxoacyl-[acyl-carrier protein] reductase [EC:1.1.1.100]
K00059	Bacteroid	RHE_RS25095	fabG, OAR1; 3-oxoacyl-[acyl-carrier protein] reductase [EC:1.1.1.100]
K00059	Bacteroid	RHE_RS19755	fabG, OAR1; 3-oxoacyl-[acyl-carrier protein] reductase [EC:1.1.1.100]
K00135	MM	RHE_RS00470	gabD; succinate-semialdehyde dehydrogenase / glutarate-semialdehyde dehydrogenase [EC:1.2.1.16 1.2.1.79 1.2.1.20]
K00135	Bacteroid	RHE_RS28200	gabD; succinate-semialdehyde dehydrogenase / glutarate-semialdehyde dehydrogenase [EC:1.2.1.16 1.2.1.79 1.2.1.20]
K00135	Bacteroid	RHE_RS29885	gabD; succinate-semialdehyde dehydrogenase / glutarate-semialdehyde dehydrogenase [EC:1.2.1.16 1.2.1.79 1.2.1.20]
K02433	MM	RHE_RS09475	gatA, QRSL1; aspartyl-tRNA(Asn)/glutamyl-tRNA(Gln) amidotransferase subunit A [EC:6.3.5.6 6.3.5.7]
K02433	Bacteroid	RHE_RS25710	gatA, QRSL1; aspartyl-tRNA(Asn)/glutamyl-tRNA(Gln) amidotransferase subunit A [EC:6.3.5.6 6.3.5.7]
K02433	Bacteroid	RHE_RS01105	gatA, QRSL1; aspartyl-tRNA(Asn)/glutamyl-tRNA(Gln) amidotransferase subunit A [EC:6.3.5.6 6.3.5.7]
K00605	MM	RHE_RS11460	gcvT, AMT; aminomethyltransferase [EC:2.1.2.10]
K00605	Bacteroid	RHE_RS26150	gcvT, AMT; aminomethyltransferase [EC:2.1.2.10]
K00605	Bacteroid	RHE_RS26195	gcvT, AMT; aminomethyltransferase [EC:2.1.2.10]
K16147	MM	RHE_RS27870	glgE; starch synthase (maltosyl-transferring) [EC:2.4.99.16]
K16147	Bacteroid	RHE_RS27870	glgE; starch synthase (maltosyl-transferring) [EC:2.4.99.16]
K00799	MM	RHE_RS05865	GST, gst; glutathione S-transferase [EC:2.5.1.18]
K00799	MM	RHE_RS06130	GST, gst; glutathione S-transferase [EC:2.5.1.18]
K00799	MM	RHE_RS06230	GST, gst; glutathione S-transferase [EC:2.5.1.18]
K00799	MM	RHE_RS11855	GST, gst; glutathione S-transferase [EC:2.5.1.18]
K00799	MM	RHE_RS01425	GST, gst; glutathione S-transferase [EC:2.5.1.18]
K00799	Bacteroid	RHE_RS07560	GST, gst; glutathione S-transferase [EC:2.5.1.18]
K00799	Bacteroid	RHE_RS12380	GST, gst; glutathione S-transferase [EC:2.5.1.18]
K00799	Bacteroid	RHE_RS25110	GST, gst; glutathione S-transferase [EC:2.5.1.18]
K00799	Bacteroid	RHE_RS05070	GST, gst; glutathione S-transferase [EC:2.5.1.18]
K02495	MM	RHE_RS30905	hemN, hemZ; oxygen-independent coproporphyrinogen III oxidase [EC:1.3.98.3]
K02495	MM	RHE_RS29140	hemN, hemZ; oxygen-independent coproporphyrinogen III oxidase [EC:1.3.98.3]
K02495	Bacteroid	RHE_RS30730	hemN, hemZ; oxygen-independent coproporphyrinogen III oxidase [EC:1.3.98.3]
K00817	MM	RHE_RS19480	hisC; histidinol-phosphate aminotransferase [EC:2.6.1.9]
K00817	Bacteroid	RHE_RS30550	hisC; histidinol-phosphate aminotransferase [EC:2.6.1.9]
K00817	Bacteroid	RHE_RS06810	hisC; histidinol-phosphate aminotransferase [EC:2.6.1.9]
K00457	MM	RHE_RS23940	HPD, hppD; 4-hydroxyphenylpyruvate dioxygenase [EC:1.13.11.27]
K00457	Bacteroid	RHE_RS08930	HPD, hppD; 4-hydroxyphenylpyruvate dioxygenase [EC:1.13.11.27]
K01745	MM	RHE_RS24440	hutH, HAL; histidine ammonia-lyase [EC:4.3.1.3]
K01745	Bacteroid	RHE_RS01780	hutH, HAL; histidine ammonia-lyase [EC:4.3.1.3]
K10191	MM	RHE_RS22750	lacK; lactose/L-arabinose transport system ATP-binding protein
K10191	Bacteroid	RHE_RS19645	lacK; lactose/L-arabinose transport system ATP-binding protein
K10111	MM	RHE_RS14795	malK, mtlK, thuK; multiple sugar transport system ATP-binding protein [EC:7.5.2.-]
K10111	MM	RHE_RS27505	malK, mtlK, thuK; multiple sugar transport system ATP-binding protein [EC:7.5.2.-]
K10111	MM	RHE_RS10605	malK, mtlK, thuK; multiple sugar transport system ATP-binding protein [EC:7.5.2.-]
K10111	Bacteroid	RHE_RS25965	malK, mtlK, thuK; multiple sugar transport system ATP-binding protein [EC:7.5.2.-]
K03406	MM	RHE_RS02080	mcp; methyl-accepting chemotaxis protein
K03406	MM	RHE_RS02690	mcp; methyl-accepting chemotaxis protein
K03406	MM	RHE_RS03220	mcp; methyl-accepting chemotaxis protein
K03406	MM	RHE_RS03580	mcp; methyl-accepting chemotaxis protein
K03406	MM	RHE_RS03585	mcp; methyl-accepting chemotaxis protein
K03406	MM	RHE_RS04470	mcp; methyl-accepting chemotaxis protein
K03406	MM	RHE_RS04590	mcp; methyl-accepting chemotaxis protein
K03406	MM	RHE_RS04920	mcp; methyl-accepting chemotaxis protein
K03406	MM	RHE_RS05950	mcp; methyl-accepting chemotaxis protein
K03406	MM	RHE_RS06430	mcp; methyl-accepting chemotaxis protein
K03406	MM	RHE_RS17765	mcp; methyl-accepting chemotaxis protein
K03406	MM	RHE_RS17980	mcp; methyl-accepting chemotaxis protein
K03406	MM	RHE_RS17990	mcp; methyl-accepting chemotaxis protein
K03406	MM	RHE_RS27980	mcp; methyl-accepting chemotaxis protein
K03406	MM	RHE_RS02065	mcp; methyl-accepting chemotaxis protein
K03406	Bacteroid	RHE_RS27360	mcp; methyl-accepting chemotaxis protein
K10112	MM	RHE_RS18950	msmX, msmK, malK, sugC, ggtA, msiK; multiple sugar transport system ATP-binding protein
K10112	MM	RHE_RS22575	msmX, msmK, malK, sugC, ggtA, msiK; multiple sugar transport system ATP-binding protein
K10112	MM	RHE_RS23370	msmX, msmK, malK, sugC, ggtA, msiK; multiple sugar transport system ATP-binding protein
K10112	MM	RHE_RS26890	msmX, msmK, malK, sugC, ggtA, msiK; multiple sugar transport system ATP-binding protein
K10112	MM	RHE_RS28085	msmX, msmK, malK, sugC, ggtA, msiK; multiple sugar transport system ATP-binding protein
K10112	MM	RHE_RS29410	msmX, msmK, malK, sugC, ggtA, msiK; multiple sugar transport system ATP-binding protein
K10112	MM	RHE_RS12565	msmX, msmK, malK, sugC, ggtA, msiK; multiple sugar transport system ATP-binding protein
K10112	Bacteroid	RHE_RS24950	msmX, msmK, malK, sugC, ggtA, msiK; multiple sugar transport system ATP-binding protein
K10112	Bacteroid	RHE_RS28400	msmX, msmK, malK, sugC, ggtA, msiK; multiple sugar transport system ATP-binding protein
K10112	Bacteroid	RHE_RS24520	msmX, msmK, malK, sugC, ggtA, msiK; multiple sugar transport system ATP-binding protein
K01916	MM	RHE_RS06125	nadE; NAD+ synthase [EC:6.3.1.5]
K01916	Bacteroid	RHE_RS06125	nadE; NAD+ synthase [EC:6.3.1.5]
K00459	MM	RHE_RS29235	ncd2, npd; nitronate monooxygenase [EC:1.13.12.16]
K00459	Bacteroid	RHE_RS02555	ncd2, npd; nitronate monooxygenase [EC:1.13.12.16]
K23537	MM	RHE_RS10660	nupA; general nucleoside transport system ATP-binding protein
K23537	Bacteroid	RHE_RS00955	nupA; general nucleoside transport system ATP-binding protein
K10018	MM	RHE_RS24420	occT, nocT; octopine/nopaline transport system substrate-binding protein
K10018	Bacteroid	RHE_RS30295	occT, nocT; octopine/nopaline transport system substrate-binding protein
K00033	MM	RHE_RS12615	PGD, gnd, gntZ; 6-phosphogluconate dehydrogenase [EC:1.1.1.44 1.1.1.343]
K00033	Bacteroid	RHE_RS17825	PGD, gnd, gntZ; 6-phosphogluconate dehydrogenase [EC:1.1.1.44 1.1.1.343]
K22468	MM	RHE_RS02565	ppk2; polyphosphate kinase [EC:2.7.4.1]
K22468	Bacteroid	RHE_RS23870	ppk2; polyphosphate kinase [EC:2.7.4.1]
K00286	MM	RHE_RS15425	proC; pyrroline-5-carboxylate reductase [EC:1.5.1.2]
K00286	Bacteroid	RHE_RS28670	proC; pyrroline-5-carboxylate reductase [EC:1.5.1.2]
K10439	MM	RHE_RS22400	rbsB; ribose transport system substrate-binding protein
K10439	MM	RHE_RS27555	rbsB; ribose transport system substrate-binding protein
K10439	MM	RHE_RS30010	rbsB; ribose transport system substrate-binding protein
K10439	MM	RHE_RS30060	rbsB; ribose transport system substrate-binding protein
K10439	MM	RHE_RS09135	rbsB; ribose transport system substrate-binding protein
K10439	Bacteroid	RHE_RS29865	rbsB; ribose transport system substrate-binding protein
K02968	MM	RHE_RS01805	RP-S20, rpsT; small subunit ribosomal protein S20
K02968	Bacteroid	RHE_RS01805	RP-S20, rpsT; small subunit ribosomal protein S20
K01609	MM	RHE_RS11125	trpC; indole-3-glycerol phosphate synthase [EC:4.1.1.48]
K01609	Bacteroid	RHE_RS11125	trpC; indole-3-glycerol phosphate synthase [EC:4.1.1.48]

* Annotation of genes in the program BlastKoala (Kanehisa et al. 2016), based on the orthology assigns a *K* number.

The ABC components of the sugar transporters were present in MM and bacteroid; i.e., maltose/maltodextrin, galactose, raffinose/stachyose/melibiose, lactose/L-arabinose, sorbitol/mannitol, trehalose/maltose, cellobiose, chitobiose, arabinooligosaccharide. In bacteroids, for monosaccharide transporters, glucose, ribose, galactofuranose, and myo-inositol 1-phosphate were identified, while D-xylose, fructose, rhamnose, myo-inositol, and glycerol were identified in MM (Supplementary Table 2 pathway 37).

The multiplicity of ABC transporters was for seven K numbers (Supplementary Table 3A); for *afuA*, *fbpA*; iron(III) transport system substrate-binding protein; in MM, RHE_RS10880 was identified, and RHE_RS13955 in bacteroid (Table 1). For *occT*, *nocT*, octopine/nopaline transport system substrate-binding protein; in MM, RHE_RS24420 was identified and RHE_RS30295 was expressed in bacteroid (Table 1). The *malK*, *mtlK*, *thuK*; multiple sugar transport system ATP-binding protein [EC:3.6.3.-]; in MM, the proteins RHE_RS10605, RHE_RS14795, RHE_RS27505 were identified, and RHE_RS25965 was expressed in bacteroid (Table 1). The *msmX*, *msmK*, *malK*, *sugC*, *ggtA*, *msiK*; multiple sugar transport system ATP-binding protein, in MM represented by RHE_RS12565, RHE_RS18950, RHE_RS22575, RHE_RS23370, RHE_RS26890, RHE_RS28085, RHE_RS29410 were found, while the RHE_RS24520, RHE_RS24950 and RHE_RS28400 were identified in bacteroid (Table 1). For the *lacK*; lactose/L-arabinose transport system ATP-binding protein, sn-glycerol-3-phosphate ABC transporter, the ATP-binding protein UgpC in MM RHE_RS22750 and in bacteroid RHE_RS19645 were identified (Table 1). The *rbsB*; ribose transport system substrate-binding protein is represented by the isoenzymes RHE_RS09135, RHE_RS22400, RHE_RS27555, RHE_RS30010, RHE_RS30060 in MM, and RHE_RS29865 was expressed in bacteroid (Table 1). For the *nupA*, general nucleoside transport system ATP-binding protein in MM RHE_RS10660 and in bacteroid RHE_RS00955 were identified (Table 1; Supplementary Table 2, pathway 37 and Supplementary Table 3A). Once multiplicity was detected in MM and bacteroid, a wide search for multiplicity in data was performed. Interestingly, from the 101 proteins representing 48 unique K numbers (a K number may have more than one protein), 34 isoenzymes were identified that cover 60 metabolic pathways (Table 1; Supplementary Table 3A). In synthesis, multiplicity in only one enzyme was equally found for other metabolic processes such as for peptidases, inhibitors, amino acids and related enzymes, messenger ARN biogenesis, ribosome, ribosome biogenesis, transfer ARN biogenesis, translation factors, chaperones and folding catalysis, DNA replication proteins, DNA repair, and recombination proteins. While other pathways had multiplicity in two different enzymes, e.g., lipid biosynthesis proteins, mitochondrial biogenesis, two-component system, and bacterial motility proteins. Furthermore, multiplicity for three enzymes in a pathway was also detected, e.g., glutathione metabolism (Supplementary Table 2 pathway 3, and Supplementary Table 3A), for *pepA*, leucyl aminopeptidase [EC:3.4.11.1] enzyme, the RHE_RS01080 was expressed in MM, while in bacteroid the RHE_RS07430 was identified (Table 1). For the *gst*, glutathione S-transferase [EC:2.5.1.18] in MM RHE_RS01425, RHE_RS05865, RHE_RS06130, RHE_RS06230, and RHE_RS11855 were identified and in bacteroids, RHE_RS05070, RHE_RS07560, RHE_RS25110, and RHE_RS12380 were identified (Table 1). As well as, the *gntZ*, 6-phosphogluconate dehydrogenase [EC:1.1.1.44 1.1.1.343] in MM RHE_RS12615 and in the bacteroid RHE_RS17825 were expressed (Table 1). Multiplicity was also found in 5 transcription regulators and 16 transporters (Supplementary Table 3A).

From this data, there are some relevant points; it has been shown that during symbiosis of *R. leguminosarum*, bacteroids become auxotrophic for branched-chain amino acids, and their supply depends on the leguminous pea plant (Prell et al. 2009). In contrast, in *R. etli* CFN42, for valine, leucine, and isoleucine biosynthesis, 9, 3, and 3 enzymes were detected in MM, bacteroid, and Nch, respectively (Supplementary Table 2 pathway 6, and Supplementary Table 3A), suggesting a functional pathway in *R. etli* CFN42. Multiplicity was also found for the β-hydroxybutyrate dehydrogenase enzyme in *B. japonicum* USDA110, two isoforms were exclusively expressed in free-living conditions and a new isoform was expressed in nodule proteomes (Sarma and Emerich 2006). Another difference between the symbiosis of *R. etli* CFN42 is the expression of a great number of ABC sugars transporters which does not seem to be expressed in the symbiosis of *B. japonicum* and *S. meliloti*, reviewed in (Sarma and Emerich 2006). Also, this data confirmed two different systems for defense against oxidative stress for *R. etli* CFN42 (Resendis-Antonio et al. 2011), which is also observed in *S. meliloti* 1021 (see below), one prevailing in free-living conditions and the other in symbiosis. As shown, the multiplicity of genes for an enzyme is a generality in the cellular functioning of *R. etli* CFN42 in free-living conditions and symbiosis, clearly showing a greater genetic redundancy for enzymes expressed in MM than in symbiosis that may or may not be paralogous genes (Supplementary Table 3A). Additionally, a contrasting analysis of function assigned to the genes between the KO Orthology database (Kanehisa et al. 2016; Kanehisa 2017) and the NCBI database^6^ was performed from the 48 unique K numbers covering 101 and 74 proteins for MM and bacteroid, respectively (Table 1); only four K numbers from *R. etli* CFN42; K01684, K02433, K00459, and K10439 were different, showing a great coincidence between the two methods (see shaded green rows; Supplementary Table 3A). When genes with the same annotated function exist, phenotypic change of a bacterium is not present by loss of function of a gene copy; it is called “Robustness,” which is the ability to maintain the function when there is a change, as it was from free life to symbiosis (González et al. 2006; Diss et al. 2014), and they are maintained by context-dependent differences (Putty et al. 2013). These data suggest that when *R. etli* CFN42 is in free life and under symbiotic conditions, there is a metabolic adaptation, implying distinct transcriptional regulation for these genes.

### Isoenzymes in *Sinorhizobium meliloti* 1021

An identical analysis was performed with a peptone yeast-rich medium and bacteroid transcriptome data from *S. meliloti* 1021 to search for isoenzymes. Significant data were selected with two parameters, log ≥0.96 and with software with *p* ≥ 0.05 (Barnett et al. 2004). In contrast to *R. etli* CFN42, *S. meliloti* only showed 7K genes for isoenzymes; SMc03978 *tkt2* for transketolase was expressed in TY, while in bacteroids, SMc00270 was expressed (Supplementary Table 3B). The protein SMc03994 for the 30S ribosomal protein S21 was present in TY medium, while SMc04320 for the 30S ribosomal protein was present in bacteroids. The SMa0744 protein GroEL was translated in TY and was substituted by the SMa0124 GroEL protein in bacteroids. Moreover, SMc02897 for the cytochrome C transmembrane protein was expressed in TY medium, and the equivalent activity was substituted by the SMc01981 cytochrome C protein in the bacteroid. Moreover, as shown for *R. etli* CFN42 for defense against oxidative stress in MM and bacteroids, *S. meliloti* 1,021 in TY medium expressed five glutathione-S transferases, SMc00097 (gst2), SMc00383 (gst3), SMc00407 (gst4), SMc03082 (gst8), and SMc00036 (gst1). This activity was performed by the SMc01443 (gst6) glutathione-S transferase protein in bacteroids (Supplementary Table 3B). These data suggest that an alternative system for defense against oxidative stress also exists in *S. meliloti* 1021 bacteroids. There were contrasting low K numbers in *S. meliloti* 1021 compared with the *R. etli* CFN42 genome, and these data probably have a bias from a different method for the selection of significant data between these bacteria.

### Transcriptional regulatory network

Taking advantage of the RhizoBindingSites database^7^ (Taboada-Castro et al. 2020), networks were constructed for MM and bacteroid protein profiles with the application “Prediction of regulatory network” (see “Materials and Methods” section). A three-step method to build a network was implemented (see “Materials and Methods” section). In the second step (see methods), Clustered-TF genes obtained with the matrix-clustering analysis, were used as input in the application of RhizoBindingSites database “Prediction of regulatory networks” with the option “auto”, to corroborated potential TF gen-target relationships. The cluster_34 and cluster_195 from MM, and cluster_97 and cluster_112 from bacteroid were chosen. For cluster_34, all the genes had a TF gene-target relationship. Indeed, for cluster_195, 22 out of 27 genes were connected (Supplementary Table 1G). For cluster_97, 21 of 26 genes were connected and for cluster_112, 21 from 22 genes were connected (Supplementary Table 1H). These data showed that the matrix of a clustered-TF, has homology to a matrix of the target gene. In consequence, the matrices from both, the TF´s and the gene-target are conserved in their respective orthologs genes, because upstream regulatory regions of the orthologs genes were used to deduce the matrices (Taboada-Castro et al. 2020). Suggesting, this conservation is by a compromised function of the motifs for the TF and the target genes and not by chance. Then, the transcriptional regulatory networks of MM and bacteroid protein profiles are constructed with motifs interspecies conserved. The quality of the MM, bacteroid, clustered-TF-MM and clustered-TF-BACTEROID networks, which are data of the three-step method, was compared by analyzing the number of interactions per p-value range. The number of interactions of p-values with low stringency decreased, and those with a higher stringency in the network from clustered-TF-MM and clustered-TF-BACTEROID increased, meaning that there was an enrichment of interactions with high stringency p-value levels (see “Materials and Methods” section, Figure 5), emphasizing that most of the TF gene–target interactions eliminated from clustered-TF-MM and clustered-TF-BACTEROID had low stringency p-values. These data confirmed that clustered matrices of genes are strongly related to the structure of a network, and these genes probably represent hubs. We expect this new method will be helpful for the depuration of regulons from any potential TF gene-target data, since it provides data with the highest level of restriction as possible, based on coexpression of the TF´s, instead of arbitrarily imposing a threshold to determine the significance of data. The number of clusters per network was 654 and 92 for Clustered-TF-MM and Clustered-TF-BACTEROID, respectively. Moreover, 654 proteins, including 93 TFs for Clustered-TF-MM, and 246 TFs for Clustered-TF-BACTEROID, including 46 TF proteins, were identified (Supplementary Tables 4A-B). These expected regulatory networks had 5,091 and 1,114 TF gene–target relationships for MM and bacteroid, respectively, the hypothetical regulons are available (Supplementary Tables 4 A-B). Additionally, to determine whether the matrices of these networks detect motifs in the upstream regulatory region of their corresponding orthologous genes in the order Rhizobiales, an analysis with a footprint-scan method was conducted (Nguyen et al. 2018). These data showed a great number of motifs detected with these matrices even for phylogenetically distant species of *R. etli* CFN42 (data not shown), suggesting that this conservation of motifs occurs by a functional compromise.

**Figure 6:**
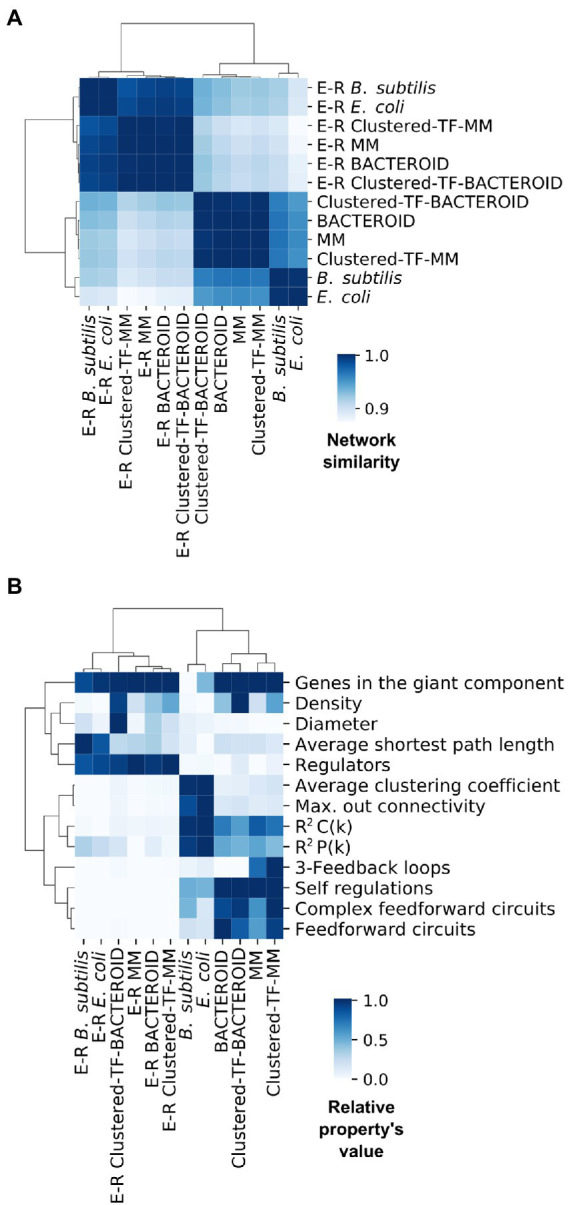
**(A)** Network similarity and **(B)** Symmetric properties of experimental networks from *Escherichia coli*, *Bacillus subtilis* and predicted networks from *Rhizobium etli* CFN42; MM, Clustered-TF-MM, BACTEROID and Clustered-TF-BACTEROID compared with 1000 Erdös-Rényi random networks.

We wondered how our inferred networks assess against known curated networks. As no curated network is available for *R. etli*, inspired by recent work showing that assessing using network structural properties provides results consistent with using a gold-standard (Zorro-Aranda et al. 2022), we performed a pairwise comparison *via* correlation of the normalized structural profiles of two well-curated regulatory networks, *E. coli* and *B. subtilis*, as positive control and a background of Erdös-Rényi parametrized random networks as a negative control (Figure 6A; “Materials and Methods” section).

Comparing these properties showed that negative control networks were clearly segregated from the experimental and inferred biological networks, showing that experimental and inferred biological networks were more similar (Figure 6A). Consequently, the inferred biological networks were not random. We then analyzed these structural property profiles of the networks using mix-max scaling across networks to maximize the differences (Figure 6B). We confirmed the segregation of the negative controls from the biological and experimental networks, which means that our networks are not random and that the experimental and inferred networks were more similar. The density was higher for the inferred networks than for the experimental networks. Between the inferred networks, the density of bacteroid and clustered-TF-BACTEROID was higher than that of the MM and clustered-TF-MM networks. The density of Clustered-TF-MM and Clustered-TF-BACTEROID could be increased due to grouping the matrices with the aforementioned matrix-clustering strategy (Figure 6B; “Materials and Methods” section).

The scale-free properties of the inferred networks were contrasted to the experimental networks of *E. coli* and *B. subtilis* by two alternative methods: robust linear regression and maximum likelihood estimation. Currently, the transcriptional regulatory network of a *Rhizobium* strain is unknown. A bioinformatic study based on functional relationships from the PROLINKS and STRING databases showed a scale-free interaction network and modularity for *Sinorhizobium meliloti* (Rodriguez-Llorente et al. 2009). However, they considered greater, more significant proteins than this study. Consistently, many genes showed a modular organization in a metabolic network of *R. etli* CFN42 with proteomic, transcriptomic, and metabolomic data (Resendis-Antonio et al. 2012). Additionally, there are more 3-feedback loops in the MM and Clustered-TF-MM networks than in the bacteroid, Clustered-TF-BACTEROID, *E. coli*, and *B. subtilis* networks. The self-regulation, complex feed-forward circuits, and feed-forward circuits from inferred networks were higher than the experimental ones (Figure 6B). Self-regulation is higher for inferred networks than experimental networks because the RhizoBindingSites database was built only with genes whose matrices could recognize a motif in their upstream promoter region.

The average clustering coefficient, maximum out connectivity, cluster coefficient *R*^2^ C(k), and connectivity distribution *R*^2^ P(k) were higher for the experimental than for inferred biological networks, implying that the inferred networks have an atypical very low modularity. As previously shown in several organisms (Freyre-González et al. 2008; 2012; Freyre-González and Tauch 2017; Escorcia-Rodríguez et al. 2021), the Natural Decomposition Approach (NDA) reveals that bacterial regulatory networks shape a diamond-like, three-tier, hierarchy where global TFs govern modules, and the local response of these modules is integrated at the promoter level by intermodular genes, whereas modules are shaped by local TFs and structural genes (Freyre-González et al. 2022). An analysis of our predicted networks using the NDA showed a hierarchy only composed of global TF and basal machinery, where neither modules nor intermodular genes could be identified (data not shown). These could be a consequence of the atypical high density of the inferred network, as this causes the networks to be more interconnected than usual.

**Figure 7:**
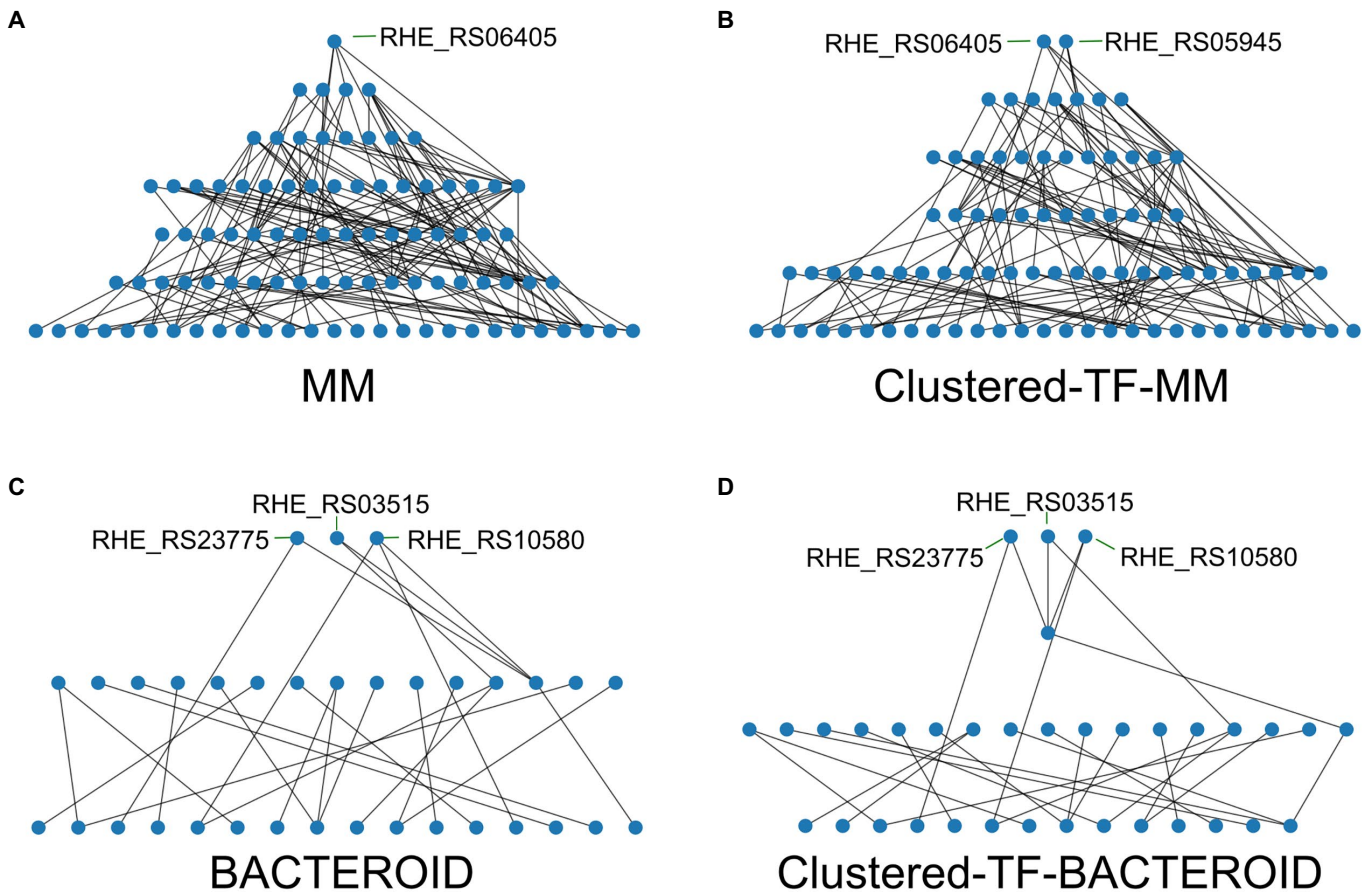
Hierarchy of the transcriptional regulators (TF’s) of the networks; **(A)** Minimal medium, **(B)** Clustered-TF-MM, **(C)** BACTEROID, and **(D)** Clustered-TF-BACTEROID from *Rhizobium etli* CFN42.

As we found that in our networks the integrative layer composed of the intermodular genes is absent, we leverage that it has been previously shown that regulatory networks are mainly descendent (Ma et al. 2004) but there are still some feedback circuits (Freyre-González et al. 2008, 2012). We unveil a hierarchy of the inferred networks by removing the top-down edges, thus eliminating feedback, and applying a topological sorting algorithm to the predicted network (Figure 7; Supplementary Table 5 and “Materials and Methods” section). Our strategy maintains the global structure of the network to reveal the hierarchy. Besides structural nodes, no other nodes are removed, and ‘bottom-up’ edges can be added back to the hierarchy to reveal the feedback among layers and reconstruct the original network.

The hierarchy of the MM network showed that RHE_RS06405 (MucR family) is at the top. Under the top, there are four genes: RHE_RS25725 (LysR family), RHE_RS16230 (ROK family), RHE_RS05945 (LuxR family), and RHE_RS02355 (ROK family; Figure 7A; Supplementary Table 5). In contrast, for the Clustered-TF-MM network (Figure 7B; Supplementary Table 5), RHE_RS06405 (MucR family) and RHE_RS05945 (LuxR family) were at the top, and under the top seven TFs identified, RHE_RS25725 (LysR family) and RHE_RS20575 (*carD* family), a CarD protein, pertaining to the CarD_CdnL_TRCF family of TFs described in *Mycobacterium tuberculosis* 2018, binds to RNA polymerase and activates transcription by stabilizing the transcription initiation complex, elongation or termination steps, and deletion of N-terminal residues hampers amyloid formation (Kaur et al. 2018). It was shown that the interaction of CarD with the RNAP beta-subunit is responsible for mediating *M. tuberculosis* viability, rifampicin resistance, and pathogenesis. It is a highly expressed protein, also induced by multiple stresses. Transient depletion of CarD makes *M. tuberculosis* more sensitive to being killed by reactive oxygen species, and its mutation abolishes persistence in mice (Weiss et al. 2012). In addition, RHE_RS16230 (ROK family), RHE_RS02355 (ROK family), RHE_RS17050 (response regulator), RHE_RS01875 (helix-turn-helix transcriptional regulator), and RHE_RS00415 (TetR family) were identified. For bacteroid and clustered TF-BACTEROID, the hierarchy of transcriptional regulatory networks showed the same three TFs at the top; RHE_RS23775 (NAC, nitrogen assimilation transcriptional regulator). In *Escherichia coli,* the *nac* and *glnK* promoters were strongly activated when cells stopped growing, and ammonium became scarce (Atkinson et al., 2002), as well as RHE_RS03515 (substrate-binding domain) and RHE_RS10580 (LacI family DNA-binding transcriptional regulator; Figures 7C,D; Supplementary Table 5). For MM and clustered-TF-MM transcriptional regulatory networks, seven and six different levels of regulation are shown, respectively (Figures 7A,B; Supplementary Table 5). In contrast, for bacteroid and clustered-TF_BACTEROID, only three and four levels of regulation were shown, respectively (Figures 7C,D; Supplementary Table 5).

### Inferred transcriptional regulation of isoenzymes in MM and bacteroids

TF gene–target relationships for genes coding for isoenzymes in the MM, Clustered-TF-MM and bacteroid, Clustered-TF-BACTEROID networks were inferred (Supplementary Table 6). The transcriptional regulator per locus tag is found in the column E headed “Matrix_ID” with the RHE_RS13345_m5 format (Supplementary Table 6), see “Materials and Methods” section. It is shown by the enzyme PGD, *gnd*, *gntZ*; 6-phosphogluconate dehydrogenase [EC:1.1.1.44 1.1.1.343] (Supplementary Table 6, column AK), the isoenzymes RHE_RS12615 and RHE_RS17825 were expressed in MM and in the bacteroid (Supplementary Table 6, column B), respectively. In MM, the transcriptional regulator is RHE_RS13345_m5 (Supplementary Table 6, column E) with a *p*-value of 1.7e-5 (Supplementary Table 6, column K), and in the bacteroid, it is RHE_RS27925_m4 (Supplementary Table 6, column E) with a *p*-value of 0.18e-4 (Supplementary Table 6, column K). However, there are no data with respect to the Clustered-TF-MM and Clustered-TF-BACTEROID networks (Supplementary Table 6, columns S–Z) due to a reduction of TF’s by the matrix-clustering analysis. Therefore, *fabG*, OAR1; 3-oxoacyl-[acyl-carrier protein] reductase [EC:1.1.1.100] (Supplementary Table 6, columns A–K) enzyme for fatty acid biosynthesis, we identified RHE_RS05335 and RHE_RS06685 in MM, and RHE_RS19755 in bacteroid (Supplementary Table 6, column B), which are potentially regulated by the TFs RHE_17755_m2, RHE_RS30790_m1 and RHE_RS23180_m2 (Supplementary Table 6, columns E and S), with *p*-value of 7.30E-07, 1.20E-06 and 2.60E-06 (Supplementary Table 6, columns K and Y), respectively, for MM, bacteroid and Clustered-TF-MM and Clustered-TF-BACTEROID networks, showing for this enzymatic step that the multiplicity also corresponds with a different TF involved in transcriptional regulation. Note that each network contains its own *p*-value (see Supplementary Table 6, columns K and Y). For a better choice of a TF gene-target, data from a clustered-TF network and a low *p*-value as possible is desirable. From here on, in this discussion, the *p*-value located in Supplementary Table 6, column K, for not clustered networks and Supplementary Table 6, column Y, for clustered networks will be omitted. Concerning the enzyme D-threo-aldose 1-dehydrogenase [EC:1.1.1.122], in MM and bacteroid, the isoenzymes RHE_RS02500 and RHE_RS28605 were expressed, and the inferred TFs were RHE_RS22090_m3 and RHE_RS03515_m5, respectively, showing a potentially distinct TF-dependent physiological condition, but incomplete data were obtained for Clustered-TF networks (Supplementary Table 6, columns S–Z). In the case of *gcvT* and AMT, aminomethyltransferase enzyme [EC:2.1.2.10] in MM expressed RHE_RS28340 and two isoenzymes in bacteroid; RHE_RS26195 and RHE_RS26150 were expressed, and their corresponding TFs RHE_RS28340_m3, RHE_RS00285_m4, and RHE_RS05730_m3 were deduced, respectively, for both non and clustered-TF networks, supporting the suggestion of distinct regulation of these genes in MM and bacteroid (Supplementary Table 6).

Most likely, the microaerobic conditions and the metabolic functions prevailing in the bacteroid (fixing nitrogen), in comparison with the bacteria cultivated in MM (free life), induce specific strategies against oxidative stress, e.g., for the case of the enzyme GST, *gst*; glutathione S-transferase [EC:2.5.1.18], in MM RHE_RS0630 and RHE_RS11855 proteins were expressed for the MM network, while in bacteroid, the RHE_RS12380 was identified with a Clustered-TF network, with the TFs RHE_RS06135_m4, RHE_RS27645_m3 in MM and RHE_RS08350_m3 in the bacteroid (Supplementary Table 6). Regarding *yghU* and *yfcG*, GSH-dependent disulfide-bond oxidoreductase [EC:1.8.4.-] in MM and bacteroid isoenzymes RHE_RS22490 and RHE_RS04155, respectively, were expressed, potentially under the transcriptional control of TFs RHE_RS12670_m4 and RHE_RS12205_m4, respectively (Supplementary Table 6). For these proteins involved in the repair of oxidized proteins, a different transcriptional regulation is suggested in MM and bacteroid and clustered-TF-MM and clustered-TF-BACTEROID networks. Iron transport is relevant for metabolism regarding *afuA* and *fbpA,* which encode the iron(III) transport system substrate-binding protein and express the isoenzymes RHE_RS10880 and RHE_RS13955 in MM and bacteroid, respectively, with the TFs RHE_RS28340_m4 and RHE_RS16205_m5, respectively, for MM and bacteroid networks (Supplementary Table 6), our data suggest two distinct metabolic strategies for transport of iron in MM (free life) and bacteroid (nitrogen fixing) conditions. It has been discussed that transport is specific for these metabolic stages (Sarma and Emerich 2006); indeed, this was supported for amino acid transport regarding ABC.PA. S; the polar amino acid transport system substrate-binding protein, in MM RHE_RS02695, RHE_RS11720, and RHE_RS27400, and in bacteroid RHE_RS07475 and RHE_RS27430 were expressed, potentially regulated by the TFs RHE_RS30745_m3, RHE_RS24110_m2, RHE_RS14135_m3 and RHE_RS18525_m2, RHE_RS26505_m5, respectively. All these data were clustering TF-associated, showing distinct TFs for each metabolic condition (Supplementary Table 6). Concerning transcriptional regulators, *lacI* and *galR* belonging to the LacI family in Clustered-TF-MM RHE_RS03090, RHE_RS12585, RHE_RS17450, RHE_RS23055, RHE_RS23350, and RHE_RS27560 were expressed in comparison with Clustered-TF-BACTEROID, where the following proteins were identified: RHE_RS03515, RHE_RS15245, and RHE_RS27525. Probably some genes are expressed because they respond to different physiological conditions with the aim of regulating different groups of genes. The inferred TFs for these genes were RHE_RS03090_m2, RHE_RS12585_m4, RHE_RS17450_m4, RHE_RS23055_m3, RHE_RS23350_m1, RHE_RS27560_m3, and RHE_RS03515_m5 for cluster-TF-MM, as well as, RHE_RS24095_m3 for bacteroid network, and RHE_RS03515_m5, RHE_RS27525_m2 for clustered-TF-BACTEROID, respectively (Supplementary Table 6). These data support the idea that isoenzymes have distinct regulations. For the ABCB-BAC ATP-binding cassette, subfamily B, bacterial beta-(1 –> 2)glucan export ATP-binding/permease NdvA protein, the proteins RHE_RS20455 and RHE_RS10390 were expressed in MM and bacteroid, respectively, with the TFs RHE_RS23325_m5 and RHE_RS26875_m3, for both not and clustered-TF were inferred, respectively, supporting a differential transcriptional regulation (Supplementary Table 6). Multiple *rbsB*; ribose transport system substrate-binding protein transporters, RHE_RS09135, RHE_RS22400, RHE_RS27555, RHE_RS30060, and RHE_RS30060 were expressed in MM, while RHE_RS29865 was identified in bacteroid; the data suggested that they were under the Clustered-TF transcriptional control of RHE_RS22090_m2, RHE_11740_m2, RHE_RS27560_m3, RHE_RS04690_m3 and RHE_RS02355_m4 and the not clustering TF associated RHE_RS10580_m1, respectively (Supplementary Table 6). Currently, it is not clear whether the plant supplies sugar to the bacteroid. A metabolome study showed that GDP-mannose and GDP-galactose were identified to be 7.4 times higher in bacteroids than in bacteria grown in MM (data not shown); in the opposite sense, proteins for these pathways were significantly higher in MM than in bacteroids (Supplementary Table 2, pathway 10). The two-component system, OmpR family response regulator proteins RHE_RS06580, RHE_RS10890, RHE_RS12325, and RHE_RS21355, were detected in MM and RHE_RS29195 in bacteroid, with TFs RHE_RS06580_m4, RHE_RS05790_m3, RHE_RS12325_m2, and RHE_RS21355_m3, for the proteins expressed in MM and RHE_RS29195_m2 for bacteroid, respectively (Supplementary Table 6), for non and clustered-TF networks, showing that multiplicity has a distinct potentially transcriptional regulation. The *nodD* LysR family transcriptional regulator recognizes a *nod-box* for transcriptional activation (Mao et al. 1994). We have demonstrated the function of the *nodD* transcriptional regulators by supplementation of MM with the flavonoid naringenin, which induced the synthesis of the nodulation factor (Meneses et al. 2017). The *nodD* genes RHE_RS30790, RHE_RS31010, and RHE_RS31005 proteins were expressed in Clustered-TF-MM and Clustered-TF-BACTEROID, respectively, probably under the transcriptional control of the inferred TFs RHE_RS30790_m2, RHE_RS12670_m4 Clustered-TF and RHE_RS20460_m2, not Clustered-TF, respectively (Supplementary Table 6). It was demonstrated that lysR *nodD* genes were autoregulated (Hu et al. 2000), as was *in silico* shown for the NodD RHE_RS30790 (Taboada-Castro et al. 2020); in addition, the *nodD* genes may be regulated by other TFs (Barnett and Long 2015), as was inferred for *nodD* RHE_RS31005 (Taboada-Castro et al. 2020). Altogether, these data suggested that in addition to specific isoenzymes expressed in a condition-dependent manner, they are potentially under specific transcriptional regulatory control. This data suggested how *R. etli* CFN42 re-program its transcriptional regulatory network to be metabolically adapted for growth in MM or in the symbiosis with the leguminous plant.

## Conclusion

A free-living and symbiotic proteomic study from *R. etli* CFN42 were performed. A lower number of proteins per pathway in bacteroids than in MM was found, and approximately 30 and 20% of proteins for some metabolic pathways were detected in MM and bacteroids with respect to the genomic content, respectively. A mapping of classified proteins based on orthology allowed us to discover the presence of isoenzymes specific for growth in minimal medium and symbiosis with deduced specific transcriptional regulation. In addition to the metabolic pathways identified, genes for the degradation of environmental compounds were detected in MM and symbiotic proteomes. In contrast, a low number of isoenzymes were found in the *S. meliloti* transcriptome data. Taking advantage of the RhizoBindingSites database, which contains inferred TF gene–target relationships of *R. etli* CFN42 and eight additional symbiotic species, a method was implemented to construct transcriptional regulatory networks for these metabolic conditions. An inferred clustered TF gene network was constructed with motifs highly conserved in the upstream regulatory regions of the genes that are also conserved in the orthologous genes from each gene.

This pioneer bioinformatic framework is an important reference to obtain basic information on the genetic circuitry to increase knowledge about an experimental transcriptional regulatory network. Given the changing climate conditions, experimental validation of these genetic circuits for remodeling the metabolic pathways to optimize the SNF of *R. etli* CFN42 is the next step.

## Data availability statement

The authors acknowledge that the data presented in this study must be deposited and made publicly available in an acceptable repository, prior to publication. Frontiers cannot accept a manuscript that does not adhere to our open data policies.

## Author contributions

HT-C, JG, and SE-G conceived the idea. JG, HT-C, JF-G, JE-R, LG-C, and SE-G designed the analysis. HT-C, JG, JF-G, and SE-G analyzed the results and drafted the manuscript. SE-G revised the manuscript. All authors contributed to the article and approved the submitted version.

## Funding

Part of this work was supported by the Programa de Apoyo a Proyectos de Investigación e Innovación Tecnológica (PAPIIT-UNAM), grants IN 213522 to SE-G and IN202421 to JF-G. JE-R is a doctoral student from Programa de Doctorado en Ciencias Biomédicas, Universidad Nacional Autónoma de México (UNAM); he received fellowship 959406 from CONACYT.

## Conflict of interest

The authors declare that the research was conducted in the absence of any commercial or financial relationships that could be construed as a potential conflict of interest.

## Publisher’s note

All claims expressed in this article are solely those of the authors and do not necessarily represent those of their affiliated organizations, or those of the publisher, the editors and the reviewers. Any product that may be evaluated in this article, or claim that may be made by its manufacturer, is not guaranteed or endorsed by the publisher.

## Footnotes

^1^http://rhizobindingsites.ccg.unam.mx/ (accessed September 8, 2022).

^2^http://www.pantherdb.org/ (accessed September 8, 2022).

^3^https://www.genome.jp/kegg/tool/map_pathway.html (accessed September 8, 2022).

^4^https://www.kegg.jp/blastkoala/ (accessed September 8, 2022).

^5^https://www.genome.jp/kegg/ko.html (accessed September 8, 2022).

^6^https://www.ncbi.nlm.nih.gov/genome/browse/#!/proteins/827/383937%7CRhizobium%20etli/ (accessed September 8, 2022).

^7^http://rhizobindingsites.ccg.unam.mx/ (accessed September 8, 2022).

## Supplementary material

SUPPLEMENTARY TABLE 1 Method for network construction A-I.

SUPPLEMENTARY TABLE 2 Metabolic pathways of the proteins expressed in Minimal medium, bacteroid and present in both conditions from *Rhizobium etli* CFN42.

SUPPLEMENTARY TABLE 3 Isoenzymes from Rhizobium etli CFN42 and *Sinorhizobium meliloti* 1021.

SUPPLEMENTARY TABLE 4 Predicted second transcriptional regulatory network with matrix-clustering of MM and Bacteroid profiles from *Rhizobium etli* CFN42.

SUPPLEMENTARY TABLE 5 Predicted Transcription Factor hierarchy in networks of MM, Clustered-TF-MM, BACTEROID and Clustered-TF-BACTEROID from *Rhizobium etli* CFN42.

SUPPLEMENTARY TABLE 6 Isoenzymes with inferred Transcriptional regulation of non-clustered and clustered networks from *Rhizobium etli* CFN42.
