# Supplementary material for: Rhizobium etli CFN42 proteomes showed isoenzymes in free-living and symbiosis with a different transcriptional regulation inferred from a transcriptional regulatory network
Source: Front Microbiol. 2022 Oct 13;13:947678. doi: 10.3389/fmicb.2022.947678 (PMC9611204; doi:10.3389/fmicb.2022.947678)
Supplement: SUPPLEMENTARY TABLE 2 — Metabolic pathways of the proteins expressed in Minimal medium, bacteroid and present in both conditions from Rhizobium etli CFN42. [file Data_Sheet_1.docx]

Figure 1.- **Metabolic pathways in MM, Bacteroid, and in both MM and Bacteroid from *R. etli*** **CFN42.**

Pathway 1.- Histidine Metabolism

Pathway 2.- Inositol phosphate metabolism

Pathway 3.- Glutathione metabolism

Pathway 4.- Sulfur relay system

Pathway 5.- Glycine, serine and threonine metabolism

Pathway 6.- Valine, leucine, isoleucine biosynthesis

Pathway 7.- Valine, leucine, isoleucine degradation

Pathway 8.- Porphyrin and chlorophyll metabolism

Pathway 9.- Trytophan metabolism

Pathway 10.- Fructose and mannose metabolism

Pathway 11.- Galactose metabolism

Pathway 12.- Propanoate metabolism

Pathway 13.- Pantothenate and CoA Biosynthesis

Pathway 14.- Pentose and glucuronate interconversions

Pathway 15.- Pentose phosphate pathway

Pathway 16.- Purine metabolism

Pathway 17.- Pyrimidine metabolism

Pathway 18.- Pyruvate metabolism

Pathway 19.- Glycolysis- gluconeogenesis

Pathway 20.- TCA cycle

Pathway 21.- Synthesis and degradation of ketone bodies

Pathway 22.- Vitamin B 6 metabolism

Pathway 23.- Ubiquinone and other terpenoid-quinone biosynthesis

Pathway 24.- Oxidative phosphorylation

Pathway 25.- Nicotinate and nicotinamide metabolism

Pathway 26.- Phosphonate and phosphinate metabolism

Pathway 27.- DNA replication

Pathway 28.- terpenoid backbone biosynthesis

Environmental metabolism

Pathway 29.- Chlorocyclohexane and chlorobenzene degradation

Pathway 30.- Chloroalkane and chloroalkene degradation

Pathway 31.- Drug metabolism and other enzymes

Pathway 32.- Drug metabolism cytochrome P450

Pathway 33.- Naphthalene degradation

Pathway 34.- Novobiocin biosynthesis

Pathway 35.- Vitamin B 6 metabolism

Pathway 36.- Two component systems

Pathway 37.- ABC transporters


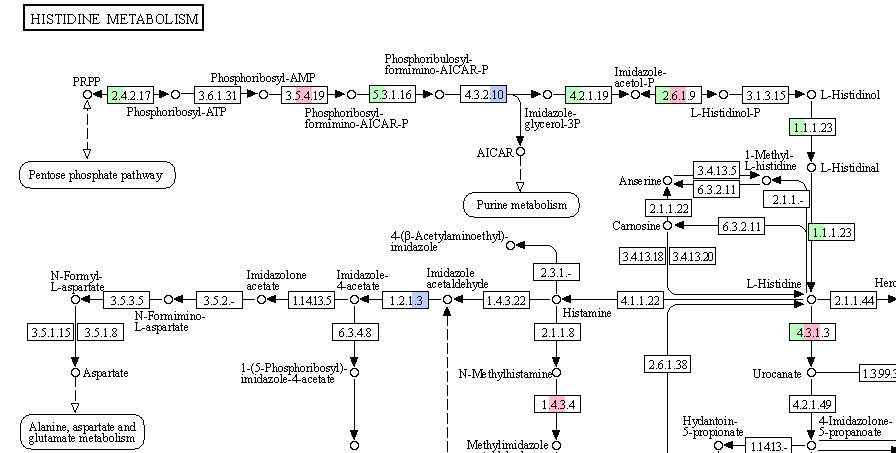


MM; Green, Bacteroide; Pink, With no change in expression; Purple. Highlighting, when two colors are in the enzymatic reaction, it means there are two isozymes for this enzymatic step.

Pathway 1


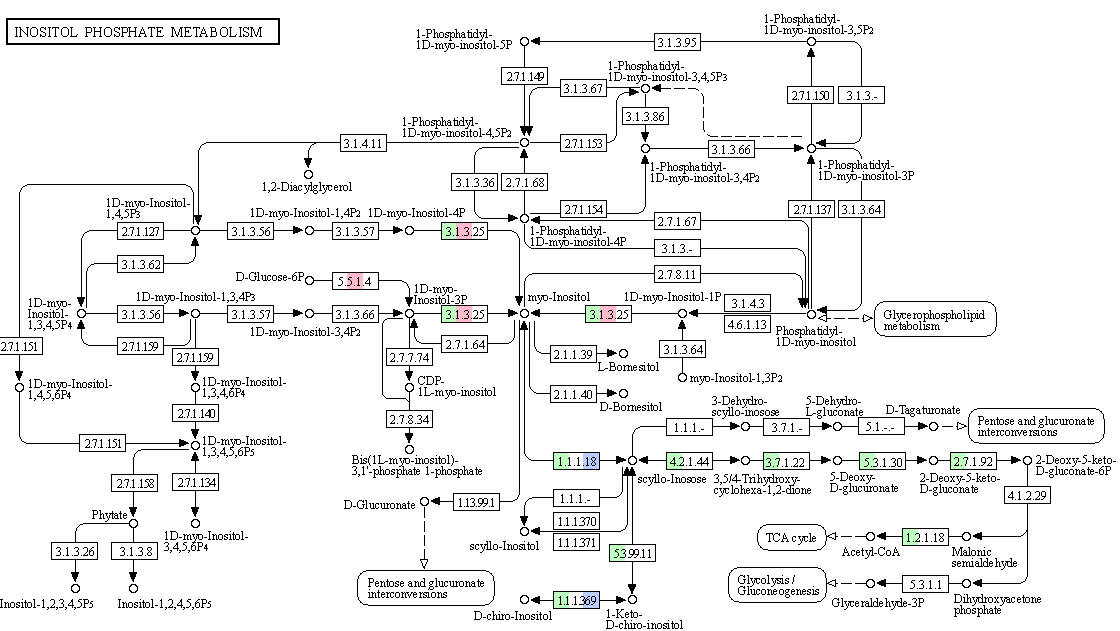


MM; Green, Bacteroide; Pink, With no change in expression; Purple. Highlighting, when two colors are in the enzymatic reaction, it means there are two isozymes for this enzymatic step.

Pathway 2


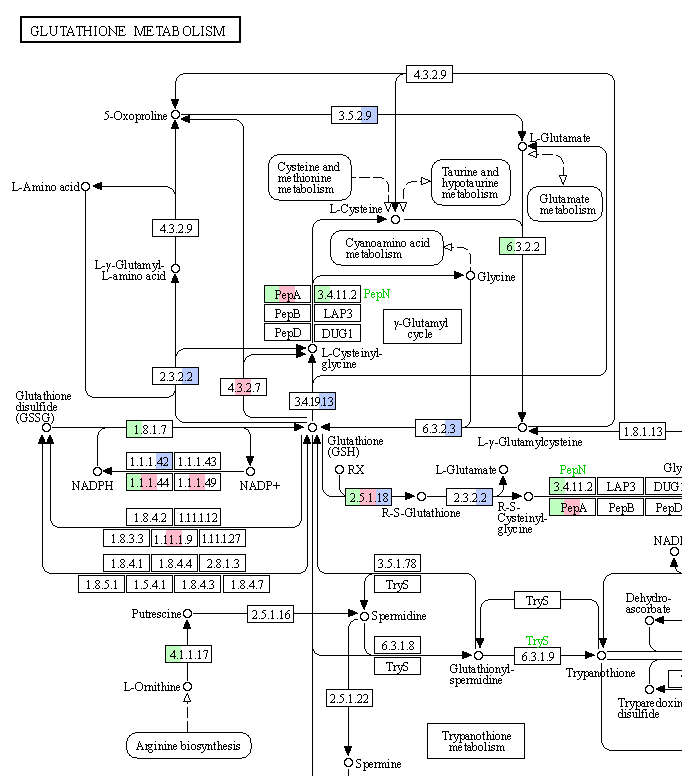


MM; Green, Bacteroide; Pink, With no change in expression; Purple. Highlighting, when two colors are in the enzymatic reaction, it means there are two isozymes for this enzymatic step.

Pathway 3


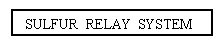


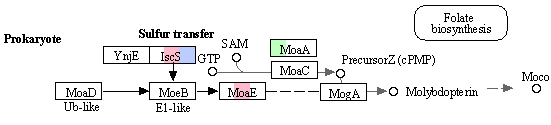


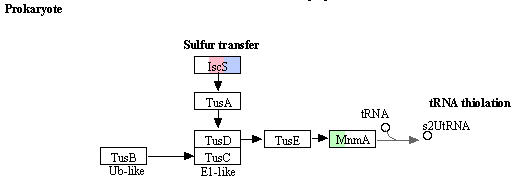


MM; Green, Bacteroide; Pink, With no change in expression; Purple. Highlighting, when two colors are in the enzymatic reaction, it means there are two isozymes for this enzymatic step.

Pathway 4


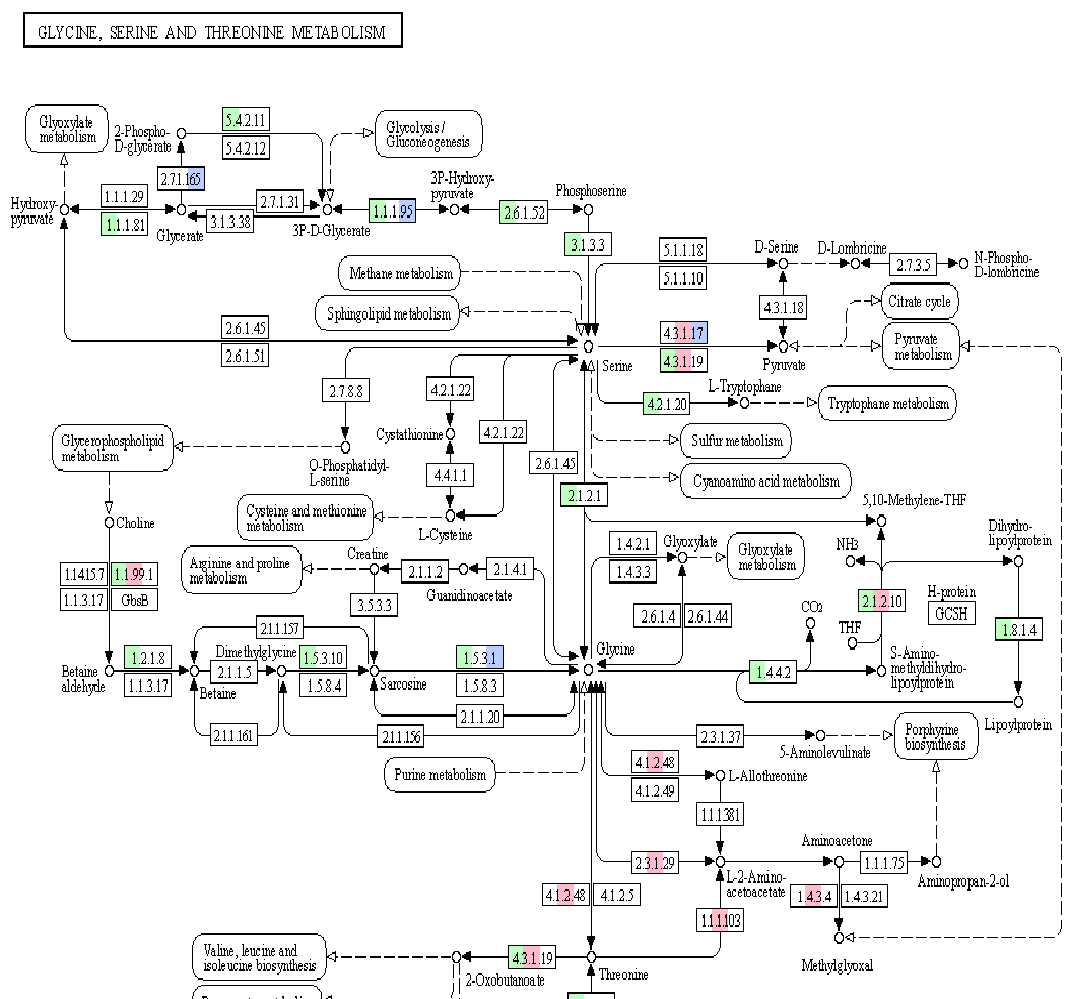


MM; Green, Bacteroide; Pink, With no change in expression; Purple. Highlighting, when two colors are in the enzymatic reaction, it means there are two isozymes for this enzymatic step.

Pathway 5


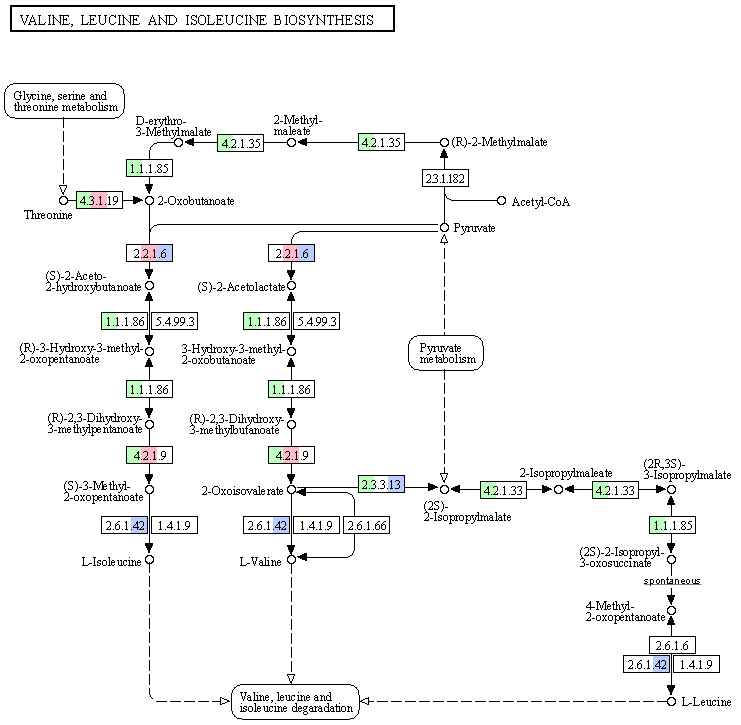


MM; Green, Bacteroide; Pink, With no change in expression; Purple. Highlighting, when two colors are in the enzymatic reaction, it means there are two isozymes for this enzymatic step.

Pathway 6


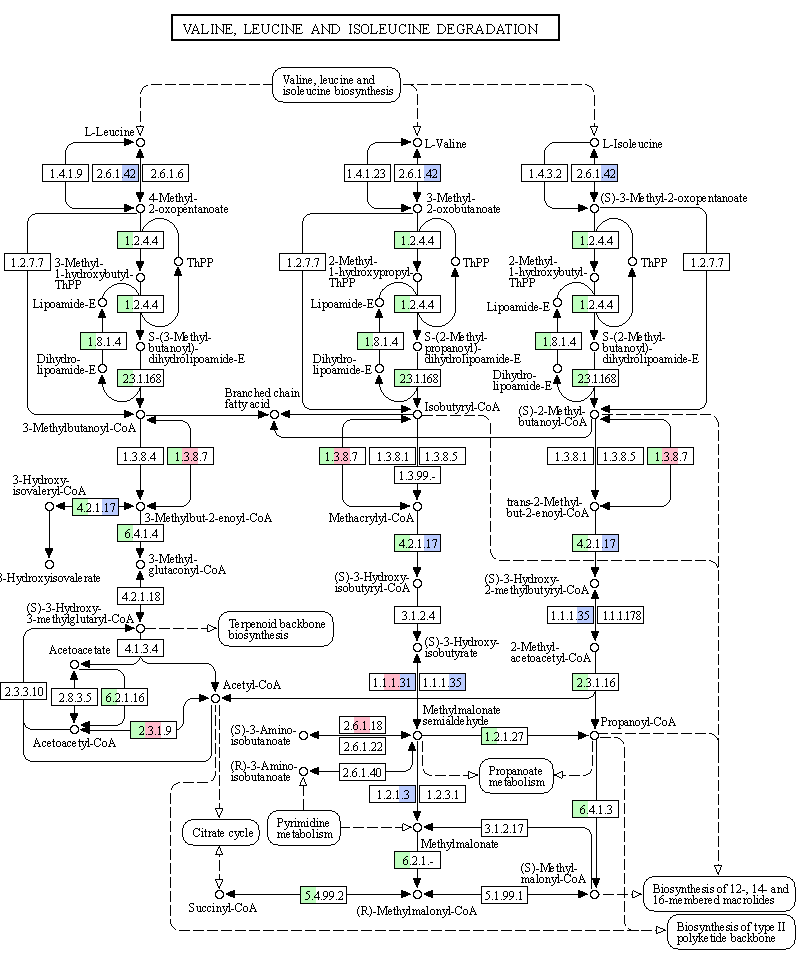


MM; Green, Bacteroide; Pink, With no change in expression; Purple. Highlighting, when two colors are in the enzymatic reaction, it means there are two isozymes for this enzymatic step.

Pathway 7


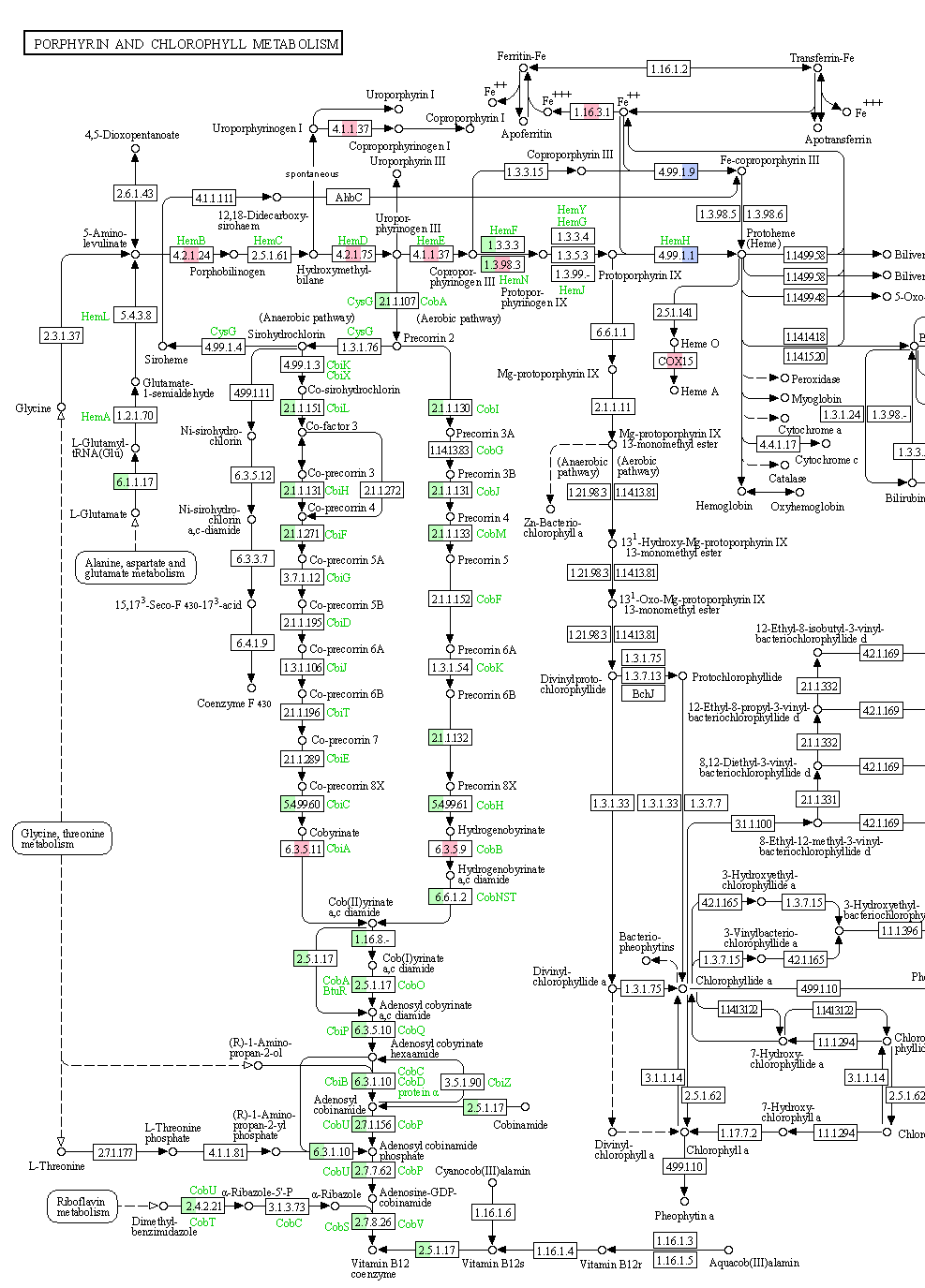


MM; Green, Bacteroide; Pink, With no change in expression; Purple. Highlighting, when two colors are in the enzymatic reaction, it means there are two isozymes for this enzymatic step.

Pathway 8


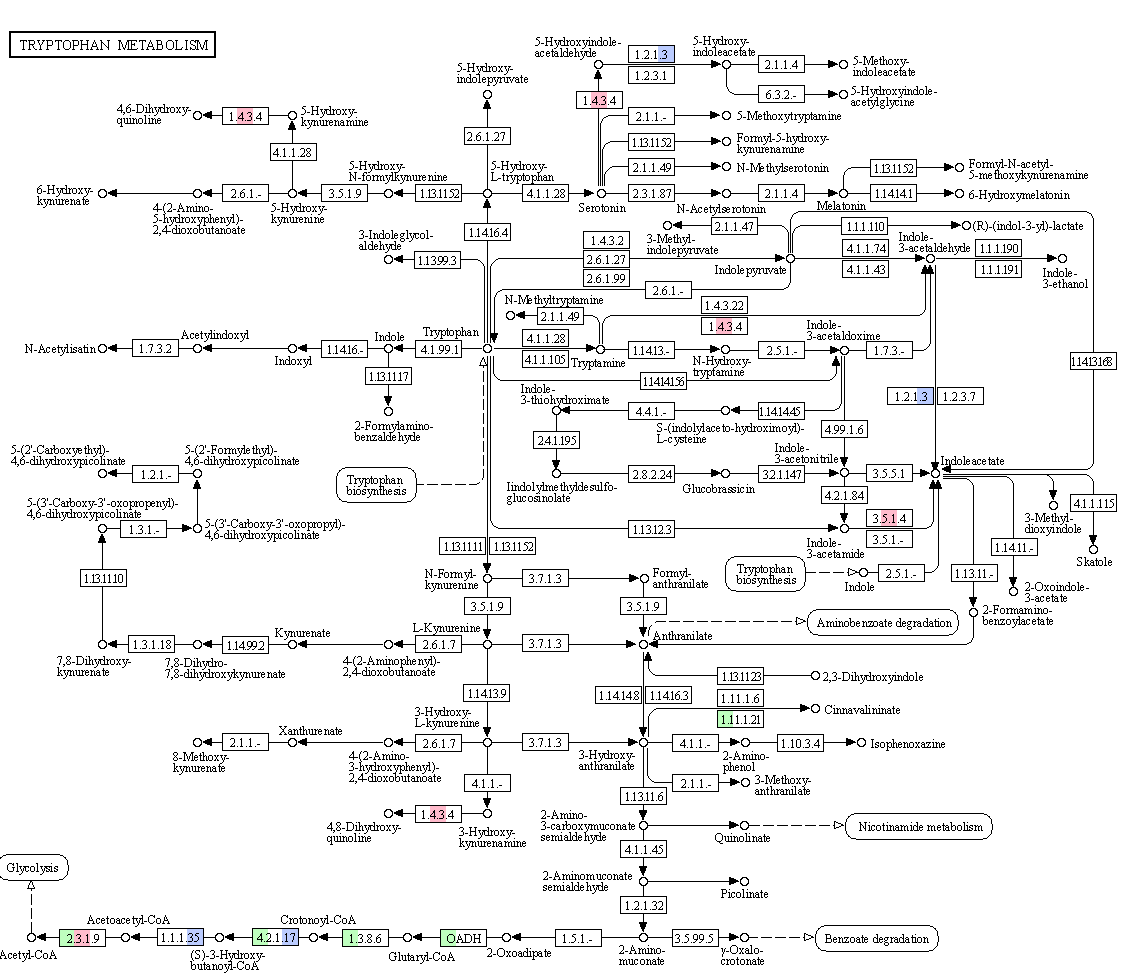


MM; Green, Bacteroide; Pink, With no change in expression; Purple. Highlighting, when two colors are in the enzymatic reaction, it means there are two isozymes for this enzymatic step.

Pathway 9


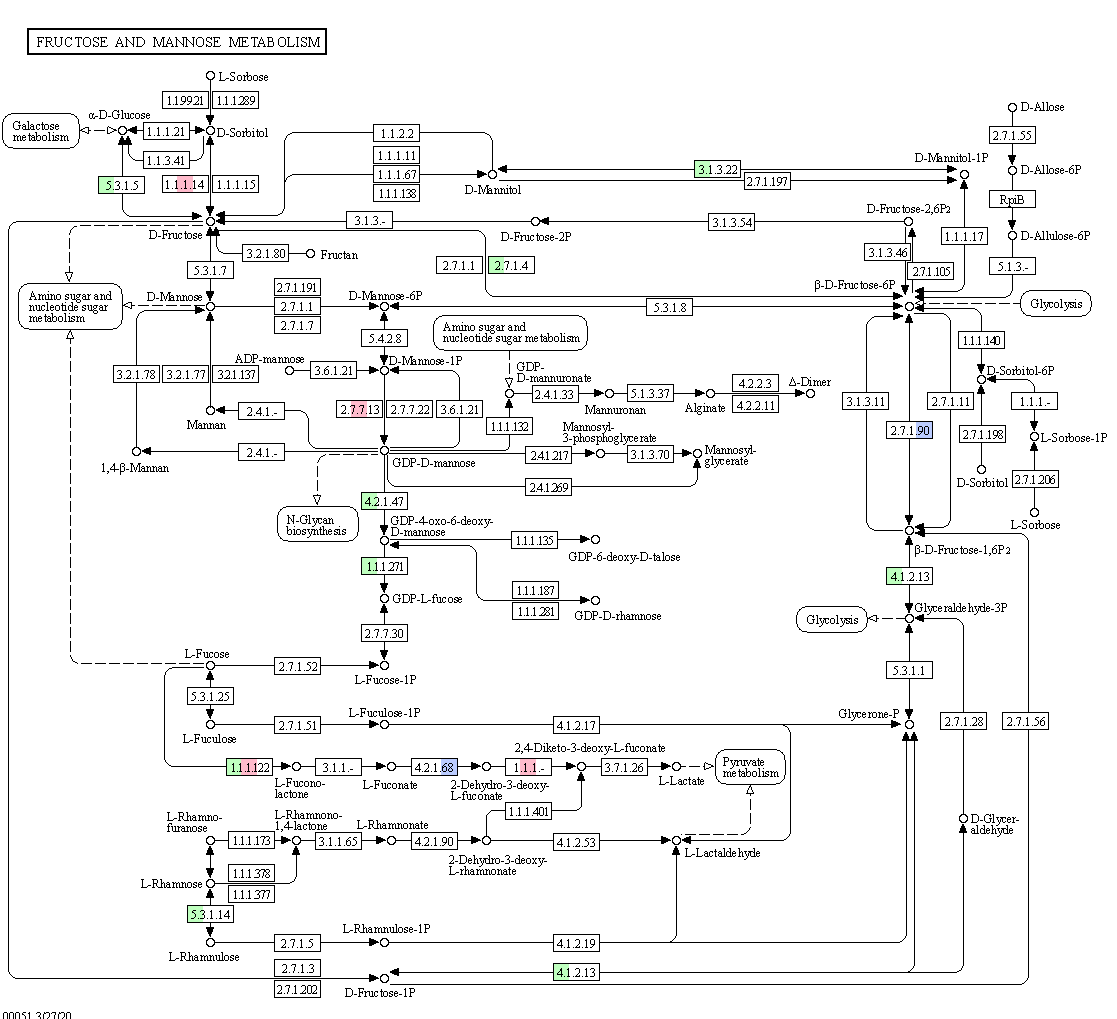


MM; Green, Bacteroide; Pink, With no change in expression; Purple. Highlighting, when two colors are in the enzymatic reaction, it means there are two isozymes for this enzymatic step.

Pathway 10


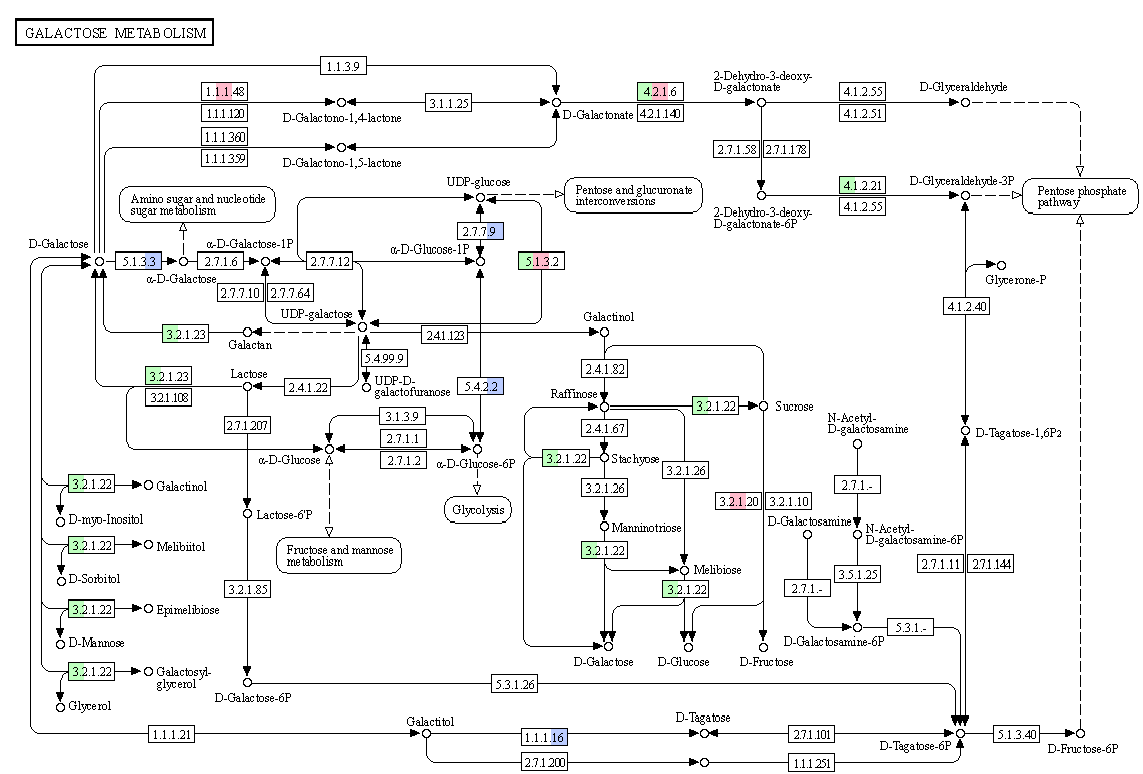


MM; Green, Bacteroide; Pink, With no change in expression; Purple. Highlighting, when two colors are in the enzymatic reaction, it means there are two isozymes for this enzymatic step.

Pathway 11


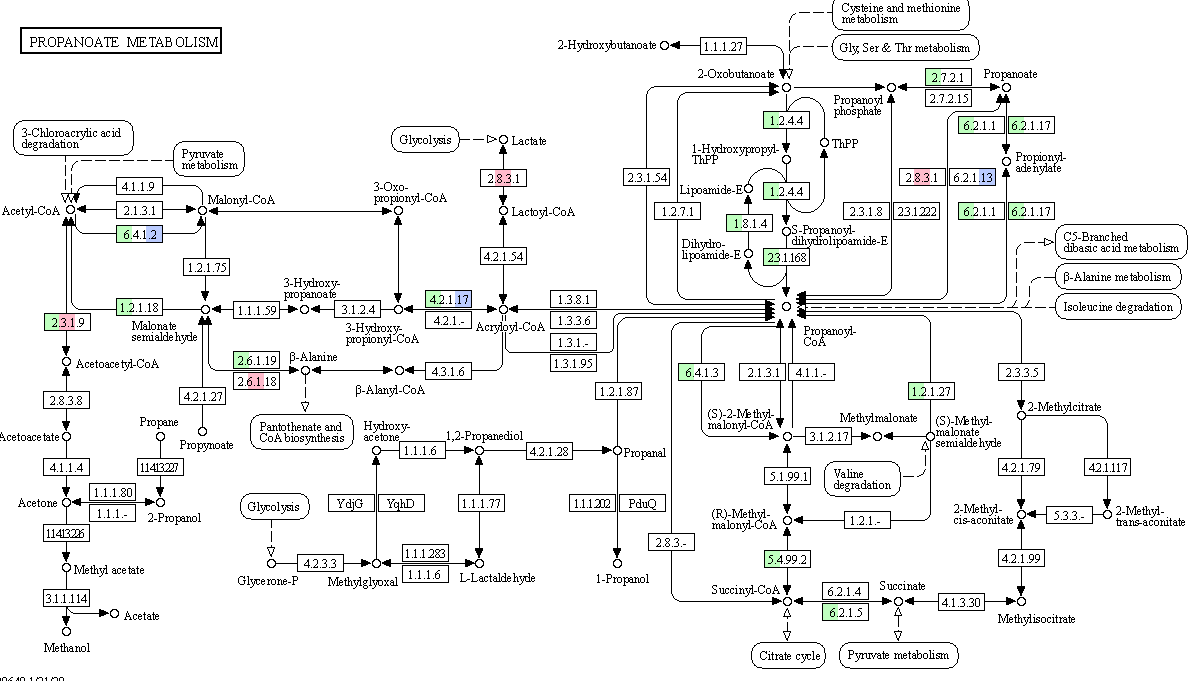


MM; Green, Bacteroide; Pink, With no change in expression; Purple. Highlighting, when two colors are in the enzymatic reaction, it means there are two isozymes for this enzymatic step.

Pathway 12


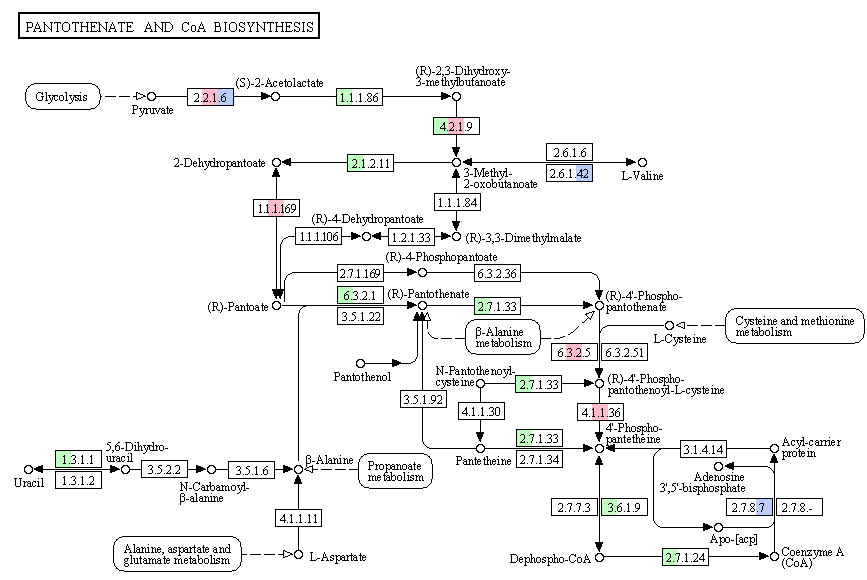


MM; Green, Bacteroide; Pink, With no change in expression; Purple. Highlighting, when two colors are in the enzymatic reaction, it means there are two isozymes for this enzymatic step.

Pathway 13


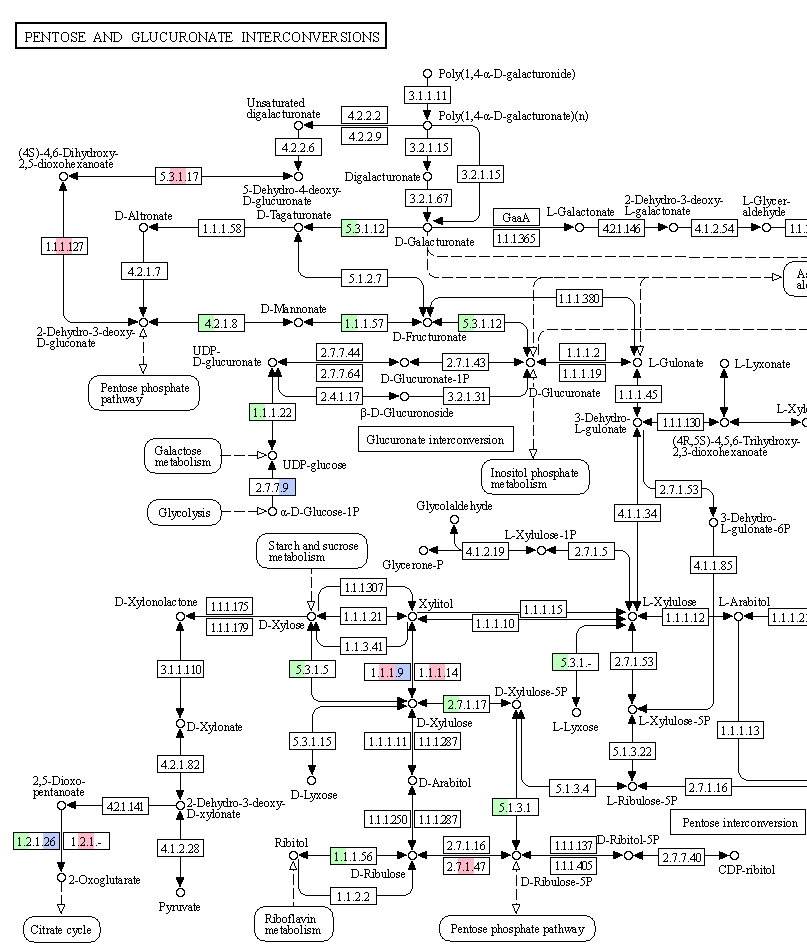


MM; Green, Bacteroide; Pink, With no change in expression; Purple. Highlighting, when two colors are in the enzymatic reaction, it means there are two isozymes for this enzymatic step.

Pathway 14


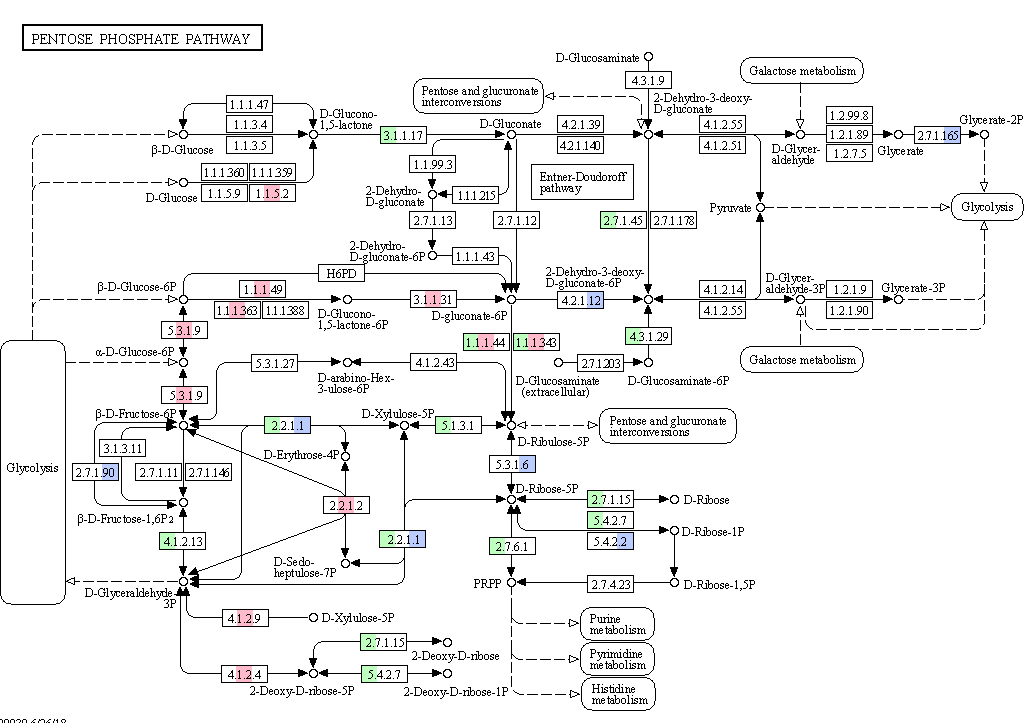


MM; Green, Bacteroide; Pink, With no change in expression; Purple. Highlighting, when two colors are in the enzymatic reaction, it means there are two isozymes for this enzymatic step.

Pathway 15


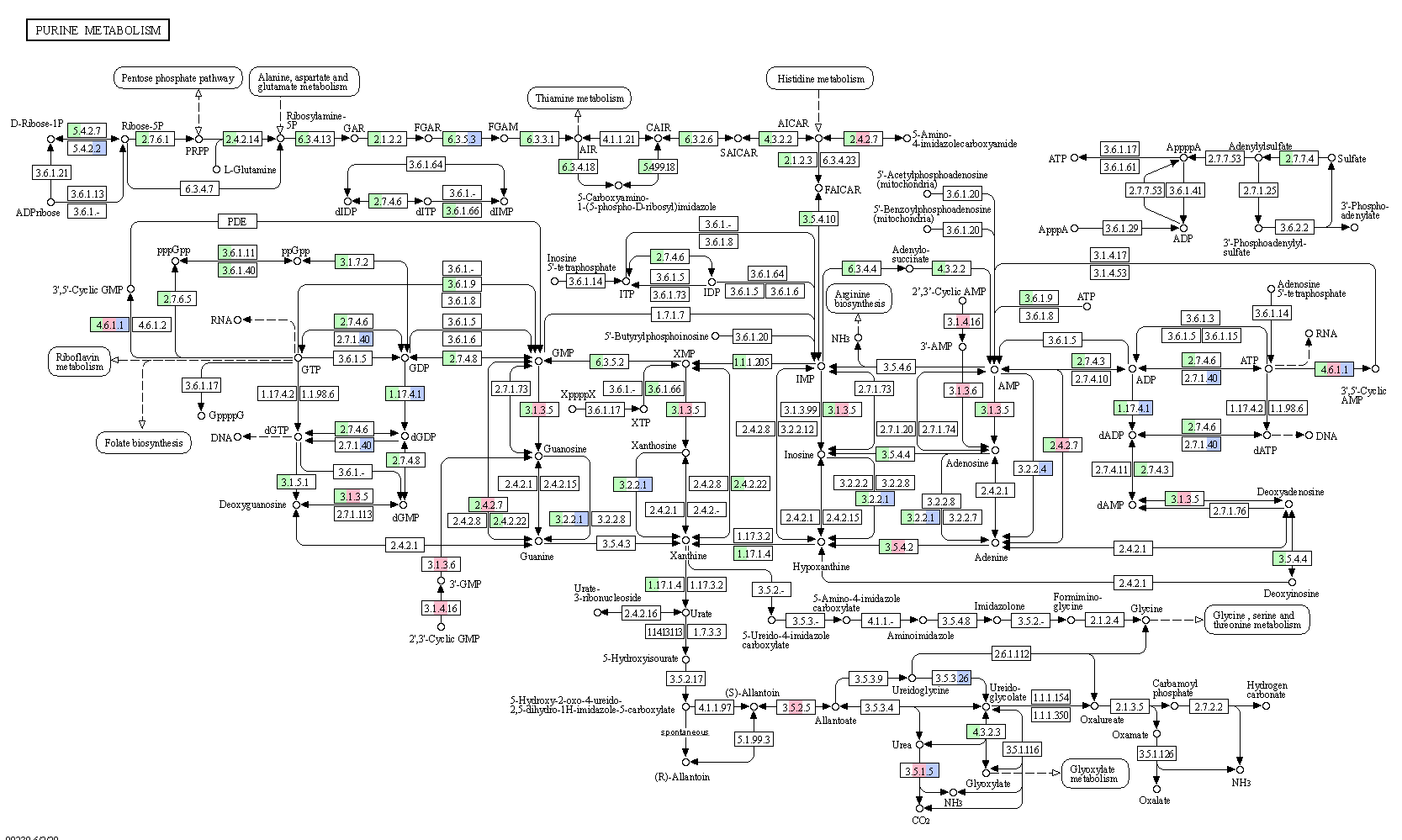


MM; Green, Bacteroide; Pink, With no change in expression; Purple. Highlighting, when two colors are in the enzymatic reaction, it means there are two isozymes for this enzymatic step.

Pathway 16


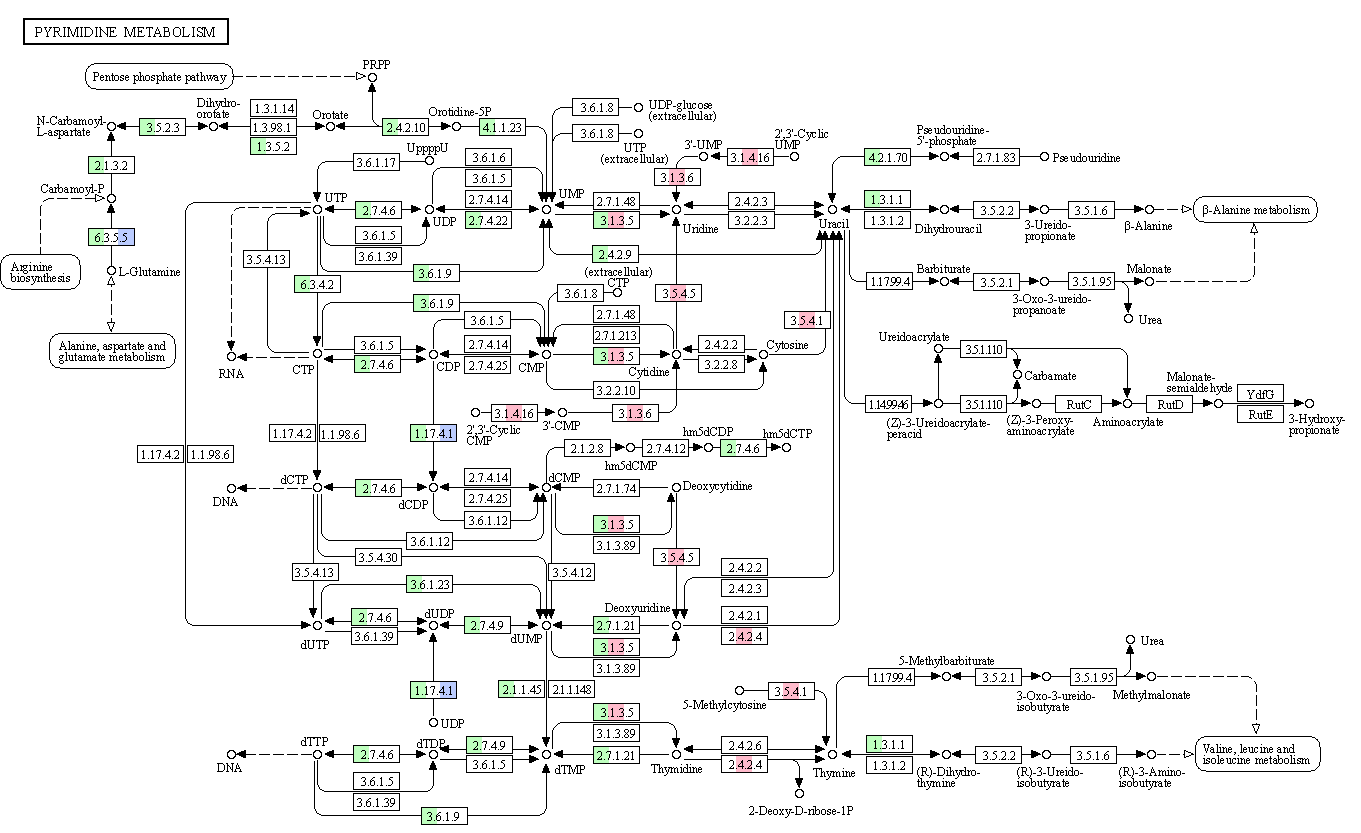


MM; Green, Bacteroide; Pink, With no change in expression; Purple. Highlighting, when two colors are in the enzymatic reaction, it means there are two isozymes for this enzymatic step.

Pathway 17

MM; Green, Bacteroide; Pink, With no change in expression; Purple


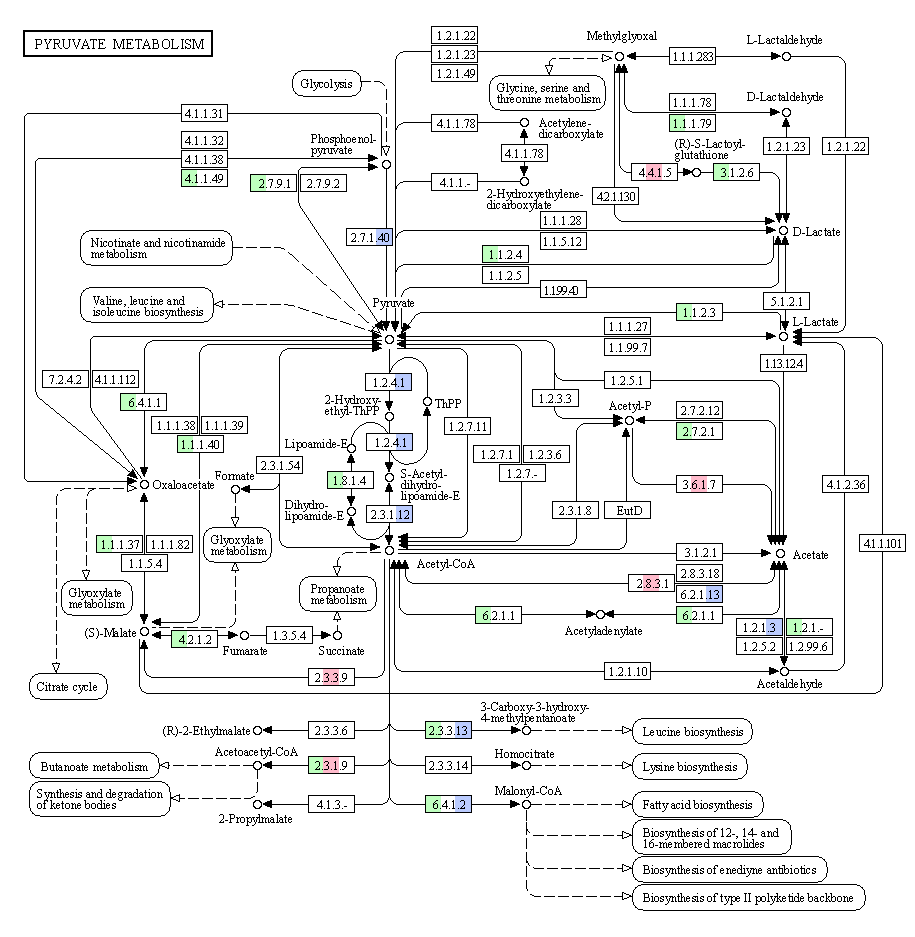


MM; Green, Bacteroide; Pink, With no change in expression; Purple. Highlighting, when two colors are in the enzymatic reaction, it means there are two isozymes for this enzymatic step.

Pathway 18


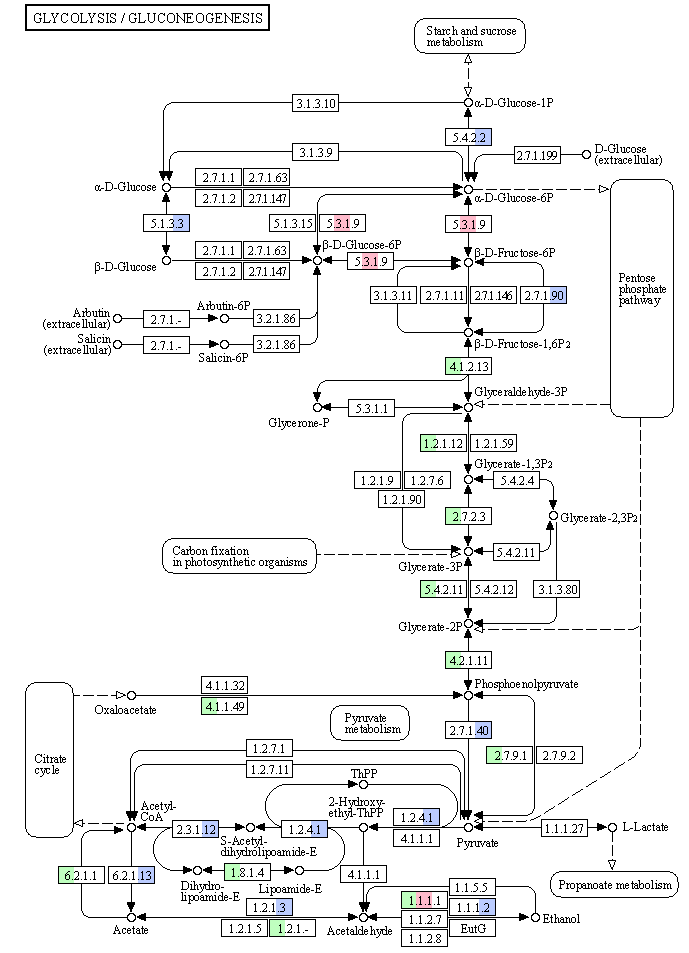


MM; Green, Bacteroide; Pink, With no change in expression; Purple. Highlighting, when two colors are in the enzymatic reaction, it means there are two isozymes for this enzymatic step.

Pathway 19


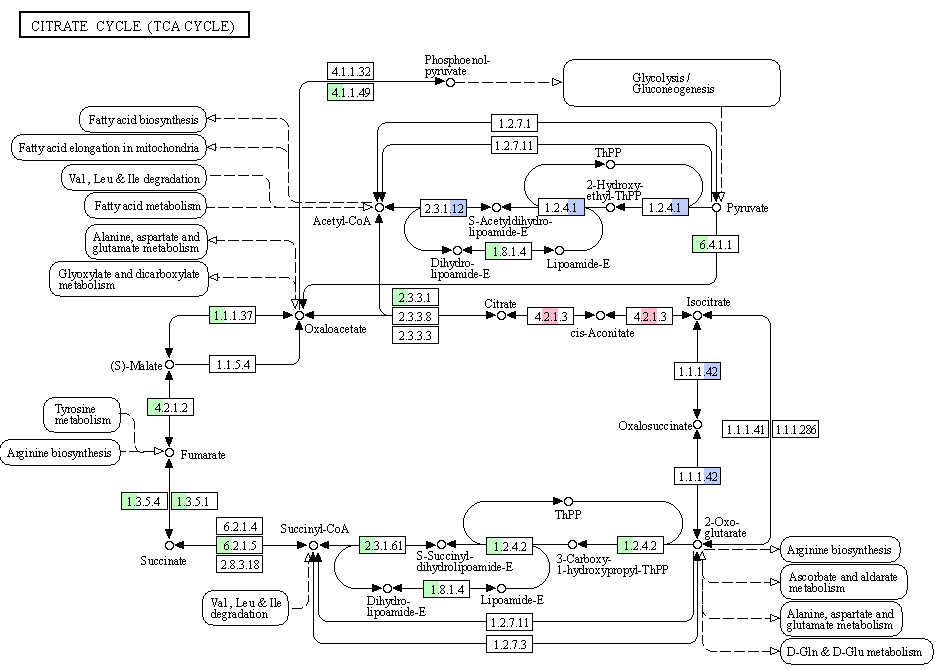


MM; Green, Bacteroide; Pink, With no change in expression; Purple. Highlighting, when two colors are in the enzymatic reaction, it means there are two isozymes for this enzymatic step.

Pathway 20


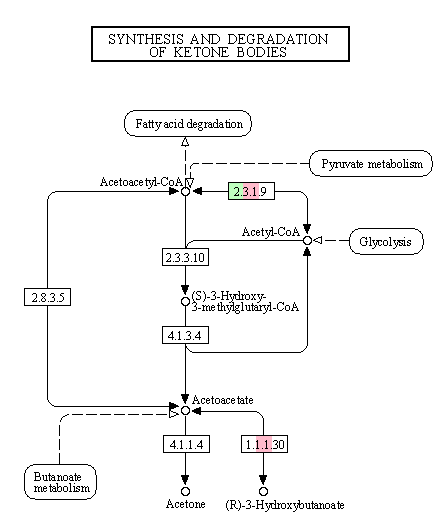


MM; Green, Bacteroide; Pink, With no change in expression; Purple. Highlighting, when two colors are in the enzymatic reaction, it means there are two isozymes for this enzymatic step.

Pathway 21


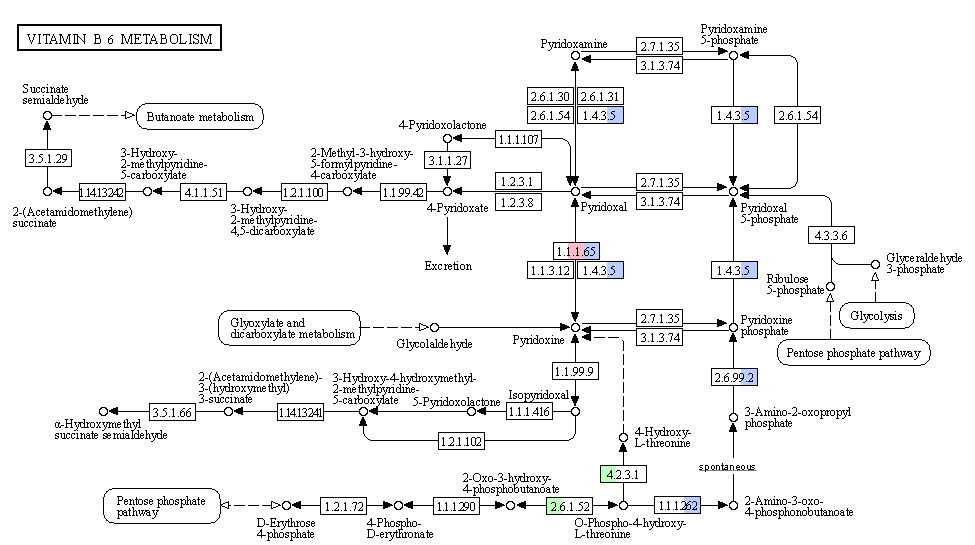


MM; Green, Bacteroide; Pink, With no change in expression; Purple. Highlighting, when two colors are in the enzymatic reaction, it means there are two isozymes for this enzymatic step.

Pathway 22


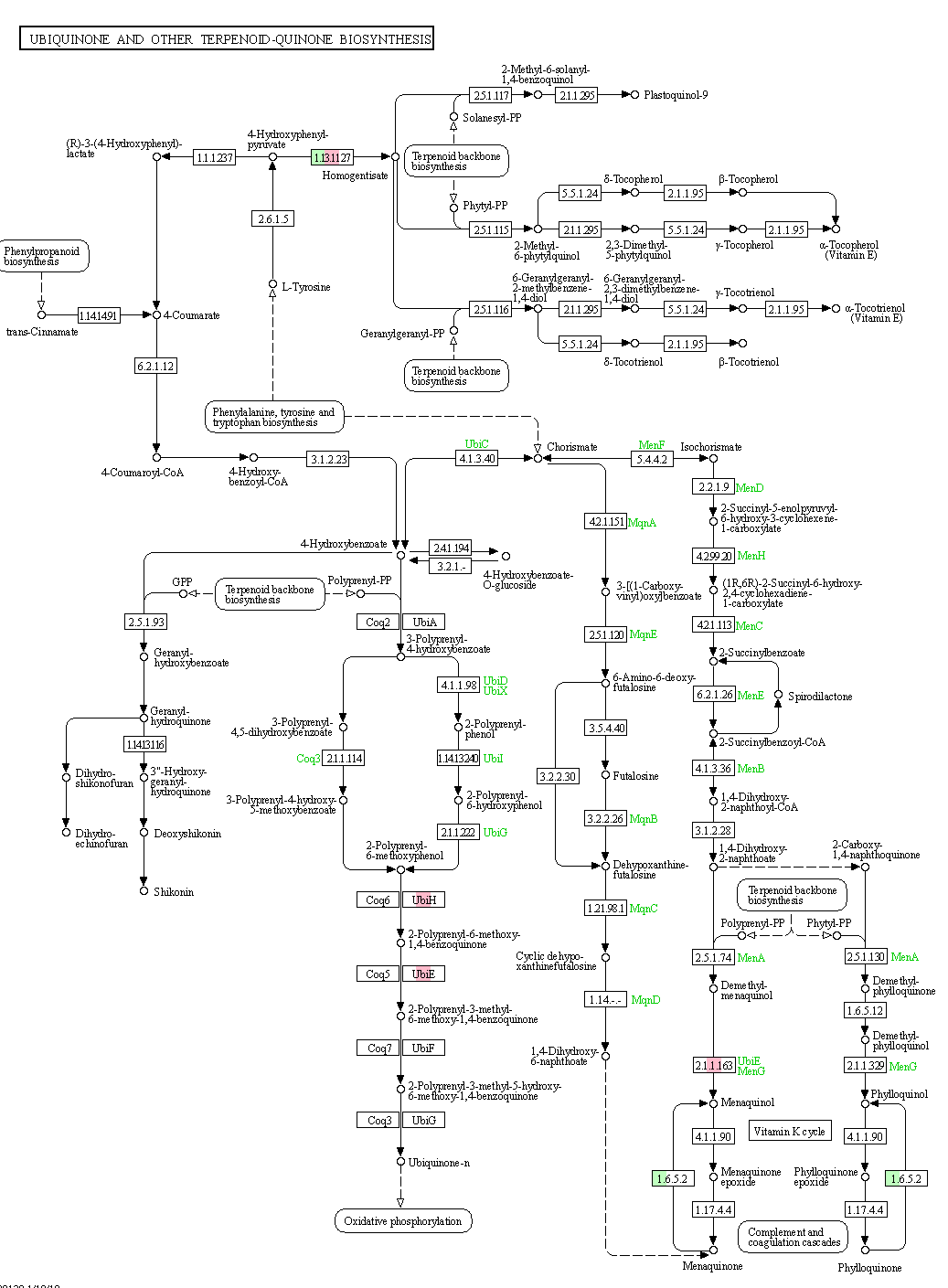


MM; Green, Bacteroide; Pink, With no change in expression; Purple. Highlighting, when two colors are in the enzymatic reaction, it means there are two isozymes for this enzymatic step.

Pathway 23


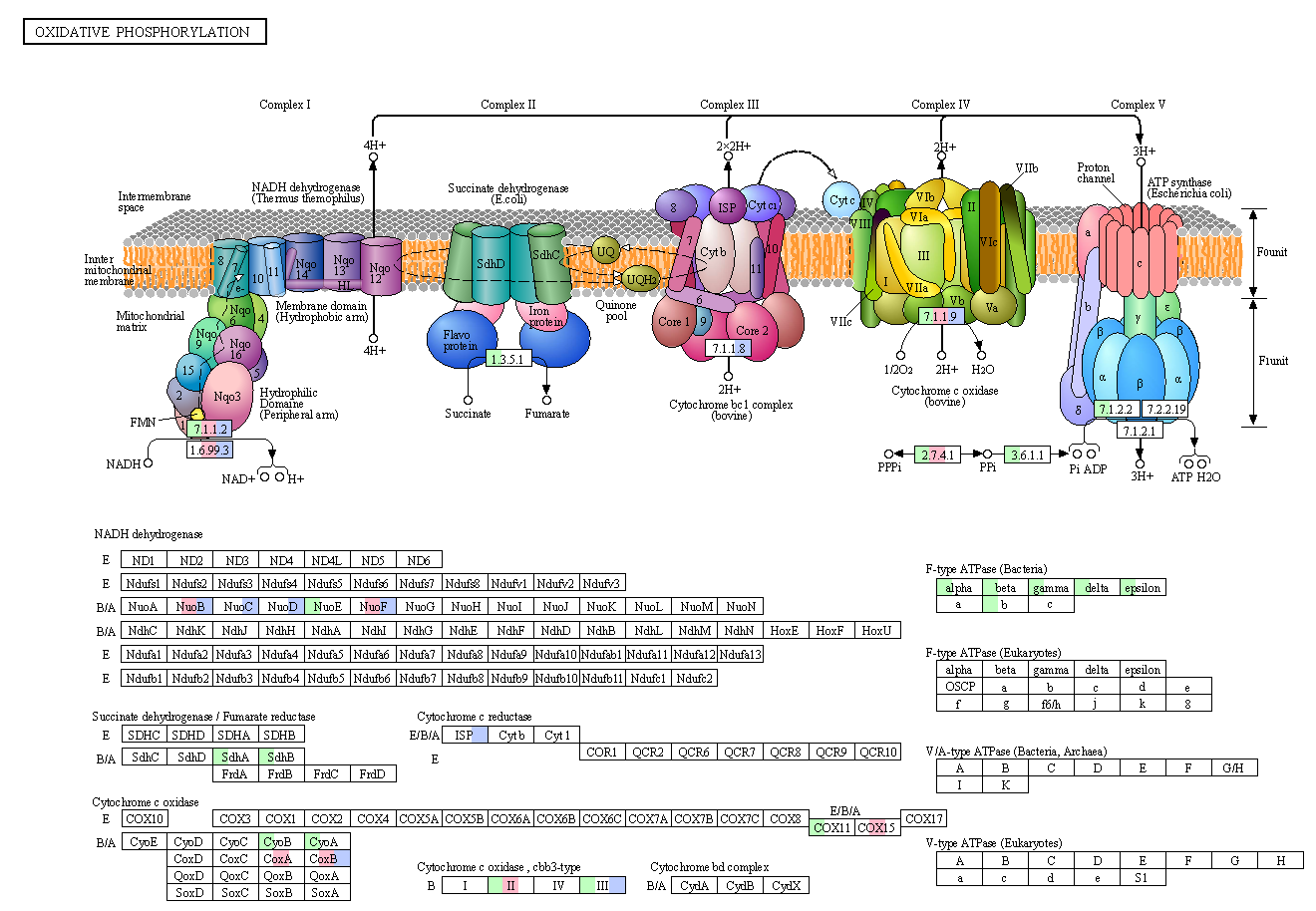


MM; Green, Bacteroide; Pink, With no change in expression; Purple. Highlighting, when two colors are in the enzymatic reaction, it means there are two isozymes for this enzymatic step.

Pathway 24


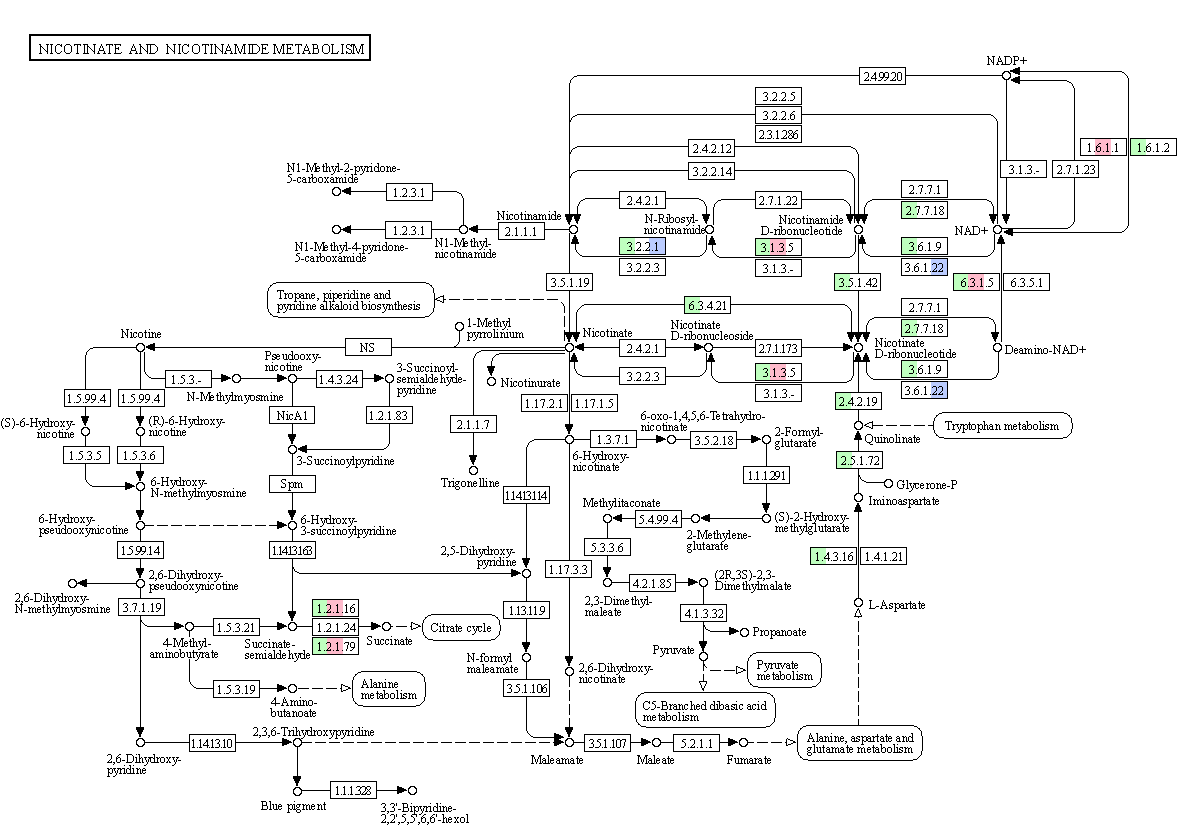


MM; Green, Bacteroide; Pink, With no change in expression; Purple. Highlighting, when two colors are in the enzymatic reaction, it means there are two isozymes for this enzymatic step.

Pathway 25


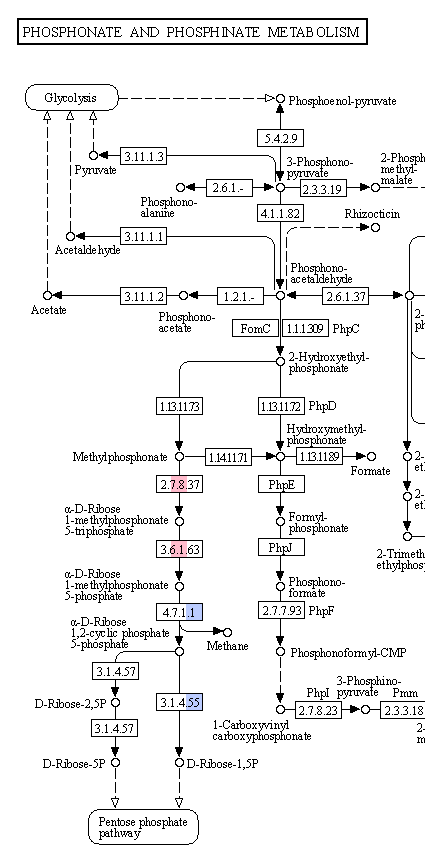


MM; Green, Bacteroide; Pink, With no change in expression; Purple. Highlighting, when two colors are in the enzymatic reaction, it means there are two isozymes for this enzymatic step.

Pathway 26


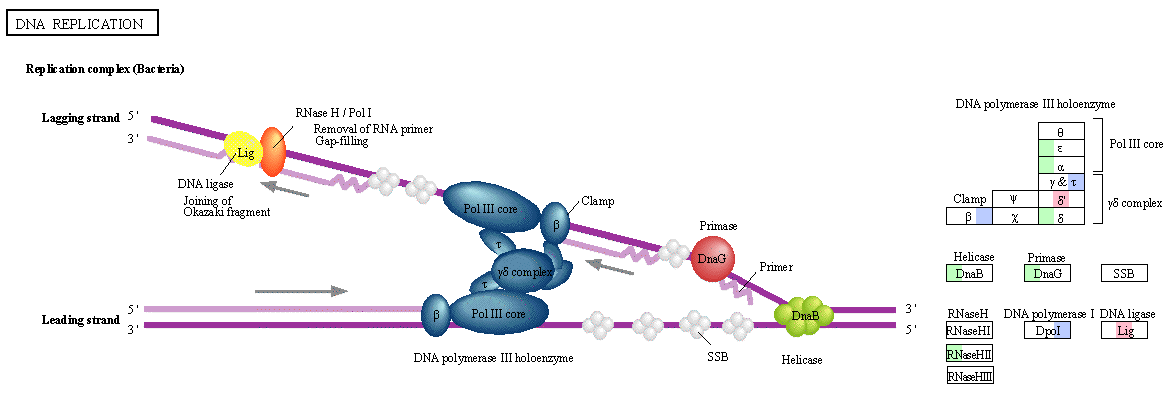


MM; Green, Bacteroide; Pink, With no change in expression; Purple. Highlighting, when two colors are in the enzymatic reaction, it means there are two isozymes for this enzymatic step.

Pathway 27


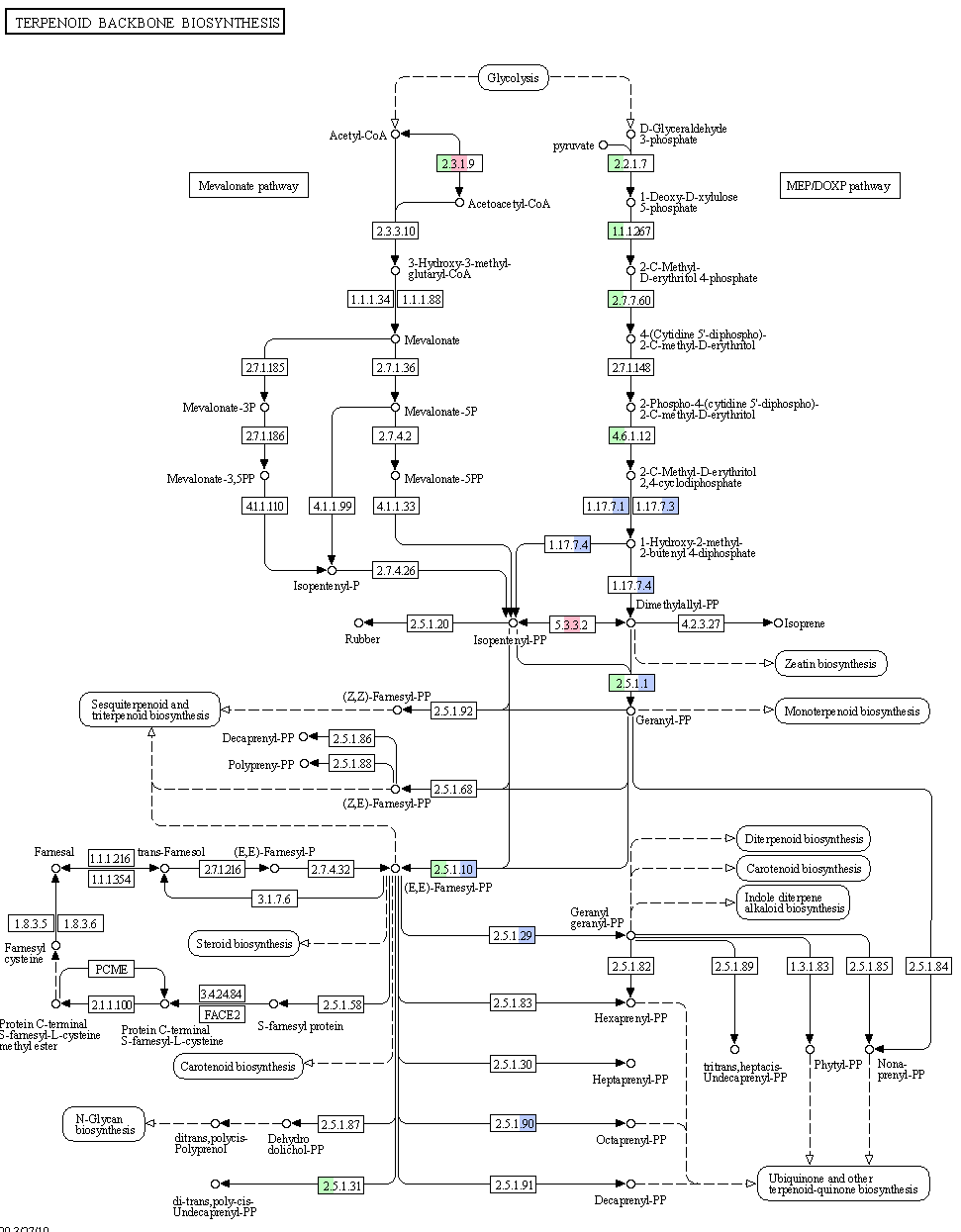


MM; Green, Bacteroide; Pink, With no change in expression; Purple. Highlighting, when two colors are in the enzymatic reaction, it means there are two isozymes for this enzymatic step.

Pathway 28

Environmental metabolism


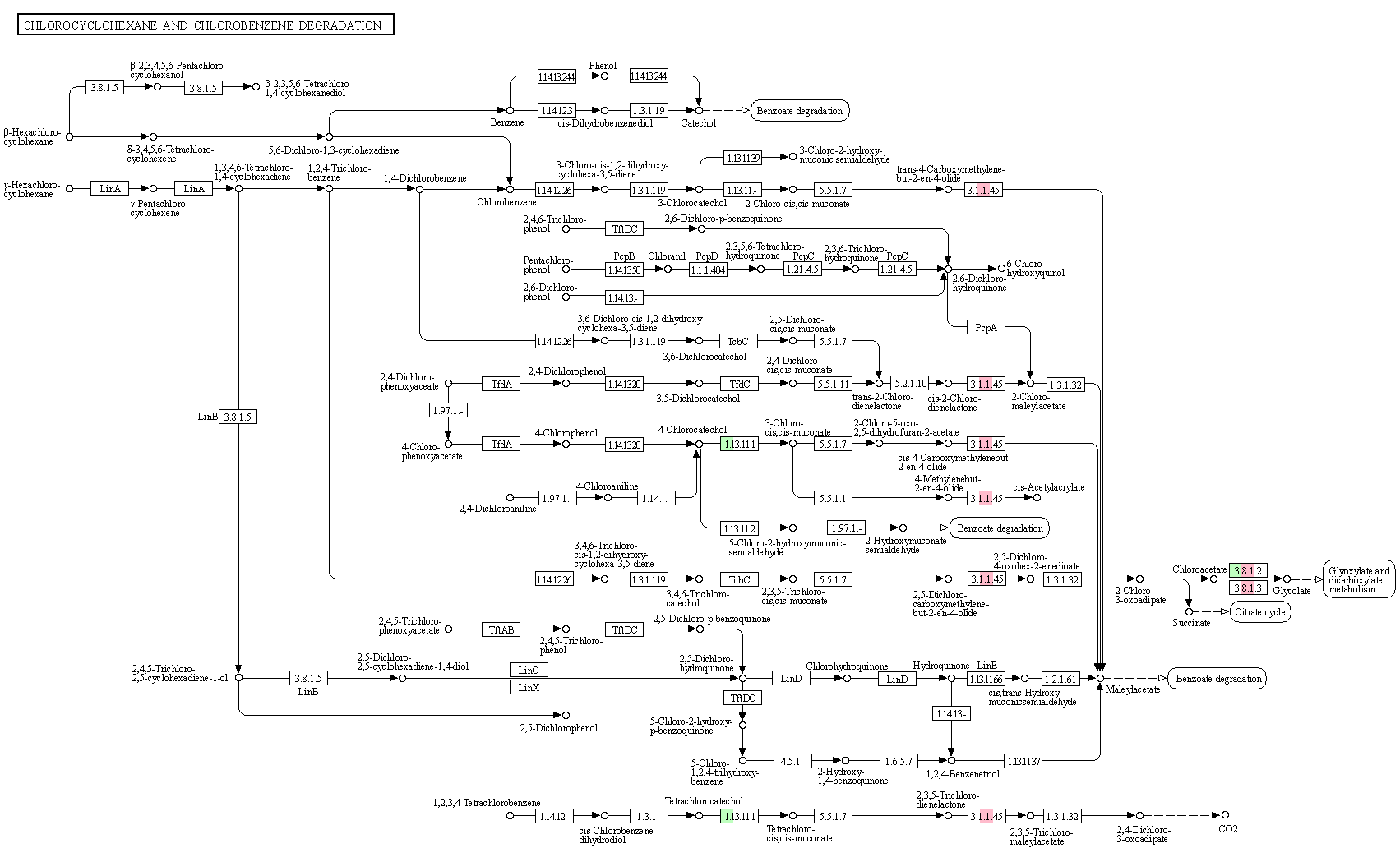


MM; Green, Bacteroide; Pink, With no change in expression; Purple. Highlighting, when two colors are in the enzymatic reaction, it means there are two isozymes for this enzymatic step.

Pathway 29


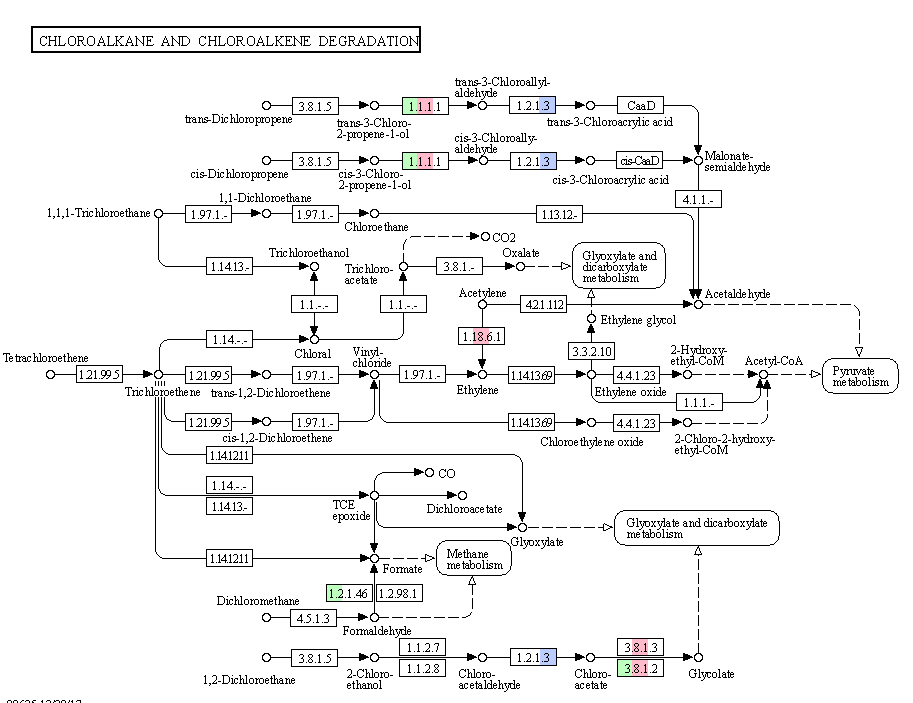


MM; Green, Bacteroide; Pink, With no change in expression; Purple. Highlighting, when two colors are in the enzymatic reaction, it means there are two isozymes for this enzymatic step.

Pathway 30


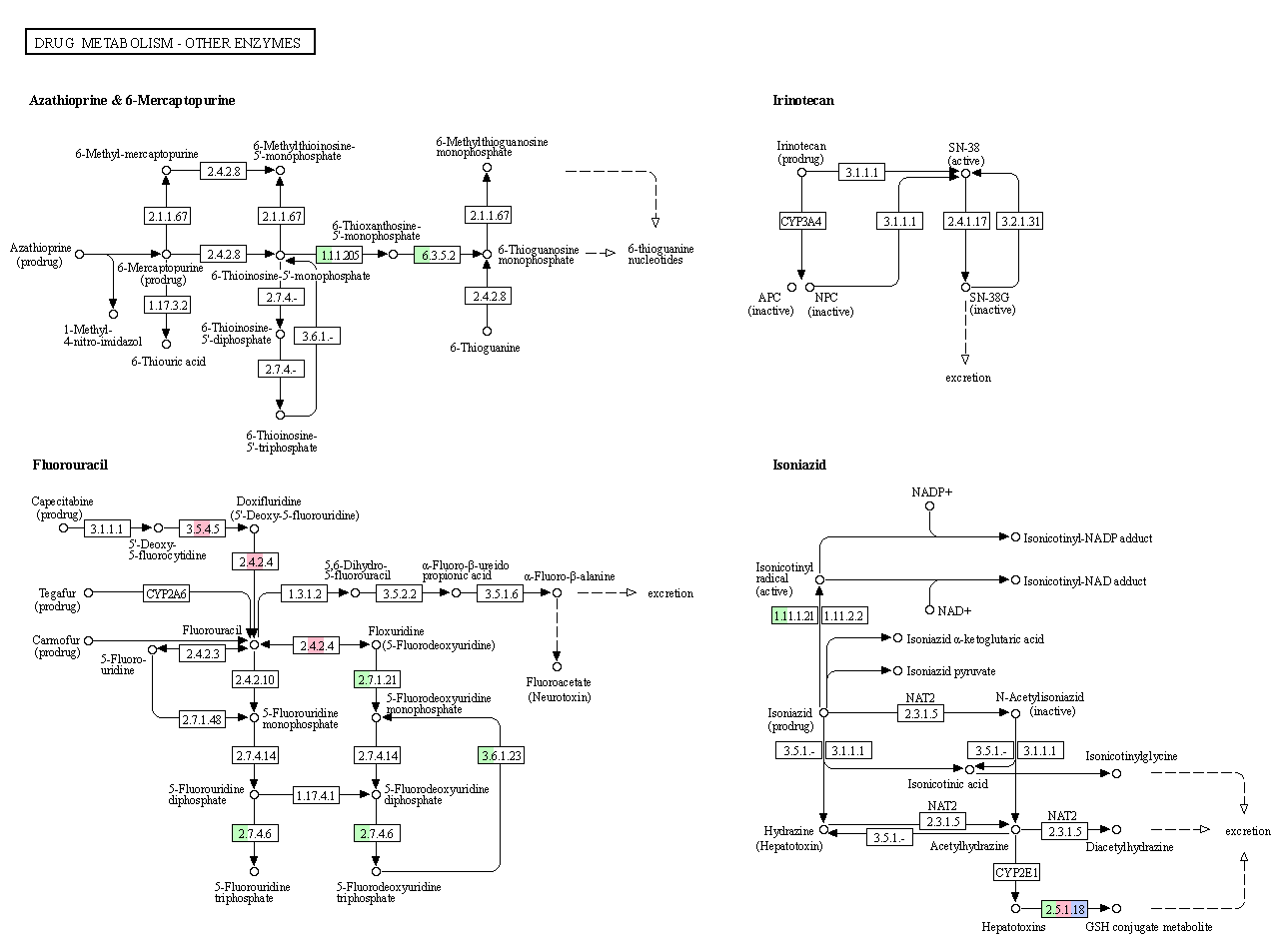


MM; Green, Bacteroide; Pink, With no change in expression; Purple. Highlighting, when two colors are in the enzymatic reaction, it means there are two isozymes for this enzymatic step.

Pathway 31


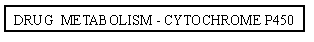


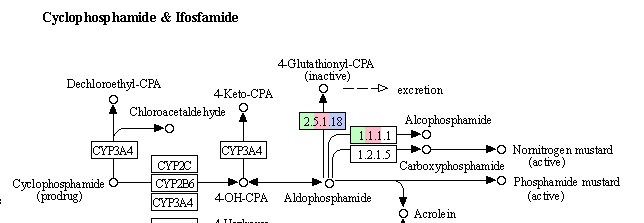


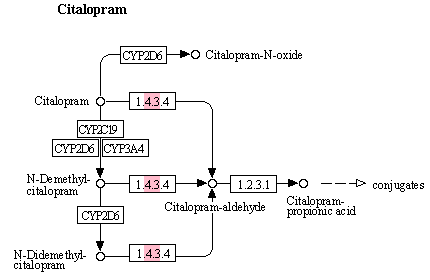


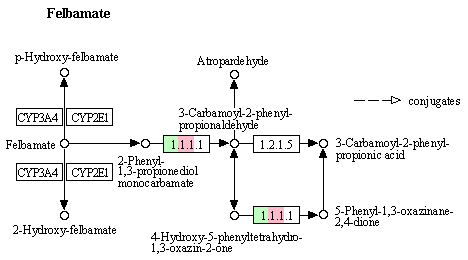


MM; Green, Bacteroide; Pink, With no change in expression; Purple. Highlighting, when two colors are in the enzymatic reaction, it means there are two isozymes for this enzymatic step.

Pathway 32


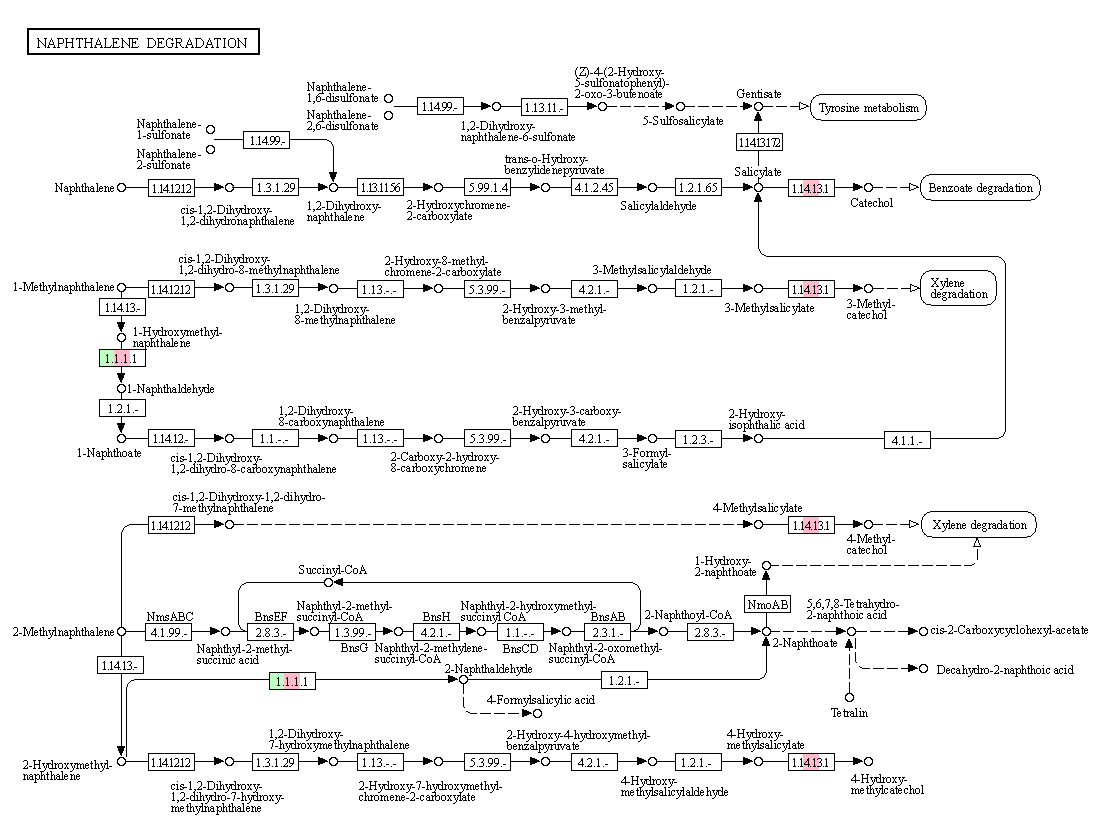


MM; Green, Bacteroide; Pink, With no change in expression; Purple. Highlighting, when two colors are in the enzymatic reaction, it means there are two isozymes for this enzymatic step.

Pathway 33


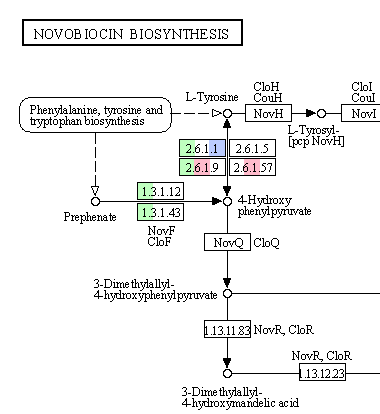


MM; Green, Bacteroide; Pink, With no change in expression; Purple. Highlighting, when two colors are in the enzymatic reaction, it means there are two isozymes for this enzymatic step.

Pathway 34


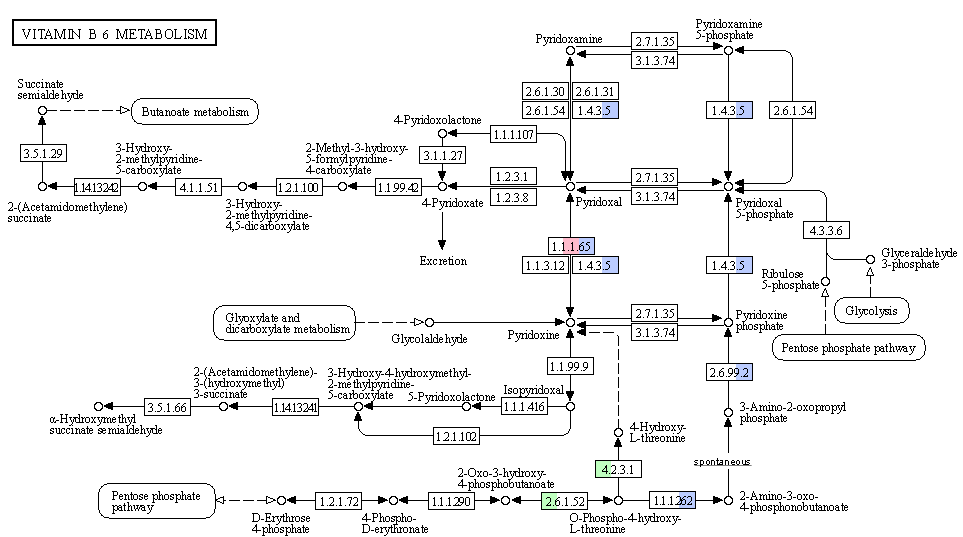


MM; Green, Bacteroide; Pink, With no change in expression; Purple. Highlighting, when two colors are in the enzymatic reaction, it means there are two isozymes for this enzymatic step.

Pathway 35


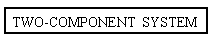


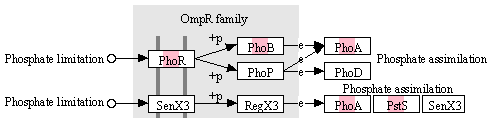


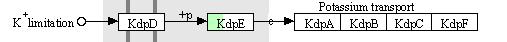


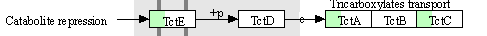


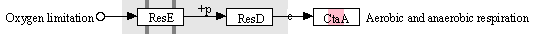


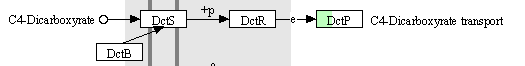


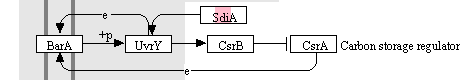


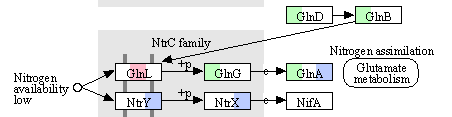


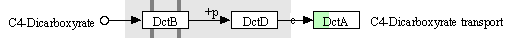


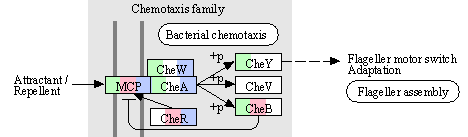


MM; Green, Bacteroide; Pink, With no change in expression; Purple. Highlighting, when two colors are in the enzymatic reaction, it means there are two isozymes for this enzymatic step.

Pathway 36

**ABC TRANSPORTERS**


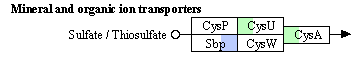


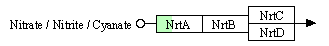


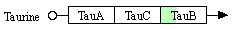


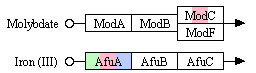


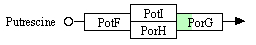


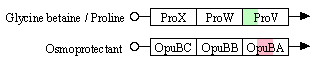


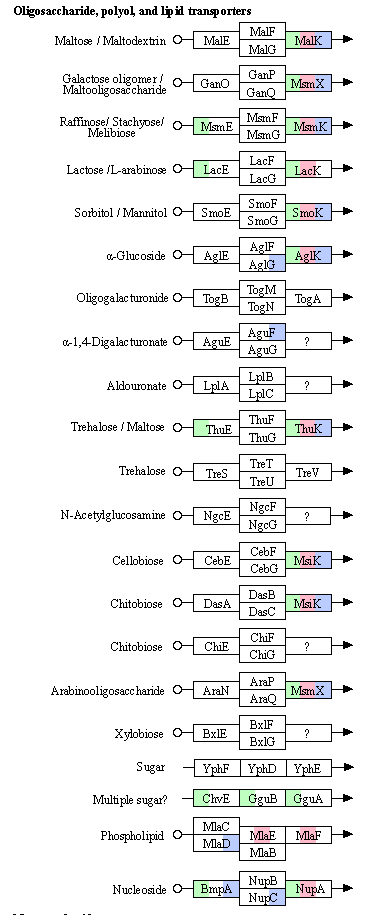


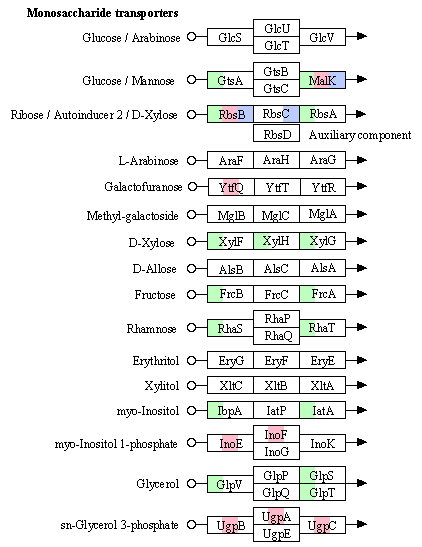


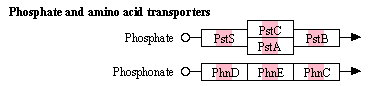


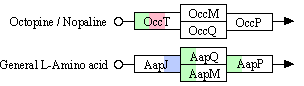


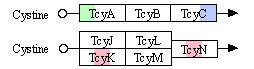


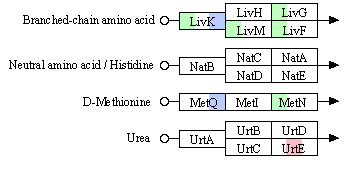


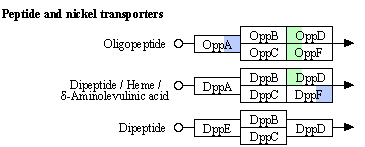


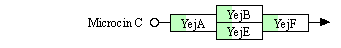


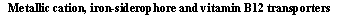


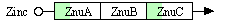


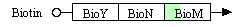


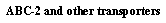


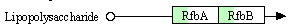


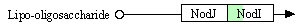


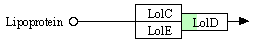


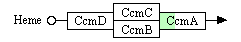


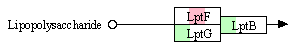


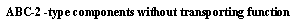


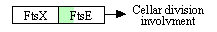


MM; Green, Bacteroide; Pink, With no change in expression; Purple. Highlighting, when two colors are in the enzymatic reaction, it means there are two isozymes for this enzymatic step.

Pathway 37
